# Brazilian Kayabi Indian accessions of peanut, *Arachis hypogaea* (Fabales, Fabaceae): origin, diversity and evolution

**DOI:** 10.1590/1678-4685-GMB-2019-0418

**Published:** 2020-11-06

**Authors:** Eliza Fabricio de Melo Bellard do Nascimento, Soraya Cristina de Macedo Leal-Bertioli, David John Bertioli, Carolina Chavarro, Fábio Oliveira Freitas, Márcio de Carvalho Moretzsohn, Patricia Messenberg Guimarães, José Francisco Montenegro Valls, Ana Claudia Guerra de Araujo

**Affiliations:** ^1^ Universidade de Brasília, Instituto de Ciências Biológicas, Campus Darcy Ribeiro, Brasília, DF, Brazil.; ^2^ Embrapa Recursos Genéticos e Biotecnologia, Brasília, DF, Brazil.; ^3^ University of Georgia, Center for Applied Genetic Technologies, Athens, GA, USA.

**Keywords:** Chromosome, *in situ* hybridization, morphological plasticity, SNPs, Xingu Indigenous Park

## Abstract

Peanut is a crop of the Kayabi tribe, inhabiting the Xingu Indigenous Park, Brazil. Morphological analysis of Xingu accessions showed variation exceeding that described for cultivated peanuts. This raised questions as to the origin of the Xingu accessions: are they derived from different species, or is their diversity a result of different evolutionary and selection processes? To answer these questions, cytogenetic and genotyping analyses were conducted. The karyotypes of Xingu accessions analyzed are very similar to each other, to an *A. hypogaea* subsp. *fastigiata* accession and to the wild allotetraploid *A. monticola*. The accessions share the number and general morphology of the chromosomes; DAPI^+^ bands; 5S and 45S rDNA loci distribution and a high genomic affinity with *A. duranensis* and *A. ipaënsis* genomic probes. However, the number of CMA_3_^+^ bands differs from those determined for *A. hypogaea* and *A. monticola*, which are also different from each other. SNP genotyping grouped all *Arachis* allotetraploids into four taxonomic groups: Xingu accessions were closer to *A. monticola* and *A. hypogaea* subsp. *hypogaea*. Our data suggests that the morphological diversity within these accessions is not associated with a different origin and can be attributed to morphological plasticity and different selection by the Indian tribes.

## Introduction

Cultivated peanut, *Arachis hypogaea* (Fabales, Fabaceae) is a recent allotetraploid, with an origin estimated between 3,500 and 9,400 years ago (Simpson *et al.*, 2001; Bertioli *et al.*, 2016, 2019). The vast archaeological findings dating to 3,700 years ago certify its relevance in the human diet since the distant past (Pickersgill and Heiser, 1977). It is a South American native oil legume, cultivated worldwide, including in Brazil by local indigenous populations.

The most probable center of origin of the genus *Arachis* is located in central region of Brazil (Valls, 2000). The genus *Arachis* displays a peculiarity that defines all the species: the underground development of seeds, known as geocarpy (Smith, 1950; Krapovickas and Gregory, 1994). The genus comprises mostly wild diploid species (*2n =* 2x = 20), including the progenitor species of the peanut, *A. duranensis* (A genome) and *A. ipaënsis* (B genome). The section *Arachis*, to which *A. hypogaea* belongs, has another allotetraploid species, the wild *A. monticola* (Krapovickas and Gregory, 1994; Valls *et al.*, 2013), with A and B subgenomes, from the same progenitors of peanut (Seijo *et al.*, 2004, 2007; Moretzsohn *et al.*, 2013; Bertioli *et al.*, 2016, 2019). Besides the A and B genomes, section *Arachis* comprises species with D, F, K and G genomes, separated based on morphological, cytological aspects, FISH mapping of rDNA loci and heterochromatin distribution (Stalker, 1991; Robledo and Seijo, 2008, 2010; Silvestri *et al.*, 2015).

The cultivated peanut, being an allotetraploid, is not easily crossable to its wild diploid relatives. This has prevented gene flow between the cultigen and its wild diploid relatives and restricted its genetic variability (Kochert *et al.*, 1991, 1996; Stalker and Simpson, 1995; Moretzsohn *et al.*, 2004; Bertioli *et al.*, 2016, 2019). However, the variability in morphological and agronomical aspects is surprisingly high; two subspecies are recognized, *hypogaea* and *fastigiata*, divided into a total of six botanical varieties (Krapovickas and Gregory, 1994). The various processes of selection under cultivation produced variation, bushy and erect growth habits, variable numbers of seeds per pod, size and color of seeds, among others (Krapovickas and Gregory, 1994).

Peanuts are the fifth most produced oilseed in the world (USDA-FAS, 2019). In Brazil, commercial cultivation is mostly concentrated in the state of Sao Paulo, but importantly, it is also grown in small-scale for subsistence, including by some Brazilian indigenous tribes. One major ethnic group maintaining and using peanut accessions is the Kayabi tribe, inhabiting the Xingu Indigenous Park, situated at the northeast area of Mato Grosso Brazilian state. They have inhabited this region since the early sixties, when they were moved there from further west, near Bolivia and Peru (Novaes, 1985).

The Kayabi people have a complex system of agriculture that includes peanut as one of the main sources of food (Freitas *et al.*, 2007). As such, they are important caretakers of their accessions and guardians of distinct genetic resources. Because of this, scientific expeditions have been organized to identify and collect the local peanut accessions, as well as to learn about their agriculture practices (Freitas *et al.*, 2007).

The characterization of these accessions from Xingu Indigenous Park and surrounding areas using taxonomic descriptors has shown morphological characteristics that surpassed the variation described for the six botanical varieties of *A. hypogaea*, as observed in the shape, size, texture and color of the pods and seeds (Suassuna *et al.*, 2016). These differences raised questions about the possible contribution of progenitor species other than *A. duranensis* and *A. ipaënsis* in the origin of some Xingu accessions. In fact, it is reasonable to consider the possible participation of other diploid *Arachis* species in the origin of the accessions, due to the occurrence in the region of the Central Brazil of wild species that share some aspects of the morphology of the pods with the Xingu accessions, specially *A. stenosperma*, which is also cultivated by indigenous tribes in Brazil that has pods with a thin smooth shell, and *A. magna*, very closely related to *A. ipaënsis*, with pods showing a moderate longitudinal prominence (Simpson *et al.*, 2001; Freitas *et al.*, 2007; Koppolu *et al.*, 2010; Moretzsohn *et al.*, 2013). However, an alternative possibility is that the origin of the Xingu Park accessions is the same as *A. hypogaea* and *A. monticola*, and their isolation, selection and genetic recombination has produced morphological features not present in accessions from other areas.

Aiming to better characterize the accessions from Xingu Park, herein is presented a cytogenetic and SNP genotyping study of some *A. hypogaea* types from Xingu, and compared to peanut cultivars, *A. monticola* and four wild diploid species.

## Material and Methods

### Plant material

Three accessions of *A. hypogaea* (AB genome) collected at the Kayabi indigenous villages Guarujá and Ilha Grande, in the Xingu Indigenous Park (Mato Grosso, Brazil), accessions Of 115, Of 120 and Of 126; *A. hypogaea* subsp. *fastigiata* var. *fastigiata* ‘IAC Tatu-ST’ (AB genome) and the wild allotetraploid *A. monticola* V 14165 (AB genome) were used for cytogenetic analysis. The diploid species, *A. duranensis* V 14167 (A genome), *A. stenosperma* V 10309 (A genome), *A. ipaënsis* K 30076 (B genome) and *A. magna* K 30097 (B genome) were used in the cytogenetic analysis as source of genomic DNA for genomic *in situ* hybridization (GISH) probes. For the single nucleotide polymorphism (SNP) genotyping, five Xingu Park accessions, 22 accessions of *A. hypogaea*, and five of *A. monticola* were used (Table 1). All seeds were obtained from the Active Germplasm Bank of Embrapa Genetic Resources and Biotechnology (Cenargen, Brasília, Brazil).

**Table 1:** Genotypes of *Arachis* used for cytogenetic and genotyping analysis, indicating the state, number of chromosomes, genomic formula and identification.

Genotypes	State	2n	Genomic formula	Identification
*A. hypogaea* “Xingu/Nambikwara” intermediate	Cultivated by indians	40	AABB	Of 115*
*A. hypogaea* subsp. *hypogaea* var. *hypogaea*	Cultivated by indians	40	AABB	Of 120*
*A. hypogaea* type “Xingu”	Cultivated by indians	40	AABB	Of 122
*A. hypogaea* type “Xingu”	Cultivated by indians	40	AABB	Of 126*
*A. hypogaea “Nambikwara” type*	Cultivated by indians	40	AABB	Of 128
*A. hypogaea* subsp. *hypogaea* var. *hypogaea*	Cultivated	40	AABB	Tifguard
*A. hypogaea* subsp. *hypogaea* var. *hypogaea*	Cultivated	40	AABB	Runner IAC 886
*A. hypogaea* subsp. *hypogaea* var. *hypogaea*	Cultivated	40	AABB	Tifrunner
*A. hypogaea*subsp. *hypogaea* var. *hypogaea*	Cultivated	40	AABB	IAC OL4
*A. hypogaea*subsp. *hypogaea* var. *hypogaea*	Cultivated	40	AABB	Tif 5-646-10
*A. hypogaea* subsp. *hypogaea* var. *hypogaea*	Cultivated	40	AABB	Tif 13-1014
*A. hypogaea*subsp. *hypogaea* var. *hypogaea*	Cultivated	40	AABB	TifGp-2
*A. hypogaea* subsp. *hypogaea* var. *hypogaea*	Cultivated	40	AABB	FloRun 107
*A. hypogaea* subsp. *hypogaea* var. *hypogaea*	Cultivated	40	AABB	FloRun 157
*A. hypogaea* subsp. *hypogaea* var. *hypogaea*	Cultivated	40	AABB	FloRun 331
*A. hypogaea* subsp. *hypogaea* var. *hypogaea*	Cultivated	40	AABB	Florida Fancy
*A. hypogaea* subsp. *hypogaea* var. *hypogaea*	Cultivated	40	AABB	Florida-EP 113
*A. hypogaea* subsp. *hypogaea* var. *hypogaea*	Cultivated	40	AABB	GA-06G
*A. hypogaea* subsp. *hypogaea* var. *hypogaea*	Cultivated	40	AABB	GA-09B
*A. hypogaea* subsp. *hypogaea* var. *hypogaea*	Cultivated	40	AABB	GA-12Y
*A. hypogaea* subsp. *hypogaea* var. *hypogaea*	Cultivated	40	AABB	TUFRunner 511
*A. hypogaea* subsp. *hypogaea* var. *hypogaea*	Cultivated	40	AABB	TUFRunner 297
*A. hypogaea* subsp. *hypogaea* var. *hypogaea*	Cultivated	40	AABB	TUFRunner 727
*A. hypogaea* subsp. *fastigiata* Waldron var*. fastigiata*	Cultivated	40	AABB	IAC Tatu-ST*
*A. hypogaea* subsp. *fastigiata* var*. fastigiata*	Cultivated	40	AABB	BR1
*A. hypogaea* subsp. *fastigiata* var*. vulgaris* C. Harz	Cultivated	40	AABB	Senegal 55-437
*A. hypogaea* subsp. *fastigiata* var*. vulgaris*	Cultivated	40	AABB	Fleur 11
*A. monticola* Krapov. & Rigoni	Wild	40	AABB	V 14165*
*A. monticola*	Wild	40	AABB	Sc 21768
*A. monticola*	Wild	40	AABB	Sc 21769
*A. monticola*	Wild	40	AABB	K 30062
*A. monticola*	Wild	40	AABB	K 30063
*A. monticola*	Wild	40	AABB	Ba 7264
*A. duranensis* Krapov. & W.C. Greg.	Wild	20	AA	V 14167*
*A. stenosperma* Krapov. & W.C. Greg.	Wild	20	AA	V 10309*
*A. ipaënsis* Krapov. & W.C. Greg.	Wild	20	BB	K 30076*
*A. magna* Krapov., W.C. Greg. & C.E. Simpson	Wild	20	BB	K 30097*

* Accessions cytogenetically analyzed.

### Metaphase chromosomes

Seeds were germinated for 5 days at 25 °C, then plantlets were transferred to pots with soil and maintained in an open plan greenhouse at Embrapa Genetic Resources and Biotechnology, Brasília, DF, Brazil. After four weeks, root tips (5-10 mm long) were isolated from five plants, for each genotype and treated with 2 mM 8-hydroxyquinoline for 3 h at room temperature (Fernández and Krapovickas, 1994). Samples were incubated in a fixative solution containing absolute ethanol: glacial acetic acid (3:1, v/v) for 12 h at 4 °C. Somatic chromosome spreads were prepared according to Schwarzacher and Heslop-Harrison (2000). Meristems were digested in 10 mM citrate buffer containing 2% cellulase (from *Trichoderma viridae*; Onozuka R-10 Serva) and 20% pectinase (from *Aspergillus niger*, Sigma) for 2 h at 37 °C. Chromosomes of each root were spread in a drop of acetic acid 45% on a slide and mounted with coverslip. Spread was obtained under pressure and the best slides were selected using the phase contrast in the AxiosKop microscope (Zeiss, Oberkochen, Germany). Those slides containing more than ten metaphases nicely spread and clean were chosen and the coverslips removed using the difference of temperature between slide and coverslip after submersion of the slide in liquid nitrogen. Slides were air-dried for 24h and kept at −20°C until use.

### Heterochromatic banding

#### *CMA*_*3*_ */ DAPI banding*

In order to localize GC and AT-rich heterochromatin, CMA_3_/DAPI banding was conducted in all genotypes following Schweizer and Ambros (1994). Chromosome spreads were treated with the fluorophore chromomycin A3 (CMA_3_, 0,5 mg/ml) for 1 h at room temperature, posteriorly with 4’, 6-diamino-2-phenylindole (DAPI, 2 μg/ml) for 30 minutes at room temperature. Slides were mounted with glycerol/McIlvaine buffer. Analysis were conducted in the epifluorescent Zeiss AxioPhot photomicroscope (Zeiss, Oberkochen, Germany). Images were captured using Zeiss AxioCam MRc digital camera (Zeiss Light Microscopy, Göttingen, Germany) and AxioVision Rel. 4.8 software and further processed using the Adobe Photoshop CS software.

#### Probes for in situ hybridization

In order to obtain the probes for GISH, genomic DNA (1 μg) was isolated from young leaflets of *A. duranensis*, *A. stenosperma*, *A. ipaënsis* and *A. magna* (Table 1), according to one CTAB protocol (Doyle and Doyle, 1987). DNA was purified, then labeled with either, digoxigenin-11-dUTP (Roche Diagnostics Deutschland GmbH) or Cy3-dUTP (Roche Diagnostics Deutschland GmbH) by Nick Translation kit (Roche Diagnostics Deutschland GmbH). No previous fragmentation of DNA sequences was necessary since this kit contains DNase. For probes to be used in fluorescent *in situ* hybridization (FISH), clones containing the sequences corresponding to the 5S ribosomal DNA of *Lotus japonicus* (Pedrosa *et al.*, 2002) and 18S-5.8S-25S of *Arabidopsis thaliana* (Wanzenböck *et al.*, 1997) were used. The rDNA was isolated with the Illustra plasmid Prep Midi Flow kit (GE Heltcare) and rDNA sequences were labeled by Nick translation, using the same kit described for genomic probes.

#### In situ hybridization

The *in situ* hybridization experiments were performed as described by Schwarzacher and Heslop-Harrison (2000). The hybridization steps and conditions were similar for GISH and FISH. Metaphase spreads were pre-treated with RNase A (10 mg/ml) for 2 h at 37 °C, followed by treatment with pepsin (10 mg/ml) for 15 min at 37 °C. Slides were incubated in a fixative solution containing 4% paraformaldehyde for 10 min at room temperature. Double GISH used simultaneously, both genomic probes, each one from a different different diploid species (approx. 50 ng of each probe/slide). FISH also used both ribosomal probes simultaneously, 5S and 45S rDNA. Hybridizations were performed for 12 h at 37 °C, followed by 73% stringent washes, using saline citrate buffer (SSC) 2X. Loci obtained by hybridization with the probe labeled with digoxigenin-11-dUTP were immunocytochemically detected, using the antibody anti-digoxigenin conjugated to fluorescein (Roche Diagnostics), while loci obtained with Cy3-dUTP probes were detected by direct observation in the epifluorescence microscope. Slides were counterstained with DAPI before observation in the Zeiss AxioPhot.

#### SNP genotyping and data analysis

SNP genotyping was performed to analyze the genetic relationships of 32 accessions including the Xingu accessions, representatives of the two subspecies of *A. hypogaea* and *A. monticola* (Table 1).

Genotyping was done using the *Axiom_Arachis2* 48K array (Korani *et al.*, 2019). Data were analyzed using Axiom Analysis Suite v.1.1.0.616 (Applied Biosystems, USA) and filtered by quality using the QC call rate > 90%. The genotyping information was filtered, allowing a minor allele frequency (MAF) > 0.05 and 20% missing calls. Markers showing inconsistent calls from duplicates of the same sample were discarded. Data output was visualized in Microsoft Excel. Genetic distances were obtained by 1-IBS (identity by state) in pairwise comparisons of the 32 accessions and an UPGMA tree was constructed using Tassel 5 (Bradbury *et al.*, 2007). The tree was plotted using FigTree software v.1.4.4.

Additionally, 448 *A. stenosperma*-specific SNPs were identified and compared to the genotypes of Xingu samples and two subspecies of *A. hypogaea*. Six accessions of *A. stenosperma* were included in this analysis: V 7762, V 10309, V 13796, V 13840, V 13844, and HLK 410.

## Results

**Figure 1:**
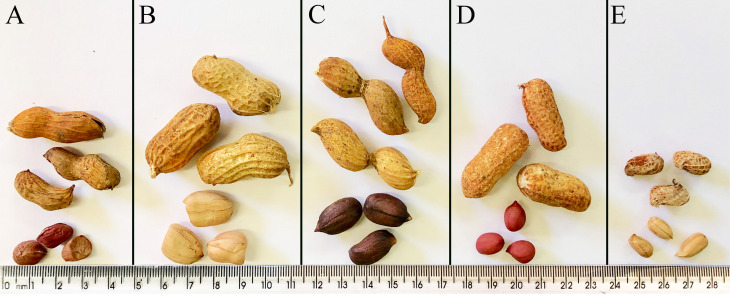
Pods and seeds of the *Arachis* genotypes cytogenetically analyzed showing differences in size, color and middle constriction. **A)** *A. hypogaea* Xingu/Nambikwara Of 115; **B)** *A. hypogaea* subsp. *hypogaea* var. *hypogaea* Of 120; **C)** *A. hypogaea* “Xingu” type Of 126; **D)** *A. hypogaea* subsp. *fastigiata* var. *fastigiata* ‘IAC Tatu-ST’ and **E)** *A. monticola* V 14165.

The choice of the Xingu accessions analyzed here considered first the larger morphological differences and genetic distances based on Freitas *et al.* (2007), followed by current germination and growth success. Differences in the morphology focused on shape, size, texture and color of the pods and seeds. Xingu/Nambikwara Of 115 (Figure 1A) is characterized by straight pods, prominent longitudinal and thick ridges and hard fruit shell, comprising some characteristics in between the other two types, the Nambikwara and Xingu. *Arachis hypogaea* subsp. *hypogaea* var. *hypogaea* Of 120 (Figure 1B) is considered the most primitive peanut cultivated by the Kayabi tribes, with some characteristics comparable to those of *A. monticola*, but much larger seeds. The third one, *A. hypogaea* Xingu type Of 126 (Figure 1C) has a thin and smooth fruit shell and an easily broken constriction in the middle of the pod. For comparison, the seeds of the Xingu accessions, cultivated peanut (*A. hypogaea* ‘IAC Tatu-ST’) (Figure 1D) and *A. monticola* (Figure 1E) are shown in Figure 1.

### Chromosome morphology

**Figure 2:**
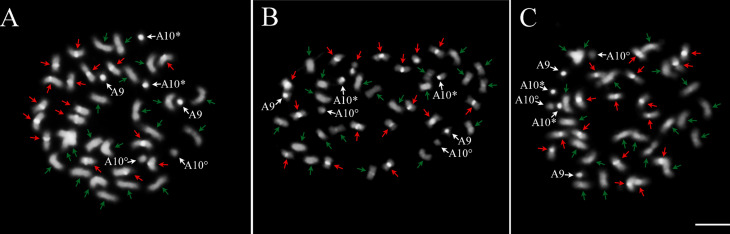
Metaphase chromosomes after DAPI counterstaining (bright white) in **A)** Xingu type of 126, representing similar results for the three Xingu accessions; **B**) *A. hypogaea* ‘IAC Tatu-ST’ and **C)** *A. monticola* V 14165. Ten pairs of chromosomes show DAPI^+^ bands on centromeric region of A subgenome (red arrows), while the other 10 pairs, corresponding to the chromosomes of the B subgenome, lack DAPI^+^ bands (green arrows). A9: small pair “A”. A10 with secondary constriction, short arm and proximal segment of the long arm (*) and satellite is (°). Bar: 5μm.

The chromosomes of the three accessions from Xingu Park were similar to each other, as shown by Of 126 as the representative of the three accessions (Figure 2A), and to cultivar ‘IAC Tatu-ST’ (Figure 2B) and *A. monticola* (Figure 2C), comprising 36 metacentric and four submetacentric chromosomes. One pair of chromosomes showed a secondary constriction, with a satellite segment, usually observed near to the proximal segment of the long arm of the chromosome, assigned as A10, based on the corresponding chromosomes 10 of the diploid progenitor *A. duranensis*, *A. hypogaea* and *A. monticola* (Seijo *et al.*, 2004). The small pair ‘A’, previously designated as chromosome 9 in *A. duranensis* was easily identified in all Xingu accessions due to the presence of the DAPI+ bands on the centromeres of these chromosomes, characteristic by the high condensation level of the heterochromatin.

### Distribution of heterochromatic banding

The Xingu accessions showed evident DAPI^+^ bands on the centromere region of ten pairs of chromosomes (A subgenome), corroborating the richness of repetitive A T in the DNA sequences of centromeres. These bands could not be detected on centromeres or other genome regions in the other ten pairs (B subgenome) (Figure 2A). DAPI^+^ banding pattern on the centromeres of chromosomes of the A subgenome was similar among the Xingu accessions, *A. hypogaea* ‘IAC Tatu-ST’ and *A. monticola* (Figures 2B and C).

**Figure 3:**
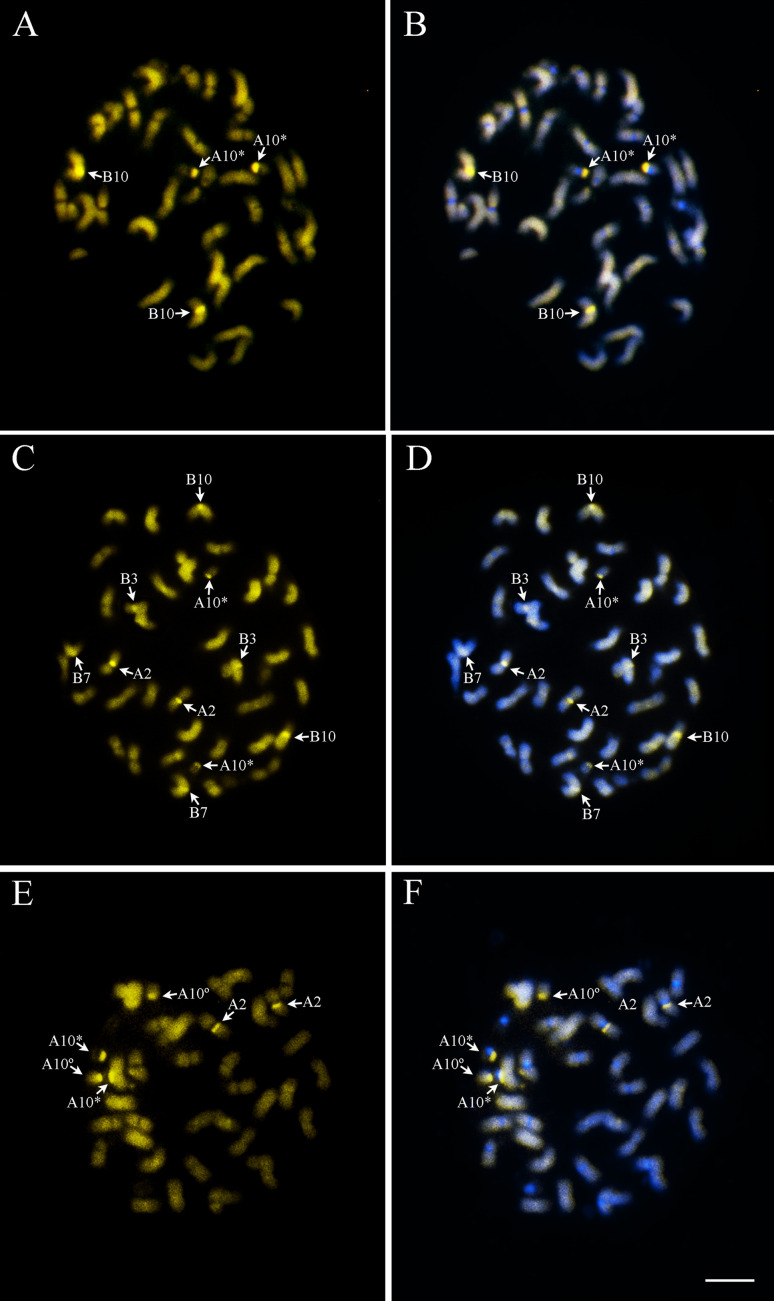
Chromosomes showing CMA_3_^+^ bands (arrows) on the proximal region of the chromosomes (A, C, E). Overlap of CMA_3_ (yellow) and DAPI (blue) (B, D, F). **A, B** Xingu type Of 126, representing the similar results of all three Xingu accessions; **C, D** *A. hypogaea* ‘IAC Tatu-ST’ and **E, F)** *A. monticola* V 14165. A10: short arm and proximal segments of the long arm (*) and satellite (°). Bar: 5μm.

The Xingu accessions had two pairs of chromosomes displaying CMA_3_^+^ bands, situated on the proximal regions of the chromosomes A10 and B10 (Figures 3A, B) and representing the sum of CMA_3_^+^ bands observed in *A. duranensis* and *A. ipaënsis* (Nascimento *et al.*, 2018). These bands differed from those present in the other tetraploids studied. In *A. hypogaea* 'IAC Tatu-ST’, besides the bands on chromosomes A10 and B10, there were bands most likely in A2, B3 and B7 (Figures 3C, D), while in *A. monticola*, CMA_3_^+^ bands were located only on chromosomes A2 and A10 (Figures 3E, F).

### Affinity of diploid genomes detected by GISH

**Figure 4:**
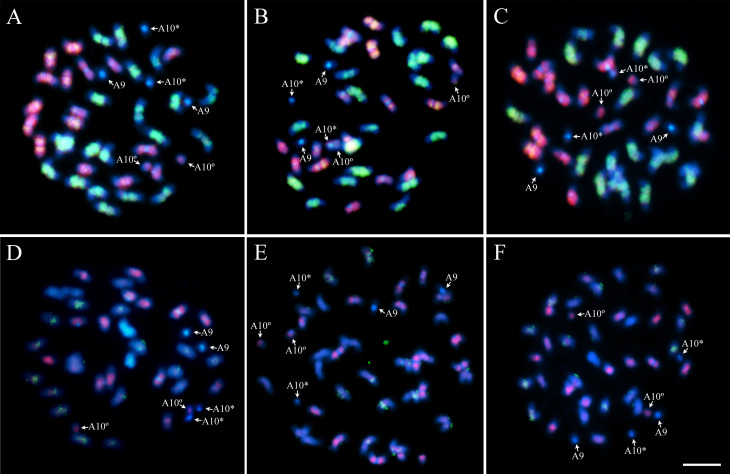
GISH using simultaneously the genomic probes from different diploid species in Xingu accessions. **A, D)** Xingu/Nambikwara Of 115; **B, E)** Of 120 and **C, F)** Xingu type Of 126. Hybridization with *A. duranensis* (red) and *A. ipaënsis* (green) probes (A, B, C), followed by DAPI counterstaining (blue), including the overlap of signals (greenish) of both probes. More discrete signals after hybridization with the probes from *A. stenosperma* (red) and signals almost absent in *A. magna* (green) (D, E, F). A10 with secondary constriction, short arm and proximal segment of the long arm (*) and satellite (°). Bar: 5μm.

Double GISH in chromosomes of the three Xingu accessions, using simultaneously the genomic probes from *A. duranensis* and *A. ipaënsis* (Figure S1) showed that each probe hybridized preferentially, with chromosomes of the corresponding subgenome, i.e., *A. duranensis* probe hybridized strongly and extensively with chromosomes of the A subgenome, whilst the *A. ipaënsis* probe hybridized clearly with chromosomes of the B subgenome (Figure 4A-C).

Signals after hybridization (GISH) were evident in all chromosomes, of both subgenomes, for the three Xingu accessions analyzed. Except for A9, short arm and proximal segment of the long arm of A10, that together with the centromeres of the chromosomes of the A subgenome and terminal regions of all chromosomes showed weak hybridization (Figures 4A-C). Furthermore, overlapping of hybridization signals, i.e., both probes hybridizing to the same DNA region was observed in most of the chromosomes, for both subgenomes, in all accessions.

The DNA from *A. stenosperma* (A genome) and *A. magna* (B genome) were also used as genomic probes for GISH (Figure S2) to investigate the possible contribution of these genomes, as donors for Xingu subgenomes. After hybridization with *A. stenosperma* probe, signals on Xingu accessions were mostly observed on chromosomes of the A subgenome, while few signals generated by hybridization with *A. magna* probe were dispersed on chromosomes of B subgenome, in all accessions (Figures 4D-F).

Most importantly, in the chromosomes of the Xingu accessions, hybridization with *A. duranensis* probe was stronger and uniform when compared to *A. stenosperma* probe. Similarly, hybridization with the *A. ipaënsis* probe was stronger and uniform than that with *A. magna* probe.

### Distribution of rDNA loci detected by FISH

**Figure 5:**
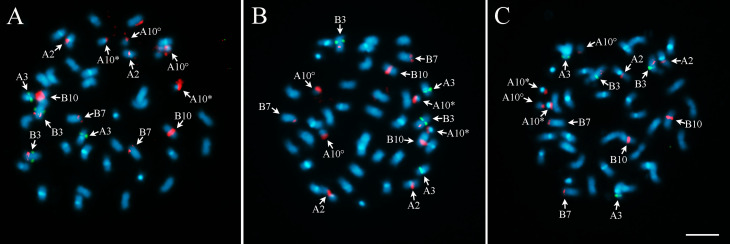
FISH using 5S (green) and 45S (red) rDNA probes, followed by DAPI counterstaining (blue). **A)** Xingu type Of 126; **B)** *A. hypogaea* ‘IAC Tatu-ST’ and **C)** *A. monticola* V 14165*.* Chromosome B3 shows 5S and 45S rDNA signals co-localized. A10 with secondary constriction, short arm and proximal segment of the long arm (*) and the satellite (°). Bar: 5μm.

The number, size and position of the 5S rDNA (green) loci on chromosomes of Xingu accessions were similar among them (Figure 5A), as well as in *A. hypogaea* ‘IAC Tatu-ST’ (Figure 5B) and *A. monticola* (Figure 5C). The loci were on the proximal region of chromosomes A3 and B3, along the short arms, as observed in the diploid species *A. duranensis*, *A. ipaënsis*, *A. stenosperma* and *A. magna*. This pattern corresponds to an additive character, with one locus from the species with A genome and the other from a B genome species, as already established for *A. hypogaea* and *A. monticola* (Seijo *et al.*, 2004; Robledo *et al.*, 2009; Robledo and Seijo, 2010).

Similarly, 45S rDNA loci (red) number is also an additive character in Xingu accessions (Figure 5A), *A. hypogaea* ‘IAC Tatu-ST’ (Figure 5B) and *A. monticola* (Figure 5C). Loci were located on the proximal regions of long arms of A2, A10 and B10, proximal regions of short arms of B3 and terminal region of short arms of B7. These loci result from the sum of the two loci from *A. duranensis* and three from *A. ipaënsis*, since the number, size and position are not compatible with the loci present in the other diploid species, *A. stenosperma* and *A. magna*.

Hybridization signals using the 45S rDNA probe can form, consistently, a distinguishable thread-like constriction linking the long segment and satellite region of A10 (Figure 5A). This aspect depicts the 45S rDNA being translated, what is characteristic of NORs (Nucleoli Organizing Region), which is frequently observed and available in the literature, including our previous work (Seijo *et al.*, 2004; Nascimento *et al.*, 2018). Co-localization of FISH signals after simultaneous use of 5S and 45S rDNA probes was observed on B3 of Xingu accessions (Figure 5A), as well as in *A. hypogaea* ‘IAC Tatu-ST’ (Figure 5B) and *A. monticola* (Figure 5C).

**Figure 6:**
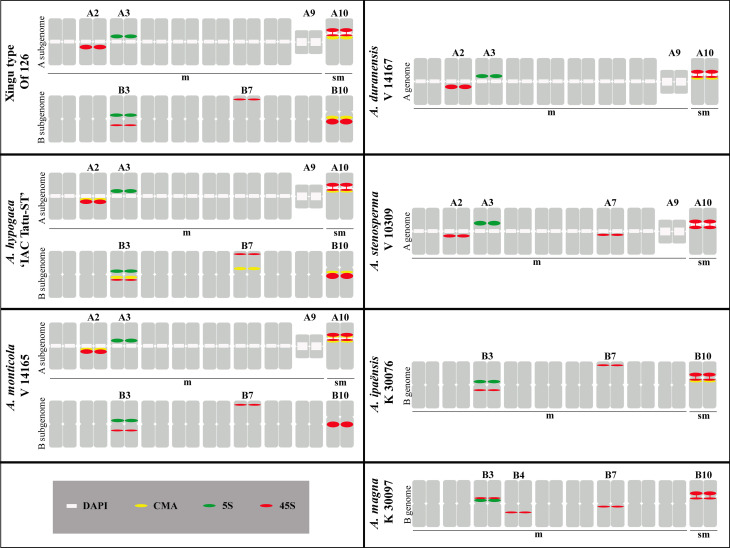
Schematic diagram of the *Arachis* karyotypes showing the morphology of chromosomes; position of centromeres (m: metacentric and sm: submetacentric); DAPI^+^ (white) and CMA_3_^+^ (yellow) bands and rDNA loci, 5S (green) and 45S (red) in chromosomes of Xingu type Of 126; *A. hypogaea* ‘IAC Tatu-ST’; *A. monticola* V 14165; *A. duranensis* V 14167; *A. stenosperma* V 10309; *A. ipaënsis* K 30076 and *A. magna* K 30097.

These results are summarized in Figure 6, including a karyotype of the Xingu type Of 126, representing the other two accessions studied, showing schematically chromosomes morphology, centromere position, DAPI^+^ and CMA_3_^+^ banding patterns and 5S and 45S rDNA loci distribution. Together, *A. hypogaea* subsp. *fastigiata* var. *fastigiata* ‘IAC Tatu-ST’, *A. monticola* (V 14165), *A. duranensis* (V 14167), *A. ipaënsis* (K 30076), *A. stenosperma* (V 10309) and *A. magna* (K 30097) karyotypes were included to enable an easy cytogenetic comparison.

### Genetic relationships based on SNP data

**Figure 7:**
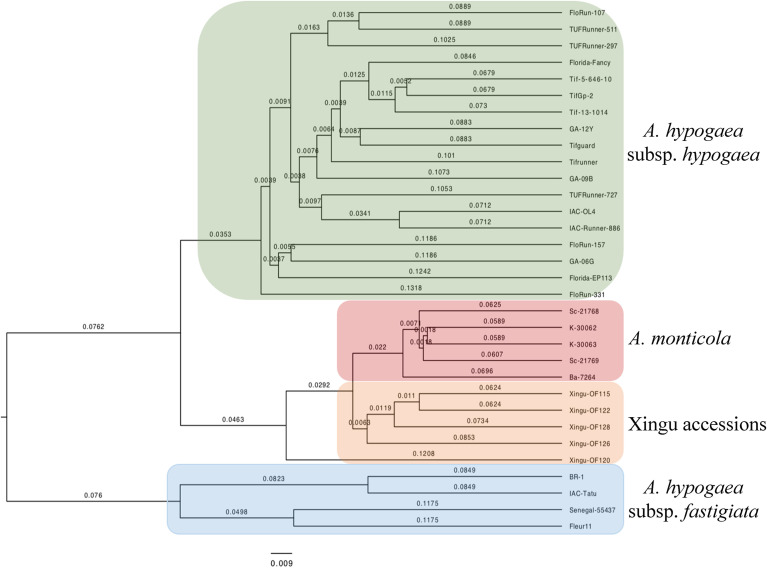
Dendrogram representing the genetic distance matrix based on SNP data, showing four taxonomic clusters including 22 *A. hypogaea*, five *A. monticola* and five Xingu Park accessions.

Five accessions from Xingu, 18 *A. hypogaea* subsp. *hypogaea*, four *A. hypogaea* subsp. *fastigiata*, and five *A. monticola* accessions were genotyped using the *Axiom_Arachis2*, an array composed of 47,837 SNPs. After filtering and exclusion of inconsistent calls, 7,883 SNPs were identified. Using this data, genetic distances in pairwise comparisons of the 32 accessions (Table 1) were estimated by the formula 1-IBS, with IBS defined as the probability that alleles drawn at random from two individuals at the same locus are the same (Figure S3). The resulting matrix was used to construct a dendrogram using UPGMA (Figure 6). The 32 accessions were grouped according to the four taxonomic groups, as expected: *A. hypogaea* subsp. *hypogaea*, *A. hypogaea* subsp. *fastigiata*, *A. monticola* and Xingu accessions (Figure 7). The Xingu accessions were closer to *A. monticola* than to the other *A. hypogaea* accessions of the two subspecies. When considering only *A. hypogaea*, the Xingu accessions were closer to subsp. *hypogaea*.

Affinity analysis of different Xingu accessions and accessions of *A. hypogaea* using *A. stenosperma* specific SNPs, showed no significant signal of *A. stenosperma* regions in any analyzed sample (Table S1). These data evidenced that *A. stenosperma* had not been involved in the origin of these Xingu accessions, as well as other cultivated peanut accessions.

## Discussion

High levels of genetic variation in Xingu Park accessions have previously been detected using microsatellite markers (Freitas *et al.*, 2007). In this study, 31 Xingu accessions were classified into three similarity groups, from which the Xingu accessions included in this study were selected. Despite of morphological differences and genetic variation evidenced by microsatellite markers, this cytogenetic study did not detect differences among the three Xingu accessions (Of 115, Of 120 and Of 126).

The similar DAPI^+^ heterochromatic banding pattern detected for the three Xingu accessions, *A. hypogaea* ‘IAC Tatu-ST’, and *A. monticola*, as well as as the previous description of for the induced allotetraploid IpaDur1 (Nascimento *et al.*, 2018), corresponded to the sum of the patterns present in *A. duranensis* and *A. ipaënsis*. This indicates the maintenance of the organization of the AT rich DNA sequences on the centromere region of the chromosomes of the A subgenome. Interestingly, DAPI^+^ centromeric bands are equally conserved in these allotetraploids, independent of spontaneous or induced origin and masculine or feminine role played by the species during the formation of allotetraploid.

On the other hand, a diversified pattern of CMA_3_^+^ bands could be herein observed. The distribution pattern of CMA_3_^+^ bands in the three Xingu accessions corresponds only to that found in the induced allotetraploid IpaDur1 (Nascimento *et al.*, 2018), which is the sum of the bands present in both the progenitor species, which differs from the patterns in *A. hypogaea* ‘IAC Tatu-ST’ and *A. monticola*. It is well known that CMA_3_^+^ bands are highly associated with rDNA loci, and NORs (Schweizer, 1976; Sato and Yoshioka, 1984; Guerra, 2000; Penãloza and Valls, 2005; Silva *et al.*, 2010), since these loci comprise DNA regions flanked by CG-rich heterochromatin (Salvadori *et al.*, 1995; Deiana *et al.*, 2000). The association between rDNA loci and CMA_3_ bands is entire only in *A. hypogaea*, ‘IAC Tatu-ST’, where the correspondence between number, position and intensity of CMA_3_^+^ bands and 45S rDNA loci is evident, suggesting the same type of heterochromatin in both (Galasso *et al.*, 1996; Cerbah *et al.*, 1998). However, not all GC-rich heterochromatin DNA regions behave equally to CMA_3_ (Schwarzacher and Schweizer, 1982; Kenton, 1991), as it is observed in other allotetraploids and diploids here studied, where the CMA_3_^+^ bands and 45S rDNA loci correspondence is only detected with a large 45S rDNA loci that corresponds to NORs.

Variation in CMA_3_^+^ bands distribution have been also reported for other plant species and their related genotypes (Guerra, 2000; Carvalho *et al.*, 2005; Cabral *et al.*, 2006), revealing differences in GC-rich heterochromatin (Schwezer, 1981). Additionally, it is acknowledged that most plant species have at least one pair of chromosomes containing a NOR, hence at least one pair of chromosomes displays the corresponding CMA_3_^+^ band (Morawetz, 1986; Röser, 1994; Guerra *et al.*, 2000). As like in *Arachis*, some species, such as *Hedera helix* (König *et al.*, 1987) and *Cicer arietinum* (Galasso *et al.*, 1996) have one or two pairs of CMA_3_^+^ bands at a very similar chromosome position to that of the NOR, although no secondary constriction can be observed, as it is the case of the chromosome B10 of these *Arachis* allotetraploids.

Therefore, the absence of direct and clear correspondence between CMA_3_^+^ bands and some of the 45S rDNA loci in the *Arachis* genomes may be the result of possible variations in DNA bases composition, affecting the fluorescence patterns obtained. For example, changes could be due to the type of DNA content along the chromosome and substitutions of DNA bases by analogs. Variations in the accessibility of the chromosomal DNA can result in differential protein distribution, associated with the chromosome and remodeling of chromatin fibers that could be here, influencing detection of CMA_3_ fluorescence (Schwezer, 1981). Yet, 45S rDNA loci could be so small that the corresponding CMA_3_ bands display bands with low intensity, not detectable (Zoldos *et al.*, 1999; Marcon *et al.*, 2003, 2005), as for example, *Citrus* (Carvalho *et al.*, 2005) and *Maxillaria* (Cabral *et al.*, 2006), where the correspondence between rDNA loci and CMA_3_^+^ bands is not complete.[Bibr B62]

There are numerous processes involved in chromatin remodeling, therefore rDNA sequences may exist in distinct conformation in allotetraploids mainly, since the doubling of genomes significantly affects gene expression, resulting for example, in induced gene silencing (Liu and Wendel, 2002, 2003). These changes are mostly determined by specific epigenetic codes, such as cytosine methylation and post translational changes in histones (Neves *et al.*, 2005) that affect the chromatin structure and interaction with fluorophores (Cabral *et al.*, 2006). Since expected 45S rDNA loci were detected in all genotypes after *in situ* hybridization, it is considered that heterochromatin is differently organized in these genotypes of *Arachis*, where depending the levels of condensation, the detection of CMA_3_^+^ bands can be favored or not (Schwezer, 1981).

GISH using probes of *A. duranensis* and *A. ipaënsis* on chromosomes of Xingu accessions corroborated the preferential hybridization of these diploid genomes to their corresponding subgenomes, as previously described for *A. hypogaea*, *A. monticola* and the induced allotetraploid, IpaDur1 (*A. duranensis* x *A. ipaënsis*)^4x^ (Seijo *et al.*, 2007; Nascimento *et al.*, 2018). The hybridization on A chromosomes of Xingu accessions with *A. duranensis* probe, together with the exclusive DAPI^+^ bands on centromeres of the chromosomes of the A subgenome allowed the differentiation of A and B subgenomes in these accessions. Additionally, the numerous repetitive elements between subgenomes was indicated by numerous overlapping signals after double GISH in Xingu chromosomes, consistent to what has been previously shown in other *Arachis* allotetraploids (Nascimento *et al.*, 2018).

The low levels of hybridization on A9, A10 and the terminal regions of chromosomes of the allotetraploids reflects a low repetitive DNA content, and, being gene rich (Bertioli *et al.*, 2016), are likely in an open chromatin conformation and and therefore, expected to show lower levels of fluorescence intensity in GISH assays (Seijo *et al.*, 2007; Nascimento *et al.*, 2018). Lastly, the centromeric regions of A subgenomes of *Arachis* seems to be evidenced only by DAPI, although centromeres are the most condensed regions of chromosomes that could be labeled by *in situ* hybridization. Therefore, the poor hybridization on the centromeric regions of A subgenome chromosomes may be related to different levels of accessibility of the probes to the homologous sequences (Seijo *et al.*, 2007). The accessibility also depends on the DNA sequence organization, determined by the families of tandemly repetitive DNA sequences present in the region. It is important to note that longer exposure to enzymatic treatment can improve the hybridization but results in a considerable loss of chromosome morphology.

Double GISH, using the genomic probes from *A. stenosperma* and *A. magna* showed lower hybridization levels than observed after hybridization with *A. duranensis* and *A. ipaënsis* probes, respectively. This is a key cytogenetic evidence that the latter species were also the genome donors of the Xingu accessions, and the contribution of *A. stenosperma* or *A. magna* to Xingu genomes is very unlikely. In fact, the center of origin of *A. stenosperma* is nearby the Xingu Indigenous Park and hybridization with its genomic probe shows evident signals, however this may indicate the occurrence of similarities between repetitive elements with another *Arachis* species with A genome, such as *A. duranensis* (Bertioli *et al.*, 2013, 2016; Moretzsohn *et al.*, 2013; Shirasawa *et al.*, 2013). In addition, five Xingu accessions were screened for SNPs present in seven diverse accessions of *A. stenosperma* and absent in two accessions of *A. duranensis*, *A. ipaënsis* and six non-Xingu *A. hypogaea* – no significant signal of these *A. stenosperma* SNPs was found in the Xingu accessions.

Besides, intron sequences from the A subgenome and their homeologs from the B subgenome were sequenced in seven accessions of *A. hypogaea*, representing both subspecies and the six varieties, plus an accession collected in Xingu Indigenous Park (Of 122), showing that the A and B subgenomes of all genotypes analyzed were grouped with *A. duranensis* and *A. ipaënsis*, respectively, and separately from all the other wild diploid species, including *A. stenosperma* (Moretzsohn *et al.*, 2013). It is also important to mention that the patterns resulting from recombination between ancestral A and B genomes are extremely similar in more than 200 genotypes of *A. hypogaea*, and other three Xingu accessions (Of 292, Of 299, and Of 303) (Bertioli *et al.*, 2019). Together, these studies studies corroborate the shared origin and similarity between Xingu accessions and representatives accessions of the two subspecies and six varieties of *A. hypogaea*.

In the three Xingu accessions, the number and localization of 5S rDNA loci were the same and corresponded to the sum of those found in *A. duranensis* and *A. ipaënsis*, as well as *A. stenosperma* and *A. magna*. Moreover, the distribution was the same as previously found for other *A. hypogaea* accessions, *A. monticola*, several *Arachis* diploid species, and IpaDur1 (Seijo *et al.*, 2004; Robledo and Seijo, 2010; Nascimento *et al.*, 2018), confirming the stability and heritance of 5S rDNA sequence in *Arachis* genome.

The 45S rDNA loci distribution in the three Xingu accessions, observed on A2, A10, B3, B7 and B10 chromosomes, was similar to other *A. hypogaea* accessions and *A. monticola* (Seijo *et al.*, 2004; Nascimento *et al.*, 2018), but different from IpaDur1, that lacks loci on B7 and B10. The loss of 45S rDNA loci in IpaDur1 might be related to the fact that it has *A. ipaënsis* as the female progenitor, the opposite direction to *A. hypogaea* and *A. monticola*. Despite this difference, the presence of the NOR on the chromosomes of the A subgenome in these *Arachis* allotetraploids corroborates the dominance of A10 of *A. duranensis* after allotetraploidization, as described for IpaDur1 and other spontaneous *Arachis* allotetraploids (Nascimento *et al.*, 2018).

The 45S rDNA loci in the three Xingu accessions were similar to the sum of those present in *A. duranensis* (A2, A10) and *A. ipaënsis* (B3, B7 and B10), distinctive from those in *A. stenosperma* (A2, A10 and A7) (Robledo *et al.*, 2009) and *A. magna* (B3, B7, B10 and B4) (Robledo and Seijo, 2010). Interestingly, the cytogenetic analysis of five Brazilian accessions of *A. magna* (V 13748, V 13765, V 14724, V 14727 and V 14750) (Custódio *et al.*, 2013) identified polymorphisms of the 45S rDNA loci among themselves, and these various patterns also differed from the K 30097. Most importantly is that all of the patterns observed in these accessions were different from that in the Xingu accessions and conclusively, it is assumed that none of these accessions may had participate in the genome origin of the Xingu accessions. On the other hand, is interesting to note that the accession V 14165 of *A. monticola*, despite being from Argentina, showed similar patterns of rDNA distribution and GISH affinity to those previously determined for another three accessions (Sn 2774, Sn 2775 and K 30062) (Seijo *et al.*, 2004). Additionally, the genotyping (SNPs) of others five accessions (Sc 21768, Sc 21769, K 30062, K 30063 and Ba 7264) confirmed the high similarity among them, suggesting that *A. monticola* accessions are genetically very close to each other, and may share similar characteristics, including the cytogenetic patterns, what allowed us the generalization of the cytogenetic conclusion based in our experiments with Argentinian.

Considering other *Arachis* species with A and B genomes previously studied and comparing with these Xingu accessions, it is possible to observe differences, such as the morphology of the chromosomes, the heterochromatin and SAT chromosomes (Robledo *et al.*, 2009; Robledo and Seijo, 2010), number, size and localization of rDNA loci and variation in the NORs (Seijo *et al.*, 2004; Robledo *et al.*, 2009; Robledo and Seijo, 2010). In addition, the low genomic affinity of these diploid species was observed by GISH when hybridized to chromosomes of *A. hypogaea* and *A. monticola* (Raina and Mukai, 1999; Seijo *et al.*, 2007), strengthening the hypothesis that *A. duranensis* and *A. ipaënsis* are the closest genomes and share similarities with the allotetraploids studied here.

Overall, the majority of the cytogenetic differences detected among the Xingu accessions, *A. hypogaea* ‘IAC Tatu-ST’ and *A. monticola* are related to the distribution of CMA_3_^+^ bands, more specifically in chromosomes B3, B7 and B10, suggesting that the B subgenome, inherited from *A. ipaënsis*, may be more affected by allotetraploidization than A subgenome. Differences in chromosomes B3 and B7 were also observed in the induced allotetraploid IpaDur1 (Nascimento *et al.*, 2018), where 45S rDNA loci lacked detection. Furthermore, chromosome B10 of this induced allotetraploid shows the indicative of recombination between A and B subgenomes, after double GISH. Finally, the SNP genotyping studies detected deletions and recombinations, such as tetrasomy and exchange of DNA blocks and alleles interspersed along the chromosomal segments, between the induced allotetraploid subgenomes (Bertioli *et al.*, 2019), a bias of DNA sequences of the B subgenome towards the A subgenome. These data strengthen the idea that the genomic instability may be affecting more the reorganization of chromosomes B.

For SNP genotyping, two additional accessions from Xingu Park were included: Of 122 also classified as Xingu type, and Of 128, morphologically distinguished as Nambikwara type, characterized by very large seeds, straight pods with prominent longitudinal ridges, and a thick, hard fruit shell (Freitas *et al.*, 2007). Despite being morphologically very different, four of the five accessions included grouped together, corroborating the results of the cytogenetic analyses. The exception was Of 120, which is considered the most antique among the peanuts cultivated by the Kayabi, having some characteristics comparable to those of *A. monticola*, but with much larger seeds. Of 120 showed to be closely related to *A. monticola* in a previous study (Freitas *et al.*, 2007). However, it is morphologically different from the other Xingu accessions and classified as *A. hypogaea* subsp. *hypogaea* var. *hypogaea*. Therefore, its clustering outside the group of Xingu and *A. monticola* was expected and seems to be more realistic. The number and genomic distribution of SNP markers used in the present study (47,837) was considerably higher than the 13 microsatellite loci used in the previous study of Freitas *et al.* (2007) and can explain this contradictory result.

The Xingu Park accessions were found to be more closely related to *A. monticola* than to *A. hypogaea* accessions from the two subspecies. Although classified as *A. hypogaea,* the Xingu Park accessions have morphological traits, especially in the pods, that exceeds the variation described for the species. *Arachis monticola* is considered the immediate tetraploid ancestor from which *A. hypogaea* has arisen upon domestication (Raina and Mukai, 1999; Grabiele *et al.*, 2012; Bertioli *et al.*, 2019). The group containing the Xingu and *A. monticola* accessions was more closely related to *A. hypogaea* subsp. *hypogaea* than to the group of *A. hypogaea* subsp. *fastigiata* accessions, as expected and corroborating the microsatellite-based analysis of Freitas *et al.* (2007). These results suggest that the accessions from Xingu Park have experienced a distinct process of evolution under artificial selection, as compared to the other *A. hypogaea* accessions, despite of the origin from the same diploid species. The taxonomic status of the Xingu Park accessions deserves further investigation.

Conclusively, the hypothesis that all varieties and subspecies of *A. hypogaea* originated from a single allotetraploid, or that they could had arisen from allotetraploid populations originating from the same two diploid species (Seijo *et al.*, 2007) is herein reinforced. Our results strongly suggest the same origin for the different accessions of Xingu and the two subspecies of *A. hypogaea*. The contribution of *A. duranensis* and *A. ipaënsis* genomes for the formation of the three Xingu Park accessions was confirmed, together with the identification of many cytogenetic resemblances among themselves and with the *fastigiata* subspecies of *A. hypogaea*, wild species and induced allotetraploids.

We conclude that the morphological variability existing in the three Xingu Park accessions is not due to the participation of different diploid species on their origin, but it is mostly result of the high morphological plasticity and selection done by the Brazilian indigenous people. To further investigate the taxonomic status of the Xingu Park accessions, additional collections of *Arachis* germplasm are necessary, followed by characterization and conservation of known and new genotypes.

## Supplementary material

The following online material is available for this article:

Figure S1 Double GISH showing similar labeling patterns in chromosomes of Xingu/Nambikwara of 115.

Figure S2 Double GISH showing similar labeling patterns in chromosomes of Xingu/Nambikwara Of 115.

Figure S3 Matrix representing the genetic distance among the *Arachis* accessions obtained by 1-IBS (identity by state) in pairwise comparisons.

Table S1 Genotyping of *Arachis* accessions using *A. stenosperma* specific SNPs.
