# Supplementary material for: Brazilian Kayabi Indian accessions of peanut, Arachis hypogaea (Fabales, Fabaceae): origin, diversity and evolution
Source: Genet Mol Biol. 2020 Nov 6;43(4):e20190418. doi: 10.1590/1678-4685-GMB-2019-0418 (PMC7644258; doi:10.1590/1678-4685-GMB-2019-0418)
Supplement: Supplementary file 2 [file 1415-4757-GMB-43-4-e20190418-suppl2.pdf]

Genetics and Molecular Biology

DOI: https://doi.org/ 10.1590/1678-4685-GMB-2019-0418

Supplementary material to "Brazilian Kayabi Indian accessions of peanut, *Arachis hypogaea* (Fabales, Fabaceae): origin, diversity and evolution"

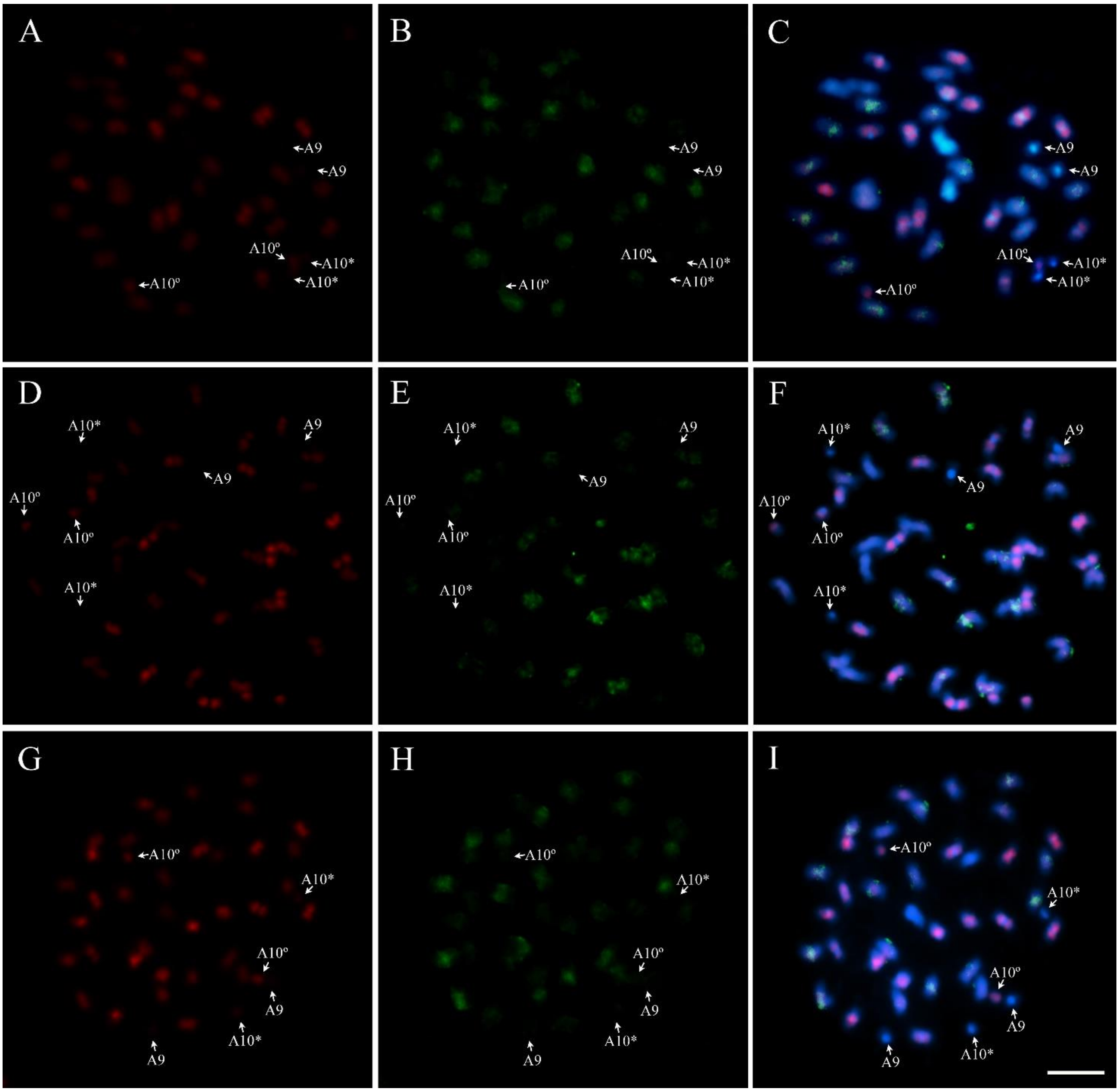

Figure S2: Double GISH showing similar labeling patterns in chromosomes of Xingu/Nambikwara Of 115 **(A/B/C)**; Of 120 **(D/E/F)** and Xingu type Of 126 **(G/H/I)**. Labelling in red after hybridization with *A. stenosperma* probe **(A, D, G)**, and in green, with *A. magna* **(B, E, H)**. Images with merged signals from both hybridizations and DAPI counterstaining (blue) **(C, F, I)**. A10 with the secondary constriction, the short arm and proximal segments of the long arm (*) and satellite (°). Bar: 5µm.
