# Supplementary material for: Brazilian Kayabi Indian accessions of peanut, Arachis hypogaea (Fabales, Fabaceae): origin, diversity and evolution
Source: Genet Mol Biol. 2020 Nov 6;43(4):e20190418. doi: 10.1590/1678-4685-GMB-2019-0418 (PMC7644258; doi:10.1590/1678-4685-GMB-2019-0418)
Supplement: Supplementary file 3 [file 1415-4757-GMB-43-4-e20190418-suppl3.pdf]

Genetics and Molecular Biology

DOI: https://doi.org/ 10.1590/1678-4685-GMB-2019-0418

Supplementary material to "Brazilian Kayabi Indian accessions of peanut, *Arachis hypogaea* (Fabales, Fabaceae): origin, diversity and evolution"

	FloRun 107	FloRun 157	FloRun 331	Florida Fancy	Florida-EP 113	TUFRunner 297	TUFRunner 511	TUFRunner 727	IAC OL4	GA-12Y	GA-09B	GA-06G	Tif-5-646-10	Tif-13-1014	Runner IAC 886	Tifguard
**FloRun 107**	0,00000	0,25177	0,24390	0,20809	0,27001	0,20562	0,17777	0,21766	0,26558	0,21558	0,22092	0,27451	0,20318	0,18614	0,25767	0,22784
**FloRun 157**	0,25177	0,00000	0,24398	0,23905	0,24027	0,23453	0,25875	0,26441	0,24915	0,25025	0,24286	0,23730	0,22097	0,22923	0,23831	0,24348
**FloRun 331**	0,24390	0,24398	0,00000	0,26016	0,28752	0,24182	0,25984	0,25365	0,27453	0,28700	0,26814	0,25454	0,25195	0,25805	0,26701	0,28286
**Florida Fancy**	0,20809	0,23905	0,26016	0,00000	0,25738	0,25380	0,23552	0,22141	0,21261	0,21466	0,20599	0,24054	0,17074	0,17164	0,20666	0,19983
**Florida-EP 113**	0,27001	0,24027	0,28752	0,25738	0,00000	0,25653	0,26550	0,26190	0,26479	0,26327	0,28216	0,25636	0,24558	0,24669	0,25671	0,26093
**TUFRunner 297**	0,20562	0,23453	0,24182	0,25380	0,25653	0,00000	0,20446	0,24787	0,28219	0,26191	0,27267	0,26304	0,22921	0,24471	0,27392	0,25905
**TUFRunner 511**	0,17777	0,25875	0,25984	0,23552	0,26550	0,20446	0,00000	0,21525	0,24455	0,24069	0,24516	0,29398	0,21290	0,21432	0,23585	0,24390
**TUFRunner 727**	0,21766	0,26441	0,25365	0,22141	0,26190	0,24787	0,21525	0,00000	0,21513	0,24680	0,21743	0,27445	0,23342	0,22814	0,20602	0,24746
**IAC OL4**	0,26558	0,24915	0,27453	0,21261	0,26479	0,28219	0,24455	0,21513	0,00000	0,25130	0,22469	0,26644	0,21316	0,22413	0,14235	0,22884
**GA-12Y**	0,21558	0,25025	0,28700	0,21466	0,26327	0,26191	0,24069	0,24680	0,25130	0,00000	0,18445	0,26921	0,20934	0,19580	0,24660	0,17663
**GA-09B**	0,22092	0,24286	0,26814	0,20599	0,28216	0,27267	0,24516	0,21743	0,22469	0,18445	0,00000	0,25614	0,22005	0,20473	0,22278	0,21966
**GA-06G**	0,27451	0,23730	0,25454	0,24054	0,25636	0,26304	0,29398	0,27445	0,26644	0,26921	0,25614	0,00000	0,24536	0,24441	0,26740	0,27322
**Tif-5-646-10**	0,20318	0,22097	0,25195	0,17074	0,24558	0,22921	0,21290	0,23342	0,21316	0,20934	0,22005	0,24536	0,00000	0,14742	0,20438	0,18087
**Tif-13-1014**	0,18614	0,22923	0,25805	0,17164	0,24669	0,24471	0,21432	0,22814	0,22413	0,19580	0,20473	0,24441	0,14742	0,00000	0,21603	0,17666
**Runner IAC 886**	0,25767	0,23831	0,26701	0,20666	0,25671	0,27392	0,23585	0,20602	0,14235	0,24660	0,22278	0,26740	0,20438	0,21603	0,00000	0,22036
**Tifguard**	0,22784	0,24348	0,28286	0,19983	0,26093	0,25905	0,24390	0,24746	0,22884	0,17663	0,21966	0,27322	0,18087	0,17666	0,22036	0,00000
**TifGp-2**	0,19608	0,23044	0,26097	0,16499	0,23900	0,23554	0,21956	0,22758	0,21854	0,20299	0,21514	0,24486	0,13579	0,14477	0,20935	0,17271
**Tifrunner**	0,23573	0,26352	0,28535	0,21774	0,25898	0,28197	0,25565	0,27849	0,26435	0,20962	0,25285	0,28420	0,20400	0,19152	0,25516	0,19739
**Sc 21768**	0,33821	0,33685	0,28716	0,35250	0,33548	0,33227	0,34414	0,33932	0,36551	0,34182	0,34372	0,34245	0,35592	0,35226	0,36814	0,34340
**K 30062**	0,33786	0,34057	0,28643	0,35346	0,33435	0,33113	0,34338	0,33861	0,36838	0,34424	0,34580	0,34510	0,35689	0,35328	0,37001	0,34437
**K 30063**	0,33784	0,33846	0,28631	0,35379	0,33454	0,33024	0,34339	0,33828	0,36863	0,34328	0,34603	0,34333	0,35733	0,35401	0,37025	0,34449
**Ba 7264**	0,35073	0,35124	0,29881	0,36604	0,34620	0,34363	0,35585	0,35068	0,38109	0,35430	0,35776	0,35737	0,36936	0,36578	0,38229	0,35583
**Sc 21769**	0,33568	0,33522	0,28565	0,35213	0,33412	0,32880	0,34129	0,33682	0,36496	0,33971	0,34171	0,33917	0,35385	0,35034	0,36658	0,33976
**Xingu Of 115**	0,33455	0,32524	0,27798	0,33785	0,32304	0,31574	0,34590	0,33451	0,36411	0,33640	0,33834	0,32532	0,34744	0,34540	0,36434	0,33534
**Xingu Of 120**	0,28836	0,31113	0,27387	0,31311	0,29025	0,29019	0,31187	0,33062	0,37822	0,29152	0,33319	0,33035	0,30495	0,29363	0,37804	0,29258
**Xingu Of 122**	0,33460	0,32586	0,27899	0,33833	0,32370	0,31627	0,34647	0,33518	0,36448	0,33533	0,33893	0,32543	0,34803	0,34606	0,36447	0,33493
**Xingu Of 126**	0,30321	0,28618	0,25290	0,29750	0,29808	0,28884	0,30662	0,29415	0,30429	0,30562	0,30315	0,29592	0,30477	0,30498	0,30385	0,30202
**Xingu OF128**	0,33644	0,32680	0,28021	0,34838	0,33029	0,31991	0,34252	0,32930	0,36501	0,34479	0,34149	0,33309	0,35576	0,35277	0,36386	0,34556
**BR1**	0,54499	0,55479	0,51186	0,56079	0,54522	0,54418	0,54010	0,54722	0,55136	0,53693	0,53617	0,54956	0,56538	0,56119	0,55713	0,54193
**Senegal 55-437**	0,52627	0,53259	0,51930	0,51555	0,51112	0,54257	0,50847	0,51766	0,48700	0,49025	0,49007	0,52121	0,52316	0,51658	0,49149	0,49634
**Fleur 11**	0,52629	0,54875	0,51935	0,53204	0,53299	0,54249	0,50699	0,51197	0,49489	0,50512	0,50281	0,53630	0,54216	0,53440	0,50015	0,51494
**IAC Tatu-ST**	0,52705	0,53282	0,49061	0,54081	0,52247	0,52722	0,52268	0,52831	0,53499	0,52104	0,52100	0,52970	0,54403	0,53776	0,53820	0,52462

	TifGp-2	Tifrunner	Sc 21768	K 30062	K 30063	Ba 7264	Sc 21769	Xingu Of 115	Xingu Of 120	Xingu Of 122	Xingu Of 126	Xingu Of 128	BR-1	Senegal 55-437	Fleur 11	IAC Tatu-ST
**FloRun 107**	0,19608	0,23573	0,33821	0,33786	0,33784	0,35073	0,33568	0,33455	0,28836	0,33460	0,30321	0,33644	0,54499	0,52627	0,52629	0,52705
**FloRun 157**	0,23044	0,26352	0,33685	0,34057	0,33846	0,35124	0,33522	0,32524	0,31113	0,32586	0,28618	0,32680	0,55479	0,53259	0,54875	0,53282
**FloRun 331**	0,26097	0,28535	0,28716	0,28643	0,28631	0,29881	0,28565	0,27798	0,27387	0,27899	0,25290	0,28021	0,51186	0,51930	0,51935	0,49061
**Florida Fancy**	0,16499	0,21774	0,35250	0,35346	0,35379	0,36604	0,35213	0,33785	0,31311	0,33833	0,29750	0,34838	0,56079	0,51555	0,53204	0,54081
**Florida-EP 113**	0,23900	0,25898	0,33548	0,33435	0,33454	0,34620	0,33412	0,32304	0,29025	0,32370	0,29808	0,33029	0,54522	0,51112	0,53299	0,52247
**TUFRunner 297**	0,23554	0,28197	0,33227	0,33113	0,33024	0,34363	0,32880	0,31574	0,29019	0,31627	0,28884	0,31991	0,54418	0,54257	0,54249	0,52722
**TUFRunner 511**	0,21956	0,25565	0,34414	0,34338	0,34339	0,35585	0,34129	0,34590	0,31187	0,34647	0,30662	0,34252	0,54010	0,50847	0,50699	0,52268
**TUFRunner 727**	0,22758	0,27849	0,33932	0,33861	0,33828	0,35068	0,33682	0,33451	0,33062	0,33518	0,29415	0,32930	0,54722	0,51766	0,51197	0,52831
**IAC OL4**	0,21854	0,26435	0,36551	0,36838	0,36863	0,38109	0,36496	0,36411	0,37822	0,36448	0,30429	0,36501	0,55136	0,48700	0,49489	0,53499
**GA-12Y**	0,20299	0,20962	0,34182	0,34424	0,34328	0,35430	0,33971	0,33640	0,29152	0,33533	0,30562	0,34479	0,53693	0,49025	0,50512	0,52104
**GA-09B**	0,21514	0,25285	0,34372	0,34580	0,34603	0,35776	0,34171	0,33834	0,33319	0,33893	0,30315	0,34149	0,53617	0,49007	0,50281	0,52100
**GA-06G**	0,24486	0,28420	0,34245	0,34510	0,34333	0,35737	0,33917	0,32532	0,33035	0,32543	0,29592	0,33309	0,54956	0,52121	0,53630	0,52970
**Tif-5-646-10**	0,13579	0,20400	0,35592	0,35689	0,35733	0,36936	0,35385	0,34744	0,30495	0,34803	0,30477	0,35576	0,56538	0,52316	0,54216	0,54403
**Tif-13-1014**	0,14477	0,19152	0,35226	0,35328	0,35401	0,36578	0,35034	0,34540	0,29363	0,34606	0,30498	0,35277	0,56119	0,51658	0,53440	0,53776
**Runner IAC 886**	0,20935	0,25516	0,36814	0,37001	0,37025	0,38229	0,36658	0,36434	0,37804	0,36447	0,30385	0,36386	0,55713	0,49149	0,50015	0,53820
**Tifguard**	0,17271	0,19739	0,34340	0,34437	0,34449	0,35583	0,33976	0,33534	0,29258	0,33493	0,30202	0,34556	0,54193	0,49634	0,51494	0,52462
**TifGp-2**	0,00000	0,19145	0,35170	0,35248	0,35283	0,36436	0,34968	0,34252	0,29417	0,34242	0,30270	0,35018	0,56311	0,51941	0,53791	0,54002
**Tifrunner**	0,19145	0,00000	0,34976	0,35035	0,35024	0,33535	0,34750	0,32803	0,24085	0,32748	0,29434	0,33863	0,54568	0,50409	0,52240	0,52140
**Sc 21768**	0,35170	0,34976	0,00000	0,12461	0,12401	0,14036	0,12621	0,18231	0,25394	0,18322	0,20560	0,17408	0,55136	0,48700	0,49489	0,53499
**K 30062**	0,35248	0,35035	0,12461	0,00000	0,11782	0,13673	0,12086	0,17856	0,25084	0,18019	0,20402	0,16972	0,53693	0,49025	0,50512	0,52104
**K 30063**	0,35283	0,35024	0,12401	0,11782	0,00000	0,13877	0,12181	0,17776	0,25000	0,17952	0,20245	0,16908	0,53617	0,49007	0,50281	0,52100
**Ba 7264**	0,36436	0,33535	0,14036	0,13673	0,13877	0,00000	0,14099	0,17765	0,23614	0,17905	0,20289	0,16883	0,54956	0,52121	0,53630	0,52970
**Sc 21769**	0,34968	0,34750	0,12621	0,12086	0,12181	0,14099	0,00000	0,17848	0,25291	0,17913	0,20207	0,16883	0,56538	0,52316	0,54216	0,54403
**Xingu Of 115**	0,34252	0,32803	0,18231	0,17856	0,17776	0,17765	0,17848	0,00000	0,22882	0,12486	0,16986	0,14667	0,56119	0,51658	0,53440	0,53776
**Xingu Of 120**	0,29417	0,24085	0,25394	0,25084	0,25000	0,23614	0,25291	0,22882	0,00000	0,22914	0,23752	0,23495	0,55713	0,49149	0,50015	0,53820
**Xingu Of 122**	0,34242	0,32748	0,18322	0,18019	0,17952	0,17905	0,17913	0,12486	0,22914	0,00000	0,17023	0,14698	0,54193	0,49634	0,51494	0,52462
**Xingu Of 126**	0,30270	0,29434	0,20560	0,20402	0,20245	0,20289	0,20207	0,16986	0,23752	0,17023	0,00000	0,17179	0,56311	0,51941	0,53791	0,54002
**Xingu OF128**	0,35018	0,33863	0,17408	0,16972	0,16908	0,16883	0,16883	0,14667	0,23495	0,14698	0,17179	0,00000	0,54568	0,50409	0,52240	0,52140
**BR1**	0,56311	0,54568	0,37973	0,37527	0,37545	0,37378	0,37766	0,41133	0,48612	0,41231	0,43619	0,39787	0,00000	0,32736	0,32411	0,16987
**Senegal 55-437**	0,51941	0,50409	0,40955	0,40607	0,40662	0,40596	0,40935	0,43769	0,51766	0,43933	0,44918	0,43460	0,32736	0,00000	0,23496	0,34572
**Fleur 11**	0,53791	0,52240	0,41533	0,41092	0,41295	0,41174	0,41388	0,44487	0,52156	0,44520	0,45703	0,43443	0,32411	0,23496	0,00000	0,34078
**IAC Tatu-ST**	0,54002	0,52140	0,35535	0,35336	0,35270	0,34991	0,35390	0,38687	0,46130	0,38777	0,41215	0,37493	0,16987	0,34572	0,34078	0,00000

Figure S3: Matrix representing the genetic distance among the *Arachis* accessions obtained by 1-IBS (identity by state) in pairwise comparisons.
