# Supplementary material for: Brazilian Kayabi Indian accessions of peanut, Arachis hypogaea (Fabales, Fabaceae): origin, diversity and evolution
Source: Genet Mol Biol. 2020 Nov 6;43(4):e20190418. doi: 10.1590/1678-4685-GMB-2019-0418 (PMC7644258; doi:10.1590/1678-4685-GMB-2019-0418)
Supplement: Supplementary file 4 [file 1415-4757-GMB-43-4-e20190418-suppl4.pdf]

Genetics and Molecular Biology

DOI: https://doi.org/ 10.1590/1678-4685-GMB-2019-0418

Supplementary material to "Brazilian Kayabi Indian accessions of peanut, *Arachis hypogaea* (Fabales, Fabaceae): origin, diversity and evolution"

Table S1: Genotyping of *Arachis* accessions using *A. stenosperma* specific SNPs.

SNP ID	Chromosome ID	Start	*A. stenosperma* V 13840	*A. stenosperma* V 13844	*A. stenosperma* V 13844	*A. stenosperma* HLK-410	*A. stenosperma* V 7762	*A. stenosperma* V 10309	*A. stenosperma* V 10309	*A. stenosperma* V 13796	Xingu Of 115	Xingu Of 115	Xingu Of 115	Xingu Of 120	Xingu Of 120	Xingu Of 120	Xingu Of 122	Xingu Of 122	Xingu Of 126	Xingu Of 126	Xingu Of 126	Xingu Of 128	Xingu Of 128	Xingu Of 128	Tif-5-646-10	TifGp-2	Tif-13-1014	Tifguard	Tifguard	Tifrunner	Tifrunner	IAC-OL4	IAC-Runner-886	IAC-Runner-886	IAC Tatu-ST	IAC Tatu-ST
AX-147207653	Aradu.A01	328554	2sten	2sten	2sten	2sten	2sten	2sten	2sten	2sten	-	-	-	-	-	-	-	-	-	-	-	-	-	-	-	-	-	-	-	-	-	-	-	-	-	-
AX-176791317	Aradu.A01	510486	2sten	2sten	2sten	2sten	2sten	2sten	2sten	2sten	-	-	-	-	-	-	-	-	-	-	-	-	-	-	-	-	-	-	-	-	-	-	-	-	-	-
AX-147265635	Aradu.A01	595605	2sten	2sten	2sten	2sten	2sten	2sten	2sten	2sten	-	-	-	-	-	-	-	-	-	-	-	-	-	-	-	-	-	-	-	-	-	-	-	-	-	-
AX-147207739	Aradu.A01	738136	2sten	2sten	2sten	2sten	2sten	2sten	2sten	2sten	-	-	-	-	-	-	-	-	-	-	-	-	-	-	-	-	-	-	-	-	-	-	-	-	-	-
AX-147207741	Aradu.A01	748422	2sten	2sten	2sten	2sten	2sten	2sten	2sten	2sten	-	-	-	-	-	-	-	-	-	-	-	-	-	-	-	-	-	-	-	-	-	-	-	-	-	-
AX-147207744	Aradu.A01	773072	2sten	2sten	2sten	2sten	2sten	2sten	2sten	2sten	-	-	-	-	-	-	-	-	-	-	-	-	-	-	-	-	-	-	-	-	-	-	-	-	-	-
AX-147207750	Aradu.A01	785311	2sten	2sten	2sten	2sten	2sten	2sten	2sten	2sten	-	-	-	-	-	-	-	-	-	-	-	-	-	-	-	-	-	-	-	-	-	-	-	-	-	-
AX-147207751	Aradu.A01	795675	2sten	2sten	2sten	2sten	2sten	2sten	2sten	2sten	-	-	-	-	-	-	-	-	-	-	-	-	-	-	-	-	-	-	-	-	-	-	-	-	-	-

SNP ID	Chromosome ID	Start	*A. stenosperma* V 13840	*A. stenosperma* V 13844	*A. stenosperma* V 13844	*A. stenosperma* HLK-410	*A. stenosperma* V 7762	*A. stenosperma* V 10309	*A. stenosperma* V 10309	*A. stenosperma* V 13796	Xingu Of 115	Xingu Of 115	Xingu Of 115	Xingu Of 120	Xingu Of 120	Xingu Of 120	Xingu Of 122	Xingu Of 122	Xingu Of 126	Xingu Of 126	Xingu Of 126	Xingu Of 128	Xingu Of 128	Xingu Of 128	Tif-5-646-10	TifGp-2	Tif-13-1014	Tifguard	Tifguard	Tifrunner	Tifrunner	IAC-OL4	IAC-Runner-886	IAC-Runner-886	IAC Tatu-ST	IAC Tatu-ST
AX-147207796	Aradu.A01	1043380	2sten	2sten	2sten	2sten	2sten	2sten	2sten	2sten	-	-	-	-	-	-	-	-	-	-	-	-	-	-	-	-	-	-	-	-	-	-	-	-	-	-
AX-147207810	Aradu.A01	1117356	2sten	2sten	2sten	2sten	2sten	2sten	2sten	2sten	-	-	-	-	-	-	-	-	-	-	-	-	-	-	-	-	-	-	-	-	-	-	-	-	-	-
AX-147207811	Aradu.A01	1117514	2sten	2sten	2sten	2sten	2sten	2sten	2sten	2sten	-	-	-	-	-	-	-	-	-	-	-	-	-	-	-	-	-	-	-	-	-	-	-	-	-	-
AX-147265656	Aradu.A01	1185029	2sten	2sten	2sten	2sten	2sten	2sten	2sten	2sten	-	-	-	-	-	-	-	-	-	-	-	-	-	-	-	-	-	-	-	-	-	-	-	-	-	-
AX-147207859	Aradu.A01	1397162	2sten	2sten	2sten	2sten	2sten	2sten	2sten	2sten	-	-	-	-	-	-	-	-	-	-	-	-	-	-	-	-	-	-	-	-	-	-	-	-	-	-
AX-176791373	Aradu.A01	1399006	2sten	2sten	2sten	2sten	2sten	2sten	2sten	2sten	-	-	-	-	-	-	-	-	-	-	-	-	-	-	-	-	-	-	-	-	-	-	-	-	-	-
AX-147207867	Aradu.A01	1427146	2sten	2sten	2sten	2sten	2sten	2sten	2sten	2sten	-	-	-	-	-	-	-	-	-	-	-	-	-	-	-	-	-	-	-	-	-	-	-	-	-	-
AX-147207931	Aradu.A01	1651448	2sten	2sten	2sten	2sten	2sten	2sten	2sten	2sten	-	-	-	-	-	-	-	-	-	-	-	-	-	-	-	-	-	-	-	-	-	-	-	-	-	-
AX-147265676	Aradu.A01	1665661	2sten	2sten	2sten	2sten	2sten	2sten	2sten	2sten	-	-	-	-	-	-	-	-	-	-	-	-	-	-	-	-	-	-	-	-	-	-	-	-	-	-
AX-147207949	Aradu.A01	1724573	2sten	2sten	2sten	2sten	2sten	2sten	2sten	2sten	-	-	-	-	-	-	-	-	-	-	-	-	-	-	-	-	-	-	-	-	-	-	-	-	-	-
AX-147207952	Aradu.A01	1734177	2sten	2sten	2sten	2sten	2sten	2sten	2sten	2sten	-	-	-	-	-	-	-	-	-	-	-	-	-	-	-	-	-	-	-	-	-	-	-	-	-	-
AX-147207960	Aradu.A01	1776991	2sten	2sten	2sten	2sten	2sten	2sten	2sten	2sten	-	-	-	-	-	-	-	-	-	-	-	-	-	-	-	-	-	-	-	-	-	-	-	-	-	-
AX-147207965	Aradu.A01	1783393	2sten	2sten	2sten	2sten	2sten	2sten	2sten	2sten	-	-	-	-	-	-	-	-	-	-	-	-	-	-	-	-	-	-	-	-	-	-	-	-	-	-

SNP ID	Chromosome ID	Start	*A. stenosperma* V 13840	*A. stenosperma* V 13844	*A. stenosperma* V 13844	*A. stenosperma* HLK-410	*A. stenosperma* V 7762	*A. stenosperma* V 10309	*A. stenosperma* V 10309	*A. stenosperma* V 13796	Xingu Of 115	Xingu Of 115	Xingu Of 115	Xingu Of 120	Xingu Of 120	Xingu Of 120	Xingu Of 122	Xingu Of 122	Xingu Of 126	Xingu Of 126	Xingu Of 126	Xingu Of 128	Xingu Of 128	Xingu Of 128	Tif-5-646-10	TifGp-2	Tif-13-1014	Tifguard	Tifguard	Tifrunner	Tifrunner	IAC-OL4	IAC-Runner-886	IAC-Runner-886	IAC Tatu-ST	IAC Tatu-ST
AX-147207971	Aradu.A01	1788102	2sten	2sten	2sten	2sten	2sten	2sten	2sten	2sten	-	-	-	-	-	-	-	-	-	-	-	-	-	-	-	-	-	-	-	-	-	-	-	-	-	-
AX-147207989	Aradu.A01	1845093	2sten	2sten	2sten	2sten	2sten	2sten	2sten	2sten	-	-	-	-	-	-	-	-	-	-	-	-	-	-	-	-	-	-	-	-	-	-	-	-	-	-
AX-147208012	Aradu.A01	1916817	2sten	2sten	2sten	2sten	2sten	2sten	2sten	2sten	-	-	-	-	-	-	-	-	-	-	-	-	-	-	-	-	-	-	-	-	-	-	-	-	-	-
AX-147208016	Aradu.A01	1950958	2sten	2sten	2sten	2sten	2sten	2sten	2sten	2sten	-	-	-	-	-	-	-	-	-	-	-	-	-	-	-	-	-	-	-	-	-	-	-	-	-	-
AX-147208027	Aradu.A01	1979294	2sten	2sten	2sten	2sten	2sten	2sten	2sten	2sten	-	-	-	-	-	-	-	-	-	-	-	-	-	-	-	-	-	-	-	-	-	-	-	-	-	-
AX-147208038	Aradu.A01	1984064	2sten	2sten	2sten	2sten	2sten	2sten	2sten	2sten	-	-	-	-	-	-	-	-	-	-	-	-	-	-	-	-	-	-	-	-	-	-	-	-	-	-
AX-147208052	Aradu.A01	2010237	2sten	2sten	2sten	2sten	2sten	2sten	2sten	2sten	-	-	-	-	-	-	-	-	-	-	-	-	-	-	-	-	-	-	-	-	-	-	-	-	-	-
AX-147208069	Aradu.A01	2110672	2sten	2sten	2sten	2sten	2sten	2sten	2sten	2sten	-	-	-	-	-	-	-	-	-	-	-	-	-	-	-	-	-	-	-	-	-	-	-	-	-	-
AX-147208100	Aradu.A01	2272798	2sten	2sten	2sten	2sten	2sten	2sten	2sten	2sten	-	-	-	-	-	-	-	-	-	-	-	-	-	-	-	-	-	-	-	-	-	-	-	-	-	-
AX-147208105	Aradu.A01	2303964	2sten	2sten	2sten	2sten	2sten	2sten	2sten	2sten	-	-	-	-	-	-	-	-	-	-	-	-	-	-	-	-	-	-	-	-	-	-	-	-	-	-
AX-147208106	Aradu.A01	2304231	2sten	2sten	2sten	2sten	2sten	2sten	2sten	2sten	-	-	-	-	-	-	-	-	-	-	-	-	-	-	-	-	-	-	-	-	-	-	-	-	-	-
AX-147208130	Aradu.A01	2434115	2sten	2sten	2sten	2sten	2sten	2sten	2sten	2sten	-	-	-	-	-	-	-	-	-	-	-	-	-	-	-	-	-	-	-	-	-	-	-	-	-	-
AX-147208131	Aradu.A01	2439478	2sten	2sten	2sten	2sten	2sten	2sten	2sten	2sten	-	-	-	-	-	-	-	-	-	-	-	-	-	-	-	-	-	-	-	-	-	-	-	-	-	-

SNP ID	Chromosome ID	Start	*A. stenosperma* V 13840	*A. stenosperma* V 13844	*A. stenosperma* V 13844	*A. stenosperma* HLK-410	*A. stenosperma* V 7762	*A. stenosperma* V 10309	*A. stenosperma* V 10309	*A. stenosperma* V 13796	Xingu Of 115	Xingu Of 115	Xingu Of 115	Xingu Of 120	Xingu Of 120	Xingu Of 120	Xingu Of 122	Xingu Of 122	Xingu Of 126	Xingu Of 126	Xingu Of 126	Xingu Of 128	Xingu Of 128	Xingu Of 128	Tif-5-646-10	TifGp-2	Tif-13-1014	Tifguard	Tifguard	Tifrunner	Tifrunner	IAC-OL4	IAC-Runner-886	IAC-Runner-886	IAC Tatu-ST	IAC Tatu-ST
AX-147208153	Aradu.A01	2784761	2sten	2sten	2sten	2sten	2sten	2sten	2sten	2sten	-	-	-	-	-	-	-	-	-	-	-	-	-	-	-	-	-	-	-	-	-	-	-	-	-	-
AX-147208184	Aradu.A01	2897820	2sten	2sten	2sten	2sten	2sten	2sten	2sten	2sten	-	-	-	-	-	-	-	-	-	-	NN	-	-	-	-	-	-	-	-	-	-	-	-	-	-	-
AX-147265734	Aradu.A01	3120153	2sten	2sten	2sten	2sten	2sten	2sten	2sten	2sten	-	-	-	-	-	-	-	-	-	-	-	-	-	-	-	-	-	-	-	-	-	-	-	-	-	-
AX-147208245	Aradu.A01	3120399	2sten	2sten	2sten	2sten	2sten	2sten	2sten	2sten	-	-	-	-	-	-	-	-	-	-	-	-	-	-	-	-	-	-	-	-	-	-	-	-	-	-
AX-147208277	Aradu.A01	3171768	2sten	2sten	2sten	2sten	2sten	2sten	2sten	2sten	-	-	-	-	-	-	-	-	-	-	-	-	-	-	-	-	-	-	-	-	-	-	-	-	-	-
AX-147208313	Aradu.A01	3335135	2sten	2sten	2sten	2sten	2sten	2sten	2sten	2sten	-	-	-	-	-	-	-	-	-	-	-	-	-	-	-	-	-	-	-	-	-	-	-	-	-	-
AX-147208320	Aradu.A01	3362282	2sten	2sten	2sten	2sten	2sten	2sten	2sten	2sten	-	-	-	-	-	-	-	-	-	-	-	-	-	-	-	-	-	-	-	-	-	-	-	-	-	-
AX-147208356	Aradu.A01	3454619	2sten	2sten	2sten	2sten	2sten	2sten	2sten	2sten	-	-	-	-	-	-	-	-	-	-	-	-	-	-	-	-	-	-	-	-	-	-	-	-	-	-
AX-147208419	Aradu.A01	3648281	2sten	2sten	2sten	2sten	2sten	2sten	2sten	2sten	-	-	-	-	-	-	-	-	-	-	-	-	-	-	-	-	-	-	-	-	-	-	-	-	-	-
AX-147208422	Aradu.A01	3665532	2sten	2sten	2sten	2sten	2sten	2sten	2sten	2sten	-	-	-	-	-	-	-	-	-	-	-	-	-	-	-	-	-	-	-	-	-	-	-	-	-	-
AX-147208459	Aradu.A01	3857435	2sten	2sten	2sten	2sten	2sten	2sten	2sten	2sten	-	-	-	-	-	-	-	-	-	-	-	-	-	-	-	-	-	-	-	-	-	-	-	-	-	-
AX-147208477	Aradu.A01	3947625	2sten	2sten	2sten	2sten	2sten	2sten	2sten	2sten	-	-	-	-	-	-	-	-	-	-	-	-	-	-	-	-	-	-	-	-	-	-	-	-	-	-
AX-147208523	Aradu.A01	4143689	2sten	2sten	2sten	2sten	2sten	2sten	2sten	2sten	-	-	-	-	-	-	-	-	-	-	-	-	-	-	-	-	-	-	-	-	-	-	-	-	-	-

SNP ID	Chromosome ID	Start	*A. stenosperma* V 13840	*A. stenosperma* V 13844	*A. stenosperma* V 13844	*A. stenosperma* HLK-410	*A. stenosperma* V 7762	*A. stenosperma* V 10309	*A. stenosperma* V 10309	*A. stenosperma* V 13796	Xingu Of 115	Xingu Of 115	Xingu Of 115	Xingu Of 120	Xingu Of 120	Xingu Of 120	Xingu Of 122	Xingu Of 122	Xingu Of 126	Xingu Of 126	Xingu Of 126	Xingu Of 128	Xingu Of 128	Xingu Of 128	Tif-5-646-10	TifGp-2	Tif-13-1014	Tifguard	Tifguard	Tifrunner	Tifrunner	IAC-OL4	IAC-Runner-886	IAC-Runner-886	IAC Tatu-ST	IAC Tatu-ST
AX-147208570	Aradu.A01	4471984	2sten	2sten	2sten	2sten	2sten	2sten	2sten	2sten	-	-	-	-	-	-	-	-	-	-	-	-	-	-	-	-	-	-	-	-	-	-	-	-	-	-
AX-147208592	Aradu.A01	4568777	2sten	2sten	2sten	2sten	2sten	2sten	2sten	2sten	-	-	-	-	-	-	-	-	-	-	-	-	-	-	-	-	-	-	-	-	-	-	-	-	-	-
AX-147208614	Aradu.A01	4678347	2sten	2sten	2sten	2sten	2sten	2sten	2sten	2sten	-	-	-	-	-	-	-	-	-	-	-	-	-	-	-	-	-	-	-	-	-	-	-	-	-	-
AX-147208681	Aradu.A01	4956409	2sten	2sten	2sten	2sten	2sten	2sten	2sten	2sten	-	-	-	-	-	-	-	-	-	-	-	-	-	-	-	-	-	-	-	-	-	-	-	-	-	-
AX-147208686	Aradu.A01	4971685	2sten	2sten	2sten	2sten	2sten	2sten	2sten	2sten	-	-	-	-	-	-	-	-	-	-	-	-	-	-	-	-	-	-	-	-	-	-	-	-	-	-
AX-147208687	Aradu.A01	4971721	2sten	2sten	2sten	2sten	2sten	2sten	2sten	2sten	-	-	-	-	-	-	-	-	-	-	-	-	-	-	-	-	-	-	-	-	-	-	-	-	-	-
AX-147208902	Aradu.A01	6200623	2sten	2sten	2sten	2sten	2sten	2sten	2sten	2sten	-	-	-	-	-	-	-	-	-	-	-	-	-	-	-	-	-	-	-	-	-	-	-	-	-	-
AX-147208929	Aradu.A01	6337694	2sten	2sten	2sten	2sten	2sten	2sten	2sten	2sten	-	-	-	-	-	-	-	-	-	-	-	-	-	-	-	-	-	-	-	-	-	-	-	-	-	-
AX-147208980	Aradu.A01	6679049	2sten	2sten	2sten	2sten	2sten	2sten	2sten	2sten	-	-	-	-	-	-	-	-	-	-	-	-	-	-	-	-	-	-	-	-	-	-	-	-	-	-
AX-147208981	Aradu.A01	6679291	2sten	2sten	2sten	2sten	2sten	2sten	2sten	2sten	-	-	-	-	-	-	-	-	-	-	-	-	-	-	-	-	-	-	-	-	-	-	-	-	-	-
AX-147208984	Aradu.A01	6679582	2sten	2sten	2sten	2sten	2sten	2sten	2sten	2sten	-	-	-	-	-	-	-	-	-	-	-	-	-	-	-	-	-	-	-	-	-	-	-	-	-	-
AX-147209001	Aradu.A01	6794449	2sten	2sten	2sten	2sten	2sten	2sten	2sten	2sten	-	-	-	-	-	-	-	-	-	-	-	-	-	-	-	-	-	-	-	-	-	-	-	-	-	-
AX-147209005	Aradu.A01	6797580	2sten	2sten	2sten	2sten	2sten	2sten	2sten	2sten	-	-	-	-	-	-	-	-	-	-	-	-	-	-	-	-	-	-	-	-	-	-	-	-	-	-

**SNP ID**	**Chromosome ID**	**Start**	***A. stenosperma* V 13840**	***A. stenosperma* V 13844**	***A. stenosperma* V 13844**	***A. stenosperma* HLK-410**	***A. stenosperma* V 7762**	***A. stenosperma* V 10309**	***A. stenosperma* V 10309**	***A. stenosperma* V 13796**	**Xingu Of 115**	**Xingu Of 115**	**Xingu Of 115**	**Xingu Of 120**	**Xingu Of 120**	**Xingu Of 120**	**Xingu Of 122**	**Xingu Of 122**	**Xingu Of 126**	**Xingu Of 126**	**Xingu Of 126**	**Xingu Of 128**	**Xingu Of 128**	**Xingu Of 128**	**Tif-5-646-10**	**TifGp-2**	**Tif-13-1014**	**Tifguard**	**Tifguard**	**Tifrunner**	**Tifrunner**	**IAC-OL4**	**IAC-Runner-886**	**IAC-Runner-886**	**IAC Tatu-ST**	**IAC Tatu-ST**
AX-147209047	Aradu.A01	7002783	2sten	2sten	2sten	2sten	2sten	2sten	2sten	2sten	-	-	-	-	-	-	-	-	-	-	-	-	-	-	-	-	-	-	-	-	-	-	-	-	-	-
AX-147209077	Aradu.A01	7260640	2sten	2sten	2sten	2sten	2sten	2sten	2sten	2sten	-	-	-	-	-	-	-	-	-	-	-	-	-	-	-	-	-	-	-	-	-	-	-	-	-	-
AX-147209151	Aradu.A01	7777990	2sten	2sten	2sten	2sten	2sten	2sten	2sten	2sten	-	-	-	-	-	-	-	-	-	-	-	-	-	-	-	-	-	-	-	-	-	-	-	-	-	-
AX-147209153	Aradu.A01	7778354	2sten	2sten	2sten	2sten	2sten	2sten	2sten	2sten	-	-	-	-	-	-	-	-	-	-	-	-	-	-	-	-	-	-	-	-	-	-	-	-	-	-
AX-147209175	Aradu.A01	7840759	2sten	2sten	2sten	2sten	2sten	2sten	2sten	2sten	-	-	-	-	-	-	-	-	-	-	-	-	-	-	-	-	-	-	-	-	-	-	-	-	-	-
AX-147209294	Aradu.A01	8483053	2sten	2sten	2sten	2sten	2sten	2sten	2sten	2sten	-	-	-	-	-	-	-	-	-	-	-	-	-	-	-	-	-	-	-	-	-	-	-	-	-	-
AX-147209535	Aradu.A01	9616507	2sten	2sten	2sten	2sten	2sten	2sten	2sten	2sten	-	-	-	-	-	-	-	-	-	-	-	-	-	-	-	-	-	-	-	-	-	-	-	-	-	-
AX-147209562	Aradu.A01	9683560	2sten	2sten	2sten	2sten	2sten	2sten	2sten	2sten	-	-	-	-	-	-	-	-	-	-	-	-	-	-	-	-	-	-	-	-	-	-	-	-	-	-
AX-147209569	Aradu.A01	9732809	2sten	2sten	2sten	2sten	2sten	2sten	2sten	2sten	-	-	-	-	-	-	-	-	-	-	-	-	-	-	-	-	-	-	-	-	-	-	-	-	-	-
AX-147209744	Aradu.A01	10362805	2sten	2sten	2sten	2sten	2sten	2sten	2sten	2sten	-	-	-	-	-	-	-	-	-	-	-	-	-	-	-	-	-	-	-	-	-	-	-	-	-	-
AX-147209746	Aradu.A01	10363502	2sten	2sten	2sten	2sten	2sten	2sten	2sten	2sten	-	-	-	-	-	-	-	-	-	-	-	-	-	-	-	-	-	-	-	-	-	-	-	-	-	-
AX-147209768	Aradu.A01	10400308	2sten	2sten	2sten	2sten	2sten	2sten	2sten	2sten	-	-	-	-	-	-	-	-	-	-	-	-	-	-	-	-	-	-	-	-	-	-	-	-	-	-
AX-147209795	Aradu.A01	10513962	2sten	2sten	2sten	2sten	2sten	2sten	2sten	2sten	-	-	-	-	-	-	-	-	-	-	-	-	-	-	-	-	-	-	-	-	-	-	-	-	-	-

SNP ID	Chromosome ID	Start	*A. stenosperma* V 13840	*A. stenosperma* V 13844	*A. stenosperma* V 13844	*A. stenosperma* HLK-410	*A. stenosperma* V 7762	*A. stenosperma* V 10309	*A. stenosperma* V 10309	*A. stenosperma* V 13796	Xingu Of 115	Xingu Of 115	Xingu Of 115	Xingu Of 120	Xingu Of 120	Xingu Of 120	Xingu Of 122	Xingu Of 122	Xingu Of 126	Xingu Of 126	Xingu Of 126	Xingu Of 128	Xingu Of 128	Xingu Of 128	Tif-5-646-10	TifGp-2	Tif-13-1014	Tifguard	Tifguard	Tifrunner	Tifrunner	IAC-OL4	IAC-Runner-886	IAC-Runner-886	IAC Tatu-ST	IAC Tatu-ST
AX-147209871	Aradu.A01	10877795	2sten	2sten	2sten	2sten	2sten	2sten	2sten	2sten	-	-	-	-	-	-	-	-	-	-	-	-	-	-	-	-	-	-	-	-	-	-	-	-	-	-
AX-147209889	Aradu.A01	10951412	2sten	2sten	2sten	2sten	2sten	2sten	2sten	2sten	-	-	-	-	-	-	-	-	-	-	-	-	-	-	-	-	-	-	-	-	-	-	-	-	-	-
AX-147209909	Aradu.A01	11117584	2sten	2sten	2sten	2sten	2sten	2sten	2sten	2sten	-	-	-	-	-	-	-	-	-	-	-	-	-	-	-	-	-	-	-	-	-	-	-	-	-	-
AX-147209955	Aradu.A01	11376441	2sten	2sten	2sten	2sten	2sten	2sten	2sten	2sten	-	-	-	-	-	-	-	-	-	-	-	-	-	-	-	-	-	-	-	-	-	-	-	-	-	-
AX-147209957	Aradu.A01	11401809	2sten	2sten	2sten	2sten	2sten	2sten	2sten	2sten	-	-	-	-	-	-	-	-	-	-	-	-	-	-	-	-	-	-	-	-	-	-	-	-	-	-
AX-147209963	Aradu.A01	11604379	2sten	2sten	2sten	2sten	2sten	2sten	2sten	2sten	-	-	-	-	-	-	-	-	-	-	-	-	-	-	-	-	-	-	-	-	-	-	-	-	-	-
AX-147209973	Aradu.A01	11617233	2sten	2sten	2sten	2sten	2sten	2sten	2sten	2sten	-	-	-	-	-	-	-	-	-	-	-	-	-	-	-	-	-	-	-	-	-	-	-	-	-	-
AX-147210038	Aradu.A01	11922444	2sten	2sten	2sten	2sten	2sten	2sten	2sten	2sten	-	-	-	-	-	-	-	-	-	-	-	-	-	-	-	-	-	-	-	-	-	-	-	-	-	-
AX-147210045	Aradu.A01	11970977	2sten	2sten	2sten	2sten	2sten	2sten	2sten	2sten	-	-	-	-	-	-	-	-	-	-	-	-	-	-	-	-	-	-	-	-	-	-	-	-	-	-
AX-147210055	Aradu.A01	12011733	2sten	2sten	2sten	2sten	2sten	2sten	2sten	2sten	-	-	-	-	-	-	-	-	-	-	-	-	-	-	-	-	-	-	-	-	-	-	-	-	-	-
AX-147210114	Aradu.A01	12593804	2sten	2sten	2sten	2sten	2sten	2sten	2sten	2sten	-	-	-	-	-	-	-	-	-	-	-	-	-	-	-	-	-	-	-	-	-	-	-	-	-	-
AX-147210119	Aradu.A01	12610883	2sten	2sten	2sten	2sten	2sten	2sten	2sten	2sten	-	-	-	-	-	-	-	-	-	-	-	-	-	-	-	-	-	-	-	-	-	-	-	-	-	-
AX-147210176	Aradu.A01	14044056	2sten	2sten	2sten	2sten	2sten	2sten	2sten	2sten	-	-	-	-	-	-	-	-	-	-	-	-	-	-	-	-	-	-	-	-	-	-	-	-	-	-

SNP ID	Chromosome ID	Start	*A. stenosperma* V 13840	*A. stenosperma* V 13844	*A. stenosperma* V 13844	*A. stenosperma* HLK-410	*A. stenosperma* V 7762	*A. stenosperma* V 10309	*A. stenosperma* V 10309	*A. stenosperma* V 13796	Xingu Of 115	Xingu Of 115	Xingu Of 115	Xingu Of 120	Xingu Of 120	Xingu Of 120	Xingu Of 122	Xingu Of 122	Xingu Of 126	Xingu Of 126	Xingu Of 126	Xingu Of 128	Xingu Of 128	Xingu Of 128	Tif-5-646-10	TifGp-2	Tif-13-1014	Tifguard	Tifguard	Tifrunner	Tifrunner	IAC-OL4	IAC-Runner-886	IAC-Runner-886	IAC Tatu-ST	IAC Tatu-ST
AX-147210198	Aradu.A01	14652680	2sten	2sten	2sten	2sten	2sten	2sten	2sten	2sten	-	-	-	-	-	-	-	-	-	-	-	-	-	-	-	-	-	-	-	-	-	-	-	-	-	-
AX-147210210	Aradu.A01	14895494	2sten	2sten	2sten	2sten	2sten	2sten	2sten	2sten	-	-	-	-	-	-	-	-	1sten	-	-	-	-	-	-	-	-	-	-	-	-	-	-	-	-	-
AX-147210213	Aradu.A01	14897372	2sten	2sten	2sten	2sten	2sten	2sten	2sten	2sten	-	-	-	-	-	-	-	-	-	-	-	-	-	-	-	-	-	-	-	-	-	-	-	-	-	-
AX-147210375	Aradu.A01	19206554	2sten	2sten	2sten	2sten	2sten	2sten	2sten	2sten	-	-	-	-	-	-	-	-	1sten	-	-	-	-	-	-	-	-	-	-	-	-	-	-	-	-	-
AX-147210408	Aradu.A01	19878431	2sten	2sten	2sten	2sten	2sten	2sten	2sten	2sten	-	-	-	-	-	-	-	-	-	-	-	-	-	-	-	-	-	-	-	-	-	-	-	-	-	-
AX-147210423	Aradu.A01	20833178	2sten	2sten	2sten	2sten	2sten	2sten	2sten	2sten	-	-	-	-	-	-	-	-	-	-	-	-	-	-	-	-	-	-	-	-	-	-	-	-	-	-
AX-147210432	Aradu.A01	21307948	2sten	2sten	2sten	2sten	2sten	2sten	2sten	2sten	-	-	-	-	-	-	-	-	-	-	-	-	-	-	-	-	-	-	-	-	-	-	-	-	-	-
AX-147210510	Aradu.A01	23821516	2sten	2sten	2sten	2sten	2sten	2sten	2sten	2sten	-	-	-	-	-	-	-	-	-	-	-	-	-	-	-	-	-	-	-	-	-	-	-	-	-	-
AX-147210581	Aradu.A01	27742123	2sten	2sten	2sten	2sten	2sten	2sten	2sten	2sten	-	-	-	-	-	-	-	-	-	-	-	-	-	-	-	-	-	-	-	-	-	-	-	-	-	-
AX-147210595	Aradu.A01	28148996	2sten	2sten	2sten	2sten	2sten	2sten	2sten	2sten	-	-	-	-	-	-	-	-	-	-	-	-	-	-	-	-	-	-	-	-	-	-	-	-	-	-
AX-176791728	Aradu.A01	28191821	2sten	2sten	2sten	2sten	2sten	2sten	2sten	2sten	-	-	-	-	-	-	-	-	-	-	-	-	-	-	-	-	-	-	-	-	-	-	-	-	-	-
AX-147210685	Aradu.A01	30700005	2sten	2sten	2sten	2sten	2sten	2sten	2sten	2sten	-	-	-	-	-	-	-	-	-	-	-	-	-	-	-	-	-	-	-	-	-	-	-	-	-	-
AX-147210754	Aradu.A01	32558478	2sten	2sten	2sten	2sten	2sten	2sten	2sten	2sten	-	-	-	-	-	-	-	-	-	-	-	-	-	-	-	-	-	-	-	-	-	-	-	-	-	-

SNP ID	Chromosome ID	Start	*A. stenosperma* V 13840	*A. stenosperma* V 13844	*A. stenosperma* V 13844	*A. stenosperma* HLK-410	*A. stenosperma* V 7762	*A. stenosperma* V 10309	*A. stenosperma* V 10309	*A. stenosperma* V 13796	Xingu Of 115	Xingu Of 115	Xingu Of 115	Xingu Of 120	Xingu Of 120	Xingu Of 120	Xingu Of 122	Xingu Of 122	Xingu Of 126	Xingu Of 126	Xingu Of 126	Xingu Of 128	Xingu Of 128	Xingu Of 128	Tif-5-646-10	TifGp-2	Tif-13-1014	Tifguard	Tifguard	Tifrunner	Tifrunner	IAC-OL4	IAC-Runner-886	IAC-Runner-886	IAC Tatu-ST	IAC Tatu-ST
AX-147210868	Aradu.A01	36142296	2sten	2sten	2sten	2sten	2sten	2sten	2sten	2sten	-	-	-	-	-	-	-	-	-	-	-	-	-	-	-	-	-	-	-	-	-	-	-	-	-	-
AX-147211069	Aradu.A01	44307624	2sten	2sten	2sten	2sten	2sten	2sten	2sten	2sten	-	-	-	-	-	-	-	-	-	-	-	-	-	-	-	-	-	-	-	-	-	-	-	-	-	-
AX-147211171	Aradu.A01	49885268	2sten	2sten	2sten	2sten	2sten	2sten	2sten	2sten	-	-	-	-	-	-	-	-	-	NN	-	-	-	-	-	-	-	-	-	-	-	-	-	-	-	-
AX-147211193	Aradu.A01	50260398	2sten	2sten	2sten	2sten	2sten	2sten	2sten	2sten	-	-	-	-	-	-	-	-	-	-	-	-	-	-	-	-	-	-	-	-	-	-	-	-	-	-
AX-147211204	Aradu.A01	50383228	2sten	2sten	2sten	2sten	2sten	2sten	2sten	2sten	-	-	-	-	-	-	-	-	-	-	-	-	-	-	-	-	-	-	-	-	-	-	-	-	-	-
AX-147211289	Aradu.A01	54777219	2sten	2sten	2sten	2sten	2sten	2sten	2sten	2sten	-	-	-	-	-	-	-	-	-	-	-	-	-	-	-	-	-	-	-	-	-	-	-	-	-	-
AX-147211452	Aradu.A01	64545074	2sten	2sten	2sten	2sten	2sten	2sten	2sten	2sten	-	-	-	-	-	-	-	-	-	-	-	-	-	-	-	-	-	-	-	-	-	-	-	-	-	-
AX-176792021	Aradu.A01	67371790	2sten	2sten	2sten	2sten	2sten	2sten	2sten	2sten	-	-	-	-	-	-	-	-	-	-	-	-	-	-	-	-	-	-	-	-	-	-	-	-	-	-
AX-147211616	Aradu.A01	85136905	2sten	2sten	2sten	2sten	2sten	2sten	2sten	2sten	-	-	-	-	-	-	-	-	-	-	-	-	-	-	-	-	-	-	-	-	-	-	-	-	-	-
AX-147211618	Aradu.A01	87096940	2sten	2sten	2sten	2sten	2sten	2sten	2sten	2sten	-	-	-	-	-	-	-	-	-	-	-	-	-	-	-	-	-	-	-	-	-	-	-	-	-	-
AX-147211655	Aradu.A01	89685816	2sten	2sten	2sten	2sten	2sten	2sten	2sten	2sten	-	-	-	-	-	-	-	-	-	-	-	-	-	-	-	-	-	-	-	-	-	-	-	-	-	-
AX-147211698	Aradu.A01	91743384	2sten	2sten	2sten	2sten	2sten	2sten	2sten	2sten	-	-	-	-	-	-	-	-	-	-	-	-	-	-	-	-	-	-	-	-	-	-	-	-	-	-
AX-147211714	Aradu.A01	92478955	2sten	2sten	2sten	2sten	2sten	2sten	2sten	2sten	-	-	-	-	-	-	-	-	-	-	-	-	-	-	-	-	-	-	-	-	-	-	-	-	-	-

SNP ID	Chromosome ID	Start	*A. stenosperma* V 13840	*A. stenosperma* V 13844	*A. stenosperma* V 13844	*A. stenosperma* HLK-410	*A. stenosperma* V 7762	*A. stenosperma* V 10309	*A. stenosperma* V 10309	*A. stenosperma* V 13796	Xingu Of 115	Xingu Of 115	Xingu Of 115	Xingu Of 120	Xingu Of 120	Xingu Of 120	Xingu Of 122	Xingu Of 122	Xingu Of 126	Xingu Of 126	Xingu Of 126	Xingu Of 128	Xingu Of 128	Xingu Of 128	Tif-5-646-10	TifGp-2	Tif-13-1014	Tifguard	Tifguard	Tifrunner	Tifrunner	IAC-OL4	IAC-Runner-886	IAC-Runner-886	IAC Tatu-ST	IAC Tatu-ST
AX-147211718	Aradu.A01	92596099	2sten	2sten	2sten	2sten	2sten	2sten	2sten	2sten	-	-	-	1sten	-	-	-	-	-	-	-	-	-	-	-	-	-	-	-	-	-	-	-	-	-	-
AX-147211814	Aradu.A01	95704622	2sten	2sten	2sten	2sten	2sten	2sten	2sten	2sten	-	-	-	-	-	-	-	-	-	-	-	-	-	-	-	-	-	-	-	-	-	-	-	-	-	-
AX-147211929	Aradu.A01	100451939	2sten	2sten	2sten	2sten	2sten	2sten	2sten	2sten	-	-	-	-	-	-	-	-	-	-	-	-	-	-	-	-	-	-	-	-	-	-	-	-	-	-
AX-147211986	Aradu.A01	102053494	2sten	2sten	2sten	2sten	2sten	2sten	2sten	2sten	-	-	-	-	-	-	-	-	-	-	-	-	-	-	-	-	-	-	-	-	-	-	-	-	-	-
AX-176792555	Aradu.A01	104855194	2sten	2sten	2sten	2sten	2sten	2sten	2sten	2sten	-	-	-	-	-	-	-	-	-	-	-	-	-	-	-	-	-	-	-	-	-	-	-	-	-	-
AX-147212094	Aradu.A01	106856758	2sten	2sten	2sten	2sten	2sten	2sten	2sten	2sten	-	-	-	-	-	-	-	-	-	-	-	-	-	-	-	-	-	-	-	-	-	-	-	-	-	-
AX-147212176	Aradu.A02	327160	2sten	2sten	2sten	2sten	2sten	2sten	2sten	2sten	-	-	-	-	-	-	-	-	-	-	-	-	-	-	-	-	-	-	-	-	-	-	-	-	-	-
AX-147212177	Aradu.A02	328289	2sten	2sten	2sten	2sten	2sten	2sten	2sten	2sten	-	-	-	-	-	-	-	-	-	-	-	-	-	-	-	-	-	-	-	-	-	-	-	-	-	-
AX-176820323	Aradu.A02	351242	2sten	2sten	2sten	2sten	2sten	2sten	2sten	2sten	-	-	-	-	-	-	-	-	-	-	-	-	-	-	-	-	-	-	-	-	-	-	-	-	-	-
AX-147212220	Aradu.A02	529266	2sten	2sten	2sten	2sten	2sten	2sten	2sten	2sten	-	-	-	-	-	-	-	-	-	-	-	-	-	-	-	-	-	-	-	-	-	-	-	-	-	-
AX-176823510	Aradu.A02	611723	2sten	2sten	2sten	2sten	2sten	2sten	2sten	2sten	-	-	-	-	-	-	-	-	-	-	-	-	-	-	-	-	-	-	-	-	-	-	-	-	-	-
AX-176821132	Aradu.A02	1173968	2sten	2sten	2sten	2sten	2sten	2sten	2sten	2sten	-	-	-	-	-	-	-	-	-	-	-	-	-	-	-	-	-	-	-	-	-	-	-	-	-	-
AX-147212659	Aradu.A02	2557281	2sten	2sten	2sten	2sten	2sten	2sten	2sten	2sten	-	-	-	-	-	-	-	-	-	-	-	-	-	-	-	-	-	-	-	-	-	-	-	-	-	-

SNP ID	Chromosome ID	Start	*A. stenosperma* V 13840	*A. stenosperma* V 13844	*A. stenosperma* V 13844	*A. stenosperma* HLK-410	*A. stenosperma* V 7762	*A. stenosperma* V 10309	*A. stenosperma* V 10309	*A. stenosperma* V 13796	Xingu Of 115	Xingu Of 115	Xingu Of 115	Xingu Of 120	Xingu Of 120	Xingu Of 120	Xingu Of 122	Xingu Of 122	Xingu Of 126	Xingu Of 126	Xingu Of 126	Xingu Of 128	Xingu Of 128	Xingu Of 128	Tif-5-646-10	TifGp-2	Tif-13-1014	Tifguard	Tifguard	Tifrunner	Tifrunner	IAC-OL4	IAC-Runner-886	IAC-Runner-886	IAC Tatu-ST	IAC Tatu-ST
AX-147212669	Aradu.A02	2594443	2sten	2sten	2sten	2sten	2sten	2sten	2sten	2sten	-	-	-	-	-	-	-	-	-	-	-	-	-	-	-	-	-	-	-	-	-	-	-	-	-	-
AX-176821041	Aradu.A02	2599915	2sten	2sten	2sten	2sten	2sten	2sten	2sten	2sten	-	-	-	-	-	-	-	-	-	-	-	-	-	-	-	-	-	-	-	-	-	-	-	-	-	-
AX-147212687	Aradu.A02	2640779	2sten	2sten	2sten	2sten	2sten	2sten	2sten	2sten	-	-	-	-	-	-	-	-	-	-	-	-	-	-	-	-	-	-	-	-	-	-	-	-	-	-
AX-176819218	Aradu.A02	2782248	2sten	2sten	2sten	2sten	2sten	2sten	2sten	2sten	-	-	-	-	-	-	-	-	-	-	-	-	-	-	-	-	-	-	-	-	-	-	-	-	-	-
AX-147240219	Aradu.A02	2825568	2sten	2sten	2sten	2sten	2sten	2sten	2sten	2sten	-	-	-	-	-	-	-	-	-	-	-	-	-	-	-	-	-	-	-	-	-	-	-	-	-	-
AX-176822631	Aradu.A02	3047937	2sten	2sten	2sten	2sten	2sten	2sten	2sten	2sten	-	-	-	-	-	-	-	-	-	-	-	-	-	-	-	-	-	-	-	-	-	-	-	-	-	-
AX-176820632	Aradu.A02	3049512	2sten	2sten	2sten	2sten	2sten	2sten	2sten	2sten	-	-	-	-	-	-	-	-	-	-	-	-	-	-	-	-	-	-	-	-	-	-	-	-	-	-
AX-147240478	Aradu.A02	3939035	2sten	2sten	2sten	2sten	2sten	2sten	2sten	2sten	-	-	-	-	-	-	-	-	-	-	-	-	-	-	-	-	-	-	-	-	-	-	-	-	-	-
AX-147240497	Aradu.A02	4085059	2sten	2sten	2sten	2sten	2sten	2sten	2sten	2sten	-	-	-	-	-	-	-	-	-	-	-	-	-	-	-	-	-	-	-	-	-	-	-	-	-	-
AX-147240530	Aradu.A02	4723506	2sten	2sten	2sten	2sten	2sten	2sten	2sten	2sten	-	-	-	-	-	-	-	-	-	-	-	-	-	-	-	-	-	-	-	-	-	-	-	-	-	-
AX-147240532	Aradu.A02	4724850	2sten	2sten	2sten	2sten	2sten	2sten	2sten	2sten	-	-	-	-	-	-	-	-	-	-	-	-	-	-	-	-	-	-	-	-	-	-	-	-	-	-
AX-147212940	Aradu.A02	4851310	2sten	2sten	2sten	2sten	2sten	2sten	2sten	2sten	-	-	-	-	-	-	-	-	-	-	-	-	-	-	-	-	-	-	-	-	-	-	-	-	-	-
AX-147212952	Aradu.A02	4883604	2sten	2sten	2sten	2sten	2sten	2sten	2sten	2sten	-	-	-	-	-	-	-	-	-	-	-	-	-	-	-	-	-	-	-	-	-	-	-	-	-	-

SNP ID	Chromosome ID	Start	*A. stenosperma* V 13840	*A. stenosperma* V 13844	*A. stenosperma* V 13844	*A. stenosperma* HLK-410	*A. stenosperma* V 7762	*A. stenosperma* V 10309	*A. stenosperma* V 10309	*A. stenosperma* V 13796	Xingu Of 115	Xingu Of 115	Xingu Of 115	Xingu Of 120	Xingu Of 120	Xingu Of 120	Xingu Of 122	Xingu Of 122	Xingu Of 126	Xingu Of 126	Xingu Of 126	Xingu Of 128	Xingu Of 128	Xingu Of 128	Tif-5-646-10	TifGp-2	Tif-13-1014	Tifguard	Tifguard	Tifrunner	Tifrunner	IAC-OL4	IAC-Runner-886	IAC-Runner-886	IAC Tatu-ST	IAC Tatu-ST
AX-176792125	Aradu.A02	4899125	2sten	2sten	2sten	2sten	2sten	2sten	2sten	2sten	-	-	-	-	-	-	-	-	-	-	-	-	-	-	-	-	-	-	-	-	-	-	-	-	-	-
AX-147212981	Aradu.A02	5238668	2sten	2sten	2sten	2sten	2sten	2sten	2sten	2sten	-	-	-	-	-	-	-	-	-	-	-	-	-	-	-	-	-	-	-	-	-	-	-	-	-	-
AX-147212990	Aradu.A02	5270191	2sten	2sten	2sten	2sten	2sten	2sten	2sten	2sten	-	-	-	-	-	-	-	-	-	-	-	-	-	-	-	-	-	-	-	-	-	-	-	-	-	-
AX-147213014	Aradu.A02	5535408	2sten	2sten	2sten	2sten	2sten	2sten	2sten	2sten	-	-	-	-	-	-	-	-	-	-	-	-	-	-	-	-	-	-	-	-	-	-	-	-	-	-
AX-177643693	Aradu.A02	5796224	2sten	2sten	2sten	2sten	2sten	2sten	2sten	2sten	-	-	-	-	-	-	-	-	-	-	-	-	-	-	-	-	-	-	-	-	-	-	-	-	-	-
AX-147213119	Aradu.A02	6341115	2sten	2sten	2sten	2sten	2sten	2sten	2sten	2sten	-	-	-	-	-	-	-	-	-	-	-	-	-	-	-	-	-	-	-	-	-	-	-	-	-	-
AX-147213155	Aradu.A02	6827791	2sten	2sten	2sten	2sten	2sten	2sten	2sten	2sten	-	-	-	-	-	-	-	-	-	-	-	-	-	-	-	-	-	-	-	-	-	-	-	-	-	-
AX-147213245	Aradu.A02	8377950	2sten	2sten	2sten	2sten	2sten	2sten	2sten	2sten	-	-	-	-	-	-	-	-	-	-	-	-	-	-	-	-	-	-	-	-	-	-	-	-	-	-
AX-147213292	Aradu.A02	8798047	2sten	2sten	2sten	2sten	2sten	2sten	2sten	2sten	-	-	-	-	-	-	-	-	-	-	-	-	-	-	-	-	-	-	-	-	-	-	-	-	-	-
AX-147213472	Aradu.A02	14937650	2sten	2sten	2sten	2sten	2sten	2sten	2sten	2sten	-	-	-	-	-	-	-	-	-	-	-	-	-	-	-	-	-	-	-	-	-	-	-	-	-	-
AX-147213476	Aradu.A02	14943798	2sten	2sten	2sten	2sten	2sten	2sten	2sten	2sten	-	-	-	-	-	-	-	-	-	-	-	-	-	-	-	-	-	-	-	-	-	-	-	-	-	-
AX-147213537	Aradu.A02	19123495	2sten	2sten	2sten	2sten	2sten	2sten	2sten	2sten	-	-	-	-	-	-	-	-	-	-	-	-	-	-	-	-	-	-	-	-	-	-	-	-	-	-
AX-147213617	Aradu.A02	28328431	2sten	2sten	2sten	2sten	2sten	2sten	2sten	2sten	-	-	-	-	-	-	-	-	-	-	-	-	-	-	-	-	-	-	-	-	-	-	-	-	-	-

SNP ID	Chromosome ID	Start	*A. stenosperma* V 13840	*A. stenosperma* V 13844	*A. stenosperma* V 13844	*A. stenosperma* HLK-410	*A. stenosperma* V 7762	*A. stenosperma* V 10309	*A. stenosperma* V 10309	*A. stenosperma* V 13796	Xingu Of 115	Xingu Of 115	Xingu Of 115	Xingu Of 120	Xingu Of 120	Xingu Of 120	Xingu Of 122	Xingu Of 122	Xingu Of 126	Xingu Of 126	Xingu Of 126	Xingu Of 128	Xingu Of 128	Xingu Of 128	Tif-5-646-10	TifGp-2	Tif-13-1014	Tifguard	Tifguard	Tifrunner	Tifrunner	IAC-OL4	IAC-Runner-886	IAC-Runner-886	IAC Tatu-ST	IAC Tatu-ST
AX-147213631	Aradu.A02	28574705	2sten	2sten	2sten	2sten	2sten	2sten	2sten	2sten	-	-	-	-	-	-	-	-	-	-	-	-	-	-	-	-	-	-	-	-	-	-	-	-	-	-
AX-147213672	Aradu.A02	36455438	2sten	2sten	2sten	2sten	2sten	2sten	2sten	2sten	-	-	-	-	-	-	-	-	-	-	-	-	-	-	-	-	-	-	-	-	-	-	-	-	-	-
AX-147213689	Aradu.A02	39267669	2sten	2sten	2sten	2sten	2sten	2sten	2sten	2sten	-	-	-	-	-	-	-	-	-	-	-	-	-	-	-	-	-	-	-	-	-	-	-	-	-	-
AX-147213736	Aradu.A02	43374913	2sten	2sten	2sten	2sten	2sten	2sten	2sten	2sten	-	-	-	-	-	-	-	-	-	-	-	-	-	-	-	-	-	-	-	-	-	-	-	-	-	-
AX-147213756	Aradu.A02	47309187	2sten	2sten	2sten	2sten	2sten	2sten	2sten	2sten	-	-	-	-	-	-	-	-	-	-	-	-	-	-	-	-	-	-	-	-	-	-	-	-	-	-
AX-176810943	Aradu.A02	50000826	2sten	2sten	2sten	2sten	2sten	2sten	2sten	2sten	-	-	-	-	-	-	-	-	-	-	-	-	-	-	-	-	-	-	-	-	-	-	-	-	-	-
AX-147213789	Aradu.A02	52237413	2sten	2sten	2sten	2sten	2sten	2sten	2sten	2sten	-	-	-	-	-	-	-	-	-	-	-	-	-	-	-	-	-	-	-	-	-	-	-	-	-	-
AX-176791701	Aradu.A02	57307605	2sten	2sten	2sten	2sten	2sten	2sten	2sten	2sten	-	-	-	-	-	-	-	-	-	-	-	-	-	-	-	-	-	-	-	-	-	-	-	-	-	-
AX-147214185	Aradu.A02	75731951	2sten	2sten	2sten	2sten	2sten	2sten	2sten	2sten	-	-	-	-	-	-	-	-	-	-	-	-	-	-	-	-	-	-	-	-	-	-	-	-	-	-
AX-147214234	Aradu.A02	78187888	2sten	2sten	2sten	2sten	2sten	2sten	2sten	2sten	-	-	-	-	-	-	-	-	-	-	-	-	-	-	-	-	-	-	-	-	-	-	-	-	-	-
AX-147214521	Aradu.A02	81609749	2sten	2sten	2sten	2sten	2sten	2sten	2sten	2sten	-	-	-	-	-	-	-	-	-	-	-	-	-	-	-	-	-	-	-	-	-	-	-	-	-	-
AX-147214665	Aradu.A02	83464160	2sten	2sten	2sten	2sten	2sten	2sten	2sten	2sten	-	-	-	-	-	-	-	-	-	-	-	-	-	-	-	-	-	-	-	-	-	-	-	-	-	-
AX-147242208	Aradu.A02	84880919	2sten	2sten	2sten	2sten	2sten	2sten	2sten	2sten	-	-	-	-	-	-	-	-	-	-	-	-	-	-	-	-	-	-	-	-	-	-	-	-	-	-

SNP ID	Chromosome ID	Start	*A. stenosperma* V 13840	*A. stenosperma* V 13844	*A. stenosperma* V 13844	*A. stenosperma* HLK-410	*A. stenosperma* V 7762	*A. stenosperma* V 10309	*A. stenosperma* V 10309	*A. stenosperma* V 13796	Xingu Of 115	Xingu Of 115	Xingu Of 115	Xingu Of 120	Xingu Of 120	Xingu Of 120	Xingu Of 122	Xingu Of 122	Xingu Of 126	Xingu Of 126	Xingu Of 126	Xingu Of 128	Xingu Of 128	Xingu Of 128	Tif-5-646-10	TifGp-2	Tif-13-1014	Tifguard	Tifguard	Tifrunner	Tifrunner	IAC-OL4	IAC-Runner-886	IAC-Runner-886	IAC Tatu-ST	IAC Tatu-ST
AX-147214809	Aradu.A02	85315330	2sten	2sten	2sten	2sten	2sten	2sten	2sten	2sten	-	-	-	-	-	-	-	-	-	-	-	-	-	-	-	-	-	-	-	-	-	-	-	-	-	-
AX-147215232	Aradu.A02	92983757	2sten	2sten	2sten	2sten	2sten	2sten	2sten	2sten	-	-	-	-	-	-	-	-	-	-	-	-	-	-	-	-	-	-	-	-	-	-	-	-	-	-
AX-147215287	Aradu.A03	263764	2sten	2sten	2sten	2sten	2sten	2sten	2sten	2sten	-	-	-	-	-	-	-	-	-	-	-	-	-	-	-	-	-	-	-	-	-	-	-	-	-	-
AX-147215321	Aradu.A03	679369	2sten	2sten	2sten	2sten	2sten	2sten	2sten	2sten	-	-	-	-	-	-	-	-	-	-	-	-	-	-	-	-	-	-	-	-	-	-	-	-	-	-
AX-147215426	Aradu.A03	1989488	2sten	2sten	2sten	2sten	2sten	2sten	2sten	2sten	-	-	-	-	-	-	-	-	-	-	-	-	-	-	-	-	-	-	-	-	-	-	-	-	-	-
AX-147215435	Aradu.A03	2018385	2sten	2sten	2sten	2sten	2sten	2sten	2sten	2sten	-	-	-	-	-	-	-	-	-	-	-	-	-	-	-	-	-	-	-	-	-	-	-	-	-	-
AX-147215631	Aradu.A03	4763077	2sten	2sten	2sten	2sten	2sten	2sten	2sten	2sten	-	-	-	-	-	-	-	-	-	-	-	-	-	-	-	-	-	-	-	-	-	-	-	-	-	-
AX-147215901	Aradu.A03	9964556	2sten	2sten	2sten	2sten	2sten	2sten	2sten	2sten	-	-	-	-	-	-	-	-	-	-	-	-	-	-	-	-	-	-	-	-	-	-	-	-	-	-
AX-147216005	Aradu.A03	12100885	2sten	2sten	2sten	2sten	2sten	2sten	2sten	2sten	-	-	-	-	-	-	-	-	-	-	-	-	-	-	-	-	-	-	-	-	-	-	-	-	-	-
AX-147216011	Aradu.A03	12163932	2sten	2sten	2sten	2sten	2sten	2sten	2sten	2sten	-	-	-	-	-	-	-	-	-	-	-	-	-	-	-	-	-	-	-	-	-	-	-	-	-	-
AX-147216035	Aradu.A03	12860226	2sten	2sten	2sten	2sten	2sten	2sten	2sten	2sten	-	-	-	-	-	-	-	-	-	-	-	-	-	-	-	-	-	-	-	-	-	-	-	-	-	-
AX-147243932	Aradu.A03	13770476	2sten	2sten	2sten	2sten	2sten	2sten	2sten	2sten	-	-	-	-	-	-	-	-	-	-	-	-	-	-	-	-	-	-	-	-	-	-	-	-	-	-
AX-147216341	Aradu.A03	22722541	2sten	2sten	2sten	2sten	2sten	2sten	2sten	2sten	-	-	-	-	-	-	-	-	NN	-	-	-	-	-	-	-	-	-	-	-	-	-	-	-	-	-

SNP ID	Chromosome ID	Start	*A. stenosperma* V 13840	*A. stenosperma* V 13844	*A. stenosperma* V 13844	*A. stenosperma* HLK-410	*A. stenosperma* V 7762	*A. stenosperma* V 10309	*A. stenosperma* V 10309	*A. stenosperma* V 13796	Xingu Of 115	Xingu Of 115	Xingu Of 115	Xingu Of 120	Xingu Of 120	Xingu Of 120	Xingu Of 122	Xingu Of 122	Xingu Of 126	Xingu Of 126	Xingu Of 126	Xingu Of 128	Xingu Of 128	Xingu Of 128	Tif-5-646-10	TifGp-2	Tif-13-1014	Tifguard	Tifguard	Tifrunner	Tifrunner	IAC-OL4	IAC-Runner-886	IAC-Runner-886	IAC Tatu-ST	IAC Tatu-ST
AX-147216499	Aradu.A03	27510595	2sten	2sten	2sten	2sten	2sten	2sten	2sten	2sten	-	-	-	-	-	-	-	-	-	-	-	-	-	-	-	-	-	-	-	-	-	-	-	-	-	-
AX-147216537	Aradu.A03	28985341	2sten	2sten	2sten	2sten	2sten	2sten	2sten	2sten	-	-	-	-	-	-	-	-	-	-	-	-	-	-	-	-	-	-	-	-	-	-	-	-	-	-
AX-176797025	Aradu.A03	32032708	2sten	2sten	2sten	2sten	2sten	2sten	2sten	2sten	-	-	-	-	-	-	-	-	-	-	-	-	-	-	-	-	-	-	-	-	-	-	-	-	-	-
AX-176800155	Aradu.A03	39354975	2sten	2sten	2sten	2sten	2sten	2sten	2sten	2sten	-	-	-	-	-	-	-	-	-	-	-	-	-	-	-	-	-	-	-	-	-	-	-	-	-	-
AX-147216850	Aradu.A03	40250650	2sten	2sten	2sten	2sten	2sten	2sten	2sten	2sten	-	-	-	-	-	-	-	-	-	-	-	-	-	-	-	-	-	-	-	-	-	-	-	-	-	-
AX-147216857	Aradu.A03	40531349	2sten	2sten	2sten	2sten	2sten	2sten	2sten	2sten	NN	-	-	-	-	-	-	-	-	-	-	-	-	-	-	-	-	-	-	-	-	-	-	-	-	-
AX-147217043	Aradu.A03	71754560	2sten	2sten	2sten	2sten	2sten	2sten	2sten	2sten	-	-	-	-	-	-	-	-	-	-	-	-	-	-	-	-	-	-	-	-	-	-	-	-	-	-
AX-147217102	Aradu.A03	85054268	2sten	2sten	2sten	2sten	2sten	2sten	2sten	2sten	-	-	-	-	-	-	-	-	-	-	-	-	-	-	-	-	-	-	-	-	-	-	-	-	-	-
AX-147217108	Aradu.A03	85255229	2sten	2sten	2sten	2sten	2sten	2sten	2sten	2sten	-	-	-	-	-	-	-	-	-	-	-	-	-	-	-	-	-	-	-	-	-	-	-	-	-	-
AX-147217180	Aradu.A03	100096128	2sten	2sten	2sten	2sten	2sten	2sten	2sten	2sten	-	-	-	-	-	-	-	-	-	-	-	-	-	-	-	-	-	-	-	-	-	-	-	-	-	-
AX-147217191	Aradu.A03	100932497	2sten	2sten	2sten	2sten	2sten	2sten	2sten	2sten	-	-	-	-	-	-	-	-	-	-	-	-	-	-	-	-	-	-	-	-	-	-	-	-	-	-
AX-147217299	Aradu.A03	108830589	2sten	2sten	2sten	2sten	2sten	2sten	2sten	2sten	-	-	-	-	-	-	-	-	-	-	-	-	-	-	-	-	-	-	-	-	-	-	-	-	-	-
AX-176792070	Aradu.A03	112869301	2sten	2sten	2sten	2sten	2sten	2sten	2sten	2sten	-	-	-	-	-	-	-	-	-	-	-	-	-	-	-	-	-	-	-	-	-	-	-	-	-	-

SNP ID	Chromosome ID	Start	*A. stenosperma* V 13840	*A. stenosperma* V 13844	*A. stenosperma* V 13844	*A. stenosperma* HLK-410	*A. stenosperma* V 7762	*A. stenosperma* V 10309	*A. stenosperma* V 10309	*A. stenosperma* V 13796	Xingu Of 115	Xingu Of 115	Xingu Of 115	Xingu Of 120	Xingu Of 120	Xingu Of 120	Xingu Of 122	Xingu Of 122	Xingu Of 126	Xingu Of 126	Xingu Of 126	Xingu Of 128	Xingu Of 128	Xingu Of 128	Tif-5-646-10	TifGp-2	Tif-13-1014	Tifguard	Tifguard	Tifrunner	Tifrunner	IAC-OL4	IAC-Runner-886	IAC-Runner-886	IAC Tatu-ST	IAC Tatu-ST
AX-147245139	Aradu.A03	114594539	2sten	2sten	2sten	2sten	2sten	2sten	2sten	2sten	-	-	-	-	-	-	-	-	-	-	-	-	-	-	-	-	-	-	-	-	-	-	-	-	-	-
AX-147217486	Aradu.A03	115670522	2sten	2sten	2sten	2sten	2sten	2sten	2sten	2sten	-	-	-	-	-	-	-	-	-	-	-	-	-	-	-	-	-	-	-	-	-	-	-	-	-	-
AX-147217578	Aradu.A03	117747673	2sten	2sten	2sten	2sten	2sten	2sten	2sten	2sten	-	-	-	-	-	-	-	-	-	-	-	-	-	-	-	-	-	-	-	-	-	-	-	-	-	-
AX-147217621	Aradu.A03	118410155	2sten	2sten	2sten	2sten	2sten	2sten	2sten	2sten	-	-	-	-	-	-	-	-	-	-	-	-	-	-	-	-	-	-	-	-	-	-	-	-	-	-
AX-147217666	Aradu.A03	119191210	2sten	2sten	2sten	2sten	2sten	2sten	2sten	2sten	-	-	-	-	-	-	-	-	-	-	-	-	-	-	-	-	-	-	-	-	-	-	-	-	-	-
AX-147217690	Aradu.A03	119760103	2sten	2sten	2sten	2sten	2sten	2sten	2sten	2sten	-	-	-	-	-	-	-	-	-	-	-	-	-	-	-	-	-	-	-	-	-	-	-	-	-	-
AX-147218000	Aradu.A03	125668334	2sten	2sten	2sten	2sten	2sten	2sten	2sten	2sten	-	-	-	-	-	-	-	-	-	-	-	-	-	-	-	-	-	-	-	-	-	-	-	-	-	-
AX-147218077	Aradu.A03	127096331	2sten	2sten	2sten	2sten	2sten	2sten	2sten	2sten	-	-	-	-	-	-	-	-	-	-	-	-	-	-	-	-	-	-	-	-	-	-	-	-	-	-
AX-147218089	Aradu.A03	127285244	2sten	2sten	2sten	2sten	2sten	2sten	2sten	2sten	-	-	-	-	-	-	-	-	-	-	-	-	-	-	-	-	-	-	-	-	-	-	-	-	-	-
AX-147218118	Aradu.A03	127612658	2sten	2sten	2sten	2sten	2sten	2sten	2sten	2sten	-	-	-	-	-	-	-	-	-	-	-	-	-	-	-	-	-	-	-	-	-	-	-	-	-	-
AX-147218132	Aradu.A03	127784977	2sten	2sten	2sten	2sten	2sten	2sten	2sten	2sten	-	-	-	-	-	-	-	-	-	-	-	-	-	-	-	-	-	-	-	-	-	-	-	-	-	-
AX-147218152	Aradu.A03	128169949	2sten	2sten	2sten	2sten	2sten	2sten	2sten	2sten	-	-	-	-	-	-	-	-	-	-	-	-	-	-	-	-	-	-	-	-	-	-	-	-	-	-
AX-147218304	Aradu.A03	129649573	2sten	2sten	2sten	2sten	2sten	2sten	2sten	2sten	-	-	-	-	-	-	-	-	-	-	-	-	-	-	-	-	-	-	-	-	-	-	-	-	-	-

SNP ID	Chromosome ID	Start	*A. stenosperma* V 13840	*A. stenosperma* V 13844	*A. stenosperma* V 13844	*A. stenosperma* HLK-410	*A. stenosperma* V 7762	*A. stenosperma* V 10309	*A. stenosperma* V 10309	*A. stenosperma* V 13796	Xingu Of 115	Xingu Of 115	Xingu Of 115	Xingu Of 120	Xingu Of 120	Xingu Of 120	Xingu Of 122	Xingu Of 122	Xingu Of 126	Xingu Of 126	Xingu Of 126	Xingu Of 128	Xingu Of 128	Xingu Of 128	Tif-5-646-10	TifGp-2	Tif-13-1014	Tifguard	Tifguard	Tifrunner	Tifrunner	IAC-OL4	IAC-Runner-886	IAC-Runner-886	IAC Tatu-ST	IAC Tatu-ST
AX-147218409	Aradu.A03	130991322	2sten	2sten	2sten	2sten	2sten	2sten	2sten	2sten	-	-	-	-	-	-	-	-	-	-	-	-	-	-	-	-	-	-	-	-	-	-	-	-	-	-
AX-147218442	Aradu.A03	131479716	2sten	2sten	2sten	2sten	2sten	2sten	2sten	2sten	-	-	-	-	-	-	-	-	-	-	-	-	-	-	-	-	-	-	-	-	-	-	-	-	-	-
AX-147218446	Aradu.A03	131522659	2sten	2sten	2sten	2sten	2sten	2sten	2sten	2sten	-	-	-	-	-	-	-	-	-	-	-	-	-	-	-	-	-	-	-	-	-	-	-	-	-	-
AX-147218741	Aradu.A03	134818482	2sten	2sten	2sten	2sten	2sten	2sten	2sten	2sten	-	-	-	-	-	-	-	-	-	-	-	-	-	-	-	-	-	-	-	-	-	-	-	-	-	-
AX-147218747	Aradu.A04	105918	2sten	2sten	2sten	2sten	2sten	2sten	2sten	2sten	-	-	-	-	-	-	-	-	-	-	-	-	-	-	-	-	-	-	-	-	-	-	-	-	-	-
AX-147218752	Aradu.A04	162877	2sten	2sten	2sten	2sten	2sten	2sten	2sten	2sten	-	-	-	-	-	-	-	-	-	-	-	-	-	-	-	-	-	-	-	-	-	-	-	-	-	-
AX-147218758	Aradu.A04	186335	2sten	2sten	2sten	2sten	2sten	2sten	2sten	2sten	-	-	-	-	-	-	-	-	-	-	-	-	-	-	-	-	-	-	-	-	-	-	-	-	-	-
AX-147218905	Aradu.A04	1330961	2sten	2sten	2sten	2sten	2sten	2sten	2sten	2sten	-	-	-	-	-	-	-	-	-	-	-	-	-	-	-	-	-	-	-	-	-	-	-	-	-	-
AX-147218916	Aradu.A04	1428372	2sten	2sten	2sten	2sten	2sten	2sten	2sten	2sten	-	-	-	-	-	-	-	-	-	-	-	-	-	-	-	-	-	-	-	-	-	-	-	-	-	-
AX-147218960	Aradu.A04	1903340	2sten	2sten	2sten	2sten	2sten	2sten	2sten	2sten	-	-	-	-	-	-	-	-	-	-	-	-	-	-	-	-	-	-	-	-	-	-	-	-	-	-
AX-147218990	Aradu.A04	2341548	2sten	2sten	2sten	2sten	2sten	2sten	2sten	2sten	-	-	-	-	-	-	-	-	-	-	-	-	-	-	-	-	-	-	-	-	-	-	-	-	-	-
AX-147219075	Aradu.A04	3019173	2sten	2sten	2sten	2sten	2sten	2sten	2sten	2sten	-	-	-	-	-	-	-	-	-	-	-	-	-	-	-	-	-	-	-	-	-	-	-	-	-	-
AX-147219127	Aradu.A04	3517864	2sten	2sten	2sten	2sten	2sten	2sten	2sten	2sten	-	-	-	-	-	-	-	-	-	-	-	-	-	-	-	-	-	-	-	-	-	-	-	-	-	-

SNP ID	Chromosome ID	Start	*A. stenosperma* V 13840	*A. stenosperma* V 13844	*A. stenosperma* V 13844	*A. stenosperma* HLK-410	*A. stenosperma* V 7762	*A. stenosperma* V 10309	*A. stenosperma* V 10309	*A. stenosperma* V 13796	Xingu Of 115	Xingu Of 115	Xingu Of 115	Xingu Of 120	Xingu Of 120	Xingu Of 120	Xingu Of 122	Xingu Of 122	Xingu Of 126	Xingu Of 126	Xingu Of 126	Xingu Of 128	Xingu Of 128	Xingu Of 128	Tif-5-646-10	TifGp-2	Tif-13-1014	Tifguard	Tifguard	Tifrunner	Tifrunner	IAC-OL4	IAC-Runner-886	IAC-Runner-886	IAC Tatu-ST	IAC Tatu-ST
AX-147219138	Aradu.A04	3712906	2sten	2sten	2sten	2sten	2sten	2sten	2sten	2sten	-	-	-	-	-	-	-	-	-	-	-	-	-	-	-	-	-	-	-	-	-	-	-	-	-	-
AX-147219161	Aradu.A04	4216072	2sten	2sten	2sten	2sten	2sten	2sten	2sten	2sten	-	-	-	-	-	-	-	-	-	-	-	-	-	-	-	-	-	-	-	-	-	-	-	-	-	-
AX-147219256	Aradu.A04	6528578	2sten	2sten	2sten	2sten	2sten	2sten	2sten	2sten	-	-	-	-	-	-	-	-	-	-	-	-	-	-	-	-	-	-	-	-	-	-	-	-	-	-
AX-147219264	Aradu.A04	6680053	2sten	2sten	2sten	2sten	2sten	2sten	2sten	2sten	-	-	-	-	-	-	-	-	-	-	-	-	-	-	-	-	-	-	-	-	-	-	-	-	-	-
AX-147219321	Aradu.A04	7370841	2sten	2sten	2sten	2sten	2sten	2sten	2sten	2sten	-	-	-	-	-	-	-	-	-	-	-	-	-	-	-	-	-	-	-	-	-	-	-	-	-	-
AX-147219466	Aradu.A04	10403152	2sten	2sten	2sten	2sten	2sten	2sten	2sten	2sten	-	-	-	-	-	-	-	-	-	-	-	-	-	-	-	-	-	-	-	-	-	-	-	-	-	-
AX-147219503	Aradu.A04	12230241	2sten	2sten	2sten	2sten	2sten	2sten	2sten	2sten	-	-	-	-	-	-	-	-	-	-	-	-	-	-	-	-	-	-	-	-	-	-	-	-	-	-
AX-147219535	Aradu.A04	13743291	2sten	2sten	2sten	2sten	2sten	2sten	2sten	2sten	-	-	-	-	-	-	-	-	-	-	-	-	-	-	-	-	-	-	-	-	-	-	-	-	-	-
AX-147219646	Aradu.A04	18406432	2sten	2sten	2sten	2sten	2sten	2sten	2sten	2sten	-	-	-	-	-	-	-	-	-	-	-	-	-	-	-	-	-	-	-	-	-	-	-	-	-	-
AX-147219658	Aradu.A04	19952895	2sten	2sten	2sten	2sten	2sten	2sten	2sten	2sten	-	-	-	-	-	-	-	-	-	-	-	-	-	-	-	-	-	-	-	-	-	-	-	-	-	-
AX-147219833	Aradu.A04	31201545	2sten	2sten	2sten	2sten	2sten	2sten	2sten	2sten	-	-	-	-	-	-	-	-	-	-	-	-	-	-	-	-	-	-	-	-	-	-	-	-	-	-
AX-147220040	Aradu.A04	53185086	2sten	2sten	2sten	2sten	2sten	2sten	2sten	2sten	-	-	-	-	-	-	-	-	-	-	-	-	-	-	-	-	-	-	-	-	-	-	-	-	-	-
AX-176804104	Aradu.A04	60018529	2sten	2sten	2sten	2sten	2sten	2sten	2sten	2sten	-	-	-	-	-	-	-	-	-	-	-	-	-	-	-	-	-	-	-	-	-	-	-	-	-	-

SNP ID	Chromosome ID	Start	*A. stenosperma* V 13840	*A. stenosperma* V 13844	*A. stenosperma* V 13844	*A. stenosperma* HLK-410	*A. stenosperma* V 7762	*A. stenosperma* V 10309	*A. stenosperma* V 10309	*A. stenosperma* V 13796	Xingu Of 115	Xingu Of 115	Xingu Of 115	Xingu Of 120	Xingu Of 120	Xingu Of 120	Xingu Of 122	Xingu Of 122	Xingu Of 126	Xingu Of 126	Xingu Of 126	Xingu Of 128	Xingu Of 128	Xingu Of 128	Tif-5-646-10	TifGp-2	Tif-13-1014	Tifguard	Tifguard	Tifrunner	Tifrunner	IAC-OL4	IAC-Runner-886	IAC-Runner-886	IAC Tatu-ST	IAC Tatu-ST
AX-147220067	Aradu.A04	61632421	2sten	2sten	2sten	2sten	2sten	2sten	2sten	2sten	-	-	-	-	-	-	-	-	-	-	-	-	-	-	-	-	-	-	-	-	-	-	-	-	-	-
AX-147220074	Aradu.A04	62753790	2sten	2sten	2sten	2sten	2sten	2sten	2sten	2sten	-	-	-	-	-	-	-	-	-	-	-	-	-	-	-	-	-	-	-	-	-	-	-	-	-	-
AX-147220154	Aradu.A04	76565838	2sten	2sten	2sten	2sten	2sten	2sten	2sten	2sten	-	-	-	-	-	-	-	-	-	-	-	-	-	-	-	-	-	-	-	-	-	-	-	-	-	-
AX-147220250	Aradu.A04	85249142	2sten	2sten	2sten	2sten	2sten	2sten	2sten	2sten	-	-	-	-	-	-	-	-	-	-	-	-	-	-	-	-	-	-	-	-	-	-	-	-	-	-
AX-147220275	Aradu.A04	88047508	2sten	2sten	2sten	2sten	2sten	2sten	2sten	2sten	-	-	-	-	-	-	-	-	-	-	-	-	-	-	-	-	-	-	-	-	-	-	-	-	-	-
AX-147220481	Aradu.A04	103964397	2sten	2sten	2sten	2sten	2sten	2sten	2sten	2sten	-	-	-	-	-	-	-	-	-	-	-	-	-	-	-	-	-	-	-	-	-	-	-	-	-	-
AX-147220802	Aradu.A04	112876982	2sten	2sten	2sten	2sten	2sten	2sten	2sten	2sten	-	-	-	-	-	-	-	-	-	-	-	-	-	-	-	-	-	-	-	-	-	-	-	-	-	-
AX-147220911	Aradu.A04	115264196	2sten	2sten	2sten	2sten	2sten	2sten	2sten	2sten	-	-	-	-	-	-	-	-	-	-	-	-	-	-	-	-	-	-	-	-	-	-	-	-	-	-
AX-147221205	Aradu.A04	119691532	2sten	2sten	2sten	2sten	2sten	2sten	2sten	2sten	-	-	-	-	-	-	-	-	-	-	-	-	-	-	-	-	-	-	-	-	-	-	-	-	-	-
AX-147221510	Aradu.A05	2776713	2sten	2sten	2sten	2sten	2sten	2sten	2sten	2sten	-	-	-	-	-	-	-	-	-	-	-	-	-	-	-	-	-	-	-	-	-	-	-	-	-	-
AX-147221511	Aradu.A05	2859326	2sten	2sten	2sten	2sten	2sten	2sten	2sten	2sten	-	-	-	-	-	-	-	-	-	-	-	-	-	-	-	-	-	-	-	-	-	-	-	-	-	-
AX-147249011	Aradu.A05	4294221	2sten	2sten	2sten	2sten	2sten	2sten	2sten	2sten	-	-	-	-	-	-	-	-	-	-	-	-	-	-	-	-	-	-	-	-	-	-	-	-	-	-
AX-147221560	Aradu.A05	4536691	2sten	2sten	2sten	2sten	2sten	2sten	2sten	2sten	-	-	-	-	-	-	-	-	-	-	-	-	-	-	-	-	-	-	-	-	-	-	-	-	-	-

SNP ID	Chromosome ID	Start	*A. stenosperma* V 13840	*A. stenosperma* V 13844	*A. stenosperma* V 13844	*A. stenosperma* HLK-410	*A. stenosperma* V 7762	*A. stenosperma* V 10309	*A. stenosperma* V 10309	*A. stenosperma* V 13796	Xingu Of 115	Xingu Of 115	Xingu Of 115	Xingu Of 120	Xingu Of 120	Xingu Of 120	Xingu Of 122	Xingu Of 122	Xingu Of 126	Xingu Of 126	Xingu Of 126	Xingu Of 128	Xingu Of 128	Xingu Of 128	Tif-5-646-10	TifGp-2	Tif-13-1014	Tifguard	Tifguard	Tifrunner	Tifrunner	IAC-OL4	IAC-Runner-886	IAC-Runner-886	IAC Tatu-ST	IAC Tatu-ST
AX-176791991	Aradu.A05	5435274	2sten	2sten	2sten	2sten	2sten	2sten	2sten	2sten	-	-	-	-	-	-	-	-	-	-	-	-	-	-	-	-	-	-	-	-	-	-	-	-	-	-
AX-147221580	Aradu.A05	5802548	2sten	2sten	2sten	2sten	2sten	2sten	2sten	2sten	-	-	-	-	-	-	-	-	-	-	-	-	-	-	-	-	-	-	-	-	-	-	-	-	-	-
AX-147221634	Aradu.A05	6930172	2sten	2sten	2sten	2sten	2sten	2sten	2sten	2sten	-	-	-	-	-	-	-	-	-	-	-	-	-	-	-	-	-	-	-	-	-	-	-	-	-	-
AX-147221688	Aradu.A05	7958529	2sten	2sten	2sten	2sten	2sten	2sten	2sten	2sten	-	-	-	-	-	-	-	-	-	-	-	-	-	-	-	-	-	-	-	-	-	-	-	-	-	-
AX-147221702	Aradu.A05	8422902	2sten	2sten	2sten	2sten	2sten	2sten	2sten	2sten	-	-	-	-	-	-	-	-	1sten	-	-	-	-	-	-	-	-	-	-	-	-	-	-	-	-	-
AX-147221713	Aradu.A05	8621814	2sten	2sten	2sten	2sten	2sten	2sten	2sten	2sten	-	-	-	-	-	-	-	-	-	-	-	-	-	-	-	-	-	-	-	-	-	-	-	-	-	-
AX-147221776	Aradu.A05	10183570	2sten	2sten	2sten	2sten	2sten	2sten	2sten	2sten	-	-	-	-	-	-	-	-	1sten	-	NN	-	-	-	-	-	-	-	-	-	-	-	-	-	-	-
AX-147221803	Aradu.A05	10961866	2sten	2sten	2sten	2sten	2sten	2sten	2sten	2sten	-	-	-	-	-	-	-	-	-	-	-	-	-	-	-	-	-	-	-	-	-	-	-	-	-	-
AX-147221836	Aradu.A05	11851924	2sten	2sten	2sten	2sten	2sten	2sten	2sten	2sten	-	-	-	-	-	-	-	-	-	-	-	-	-	-	-	-	-	-	-	-	-	-	-	-	-	-
AX-147221874	Aradu.A05	13259235	2sten	2sten	2sten	2sten	2sten	2sten	2sten	2sten	-	-	-	-	-	-	-	-	-	-	-	-	-	-	-	-	-	-	-	-	-	-	-	-	-	-
AX-147221995	Aradu.A05	16078919	2sten	2sten	2sten	2sten	2sten	2sten	2sten	2sten	-	-	-	-	-	-	-	-	-	-	-	-	-	-	-	-	-	-	-	-	-	-	-	-	-	-
AX-147222100	Aradu.A05	18621146	2sten	2sten	2sten	2sten	2sten	2sten	2sten	2sten	-	-	-	-	-	-	-	-	-	-	-	-	-	-	-	-	-	-	-	-	-	-	-	-	-	-
AX-147222142	Aradu.A05	20099557	2sten	2sten	2sten	2sten	2sten	2sten	2sten	2sten	-	-	-	-	-	-	-	-	-	-	-	-	-	-	-	-	-	-	-	-	-	-	-	-	-	-

SNP ID	Chromosome ID	Start	*A. stenosperma* V 13840	*A. stenosperma* V 13844	*A. stenosperma* V 13844	*A. stenosperma* HLK-410	*A. stenosperma* V 7762	*A. stenosperma* V 10309	*A. stenosperma* V 10309	*A. stenosperma* V 13796	Xingu Of 115	Xingu Of 115	Xingu Of 115	Xingu Of 120	Xingu Of 120	Xingu Of 120	Xingu Of 122	Xingu Of 122	Xingu Of 126	Xingu Of 126	Xingu Of 126	Xingu Of 128	Xingu Of 128	Xingu Of 128	Tif-5-646-10	TifGp-2	Tif-13-1014	Tifguard	Tifguard	Tifrunner	Tifrunner	IAC-OL4	IAC-Runner-886	IAC-Runner-886	IAC Tatu-ST	IAC Tatu-ST
AX-147222237	Aradu.A05	23757151	2sten	2sten	2sten	2sten	2sten	2sten	2sten	2sten	-	-	-	-	-	-	-	-	-	-	-	-	-	-	-	-	-	-	-	-	-	-	-	-	-	-
AX-147222358	Aradu.A05	32342401	2sten	2sten	2sten	2sten	2sten	2sten	2sten	2sten	-	-	-	-	-	-	-	-	-	-	-	-	-	-	-	-	-	-	-	-	-	-	-	-	-	-
AX-147222496	Aradu.A05	50529846	2sten	2sten	2sten	2sten	2sten	2sten	2sten	2sten	-	-	-	-	-	-	-	-	-	-	-	-	-	-	-	-	-	-	-	-	-	-	-	-	-	-
AX-147222497	Aradu.A05	50577476	2sten	2sten	2sten	2sten	2sten	2sten	2sten	2sten	-	-	-	-	-	-	-	-	-	-	-	-	-	-	-	-	-	-	-	-	-	-	-	-	-	-
AX-176796043	Aradu.A05	81634052	2sten	2sten	2sten	2sten	2sten	2sten	2sten	2sten	-	-	-	-	-	-	-	-	-	-	-	-	-	-	-	-	-	-	-	-	-	-	-	-	NN	NN
AX-147251054	Aradu.A05	84284314	2sten	2sten	2sten	2sten	2sten	2sten	2sten	2sten	-	-	-	-	-	-	-	-	-	-	-	-	-	-	-	-	-	-	-	-	-	-	-	-	-	-
AX-147222936	Aradu.A05	86654583	2sten	2sten	2sten	2sten	2sten	2sten	2sten	2sten	-	-	-	-	-	-	-	-	-	-	-	-	-	-	-	-	-	-	-	-	-	-	-	-	-	-
AX-147223052	Aradu.A05	88665233	2sten	2sten	2sten	2sten	2sten	2sten	2sten	2sten	-	-	-	-	-	-	-	-	-	-	-	-	-	-	-	-	-	-	-	-	-	-	-	-	-	-
AX-147223242	Aradu.A05	92835471	2sten	2sten	2sten	2sten	2sten	2sten	2sten	2sten	-	-	-	-	-	-	-	-	-	-	-	-	-	-	-	-	-	-	-	-	-	-	-	-	-	-
AX-147223371	Aradu.A05	96431970	2sten	2sten	2sten	2sten	2sten	2sten	2sten	2sten	-	-	-	-	-	-	-	-	-	-	-	-	-	-	-	-	-	-	-	-	-	-	-	-	-	-
AX-147223533	Aradu.A05	101084375	2sten	2sten	2sten	2sten	2sten	2sten	2sten	2sten	-	-	-	-	-	-	-	-	-	-	-	-	-	-	-	-	-	-	-	-	-	-	-	-	-	-
AX-147223581	Aradu.A05	102045907	2sten	2sten	2sten	2sten	2sten	2sten	2sten	2sten	-	-	-	-	-	-	-	-	-	-	-	-	-	-	-	-	-	-	-	-	-	-	-	-	-	-
AX-147223696	Aradu.A05	104206442	2sten	2sten	2sten	2sten	2sten	2sten	2sten	2sten	-	-	-	-	-	-	-	-	-	-	-	-	-	-	-	-	-	-	-	-	-	-	-	-	-	-

SNP ID	Chromosome ID	Start	*A. stenosperma* V 13840	*A. stenosperma* V 13844	*A. stenosperma* V 13844	*A. stenosperma* HLK-410	*A. stenosperma* V 7762	*A. stenosperma* V 10309	*A. stenosperma* V 10309	*A. stenosperma* V 13796	Xingu Of 115	Xingu Of 115	Xingu Of 115	Xingu Of 120	Xingu Of 120	Xingu Of 120	Xingu Of 122	Xingu Of 122	Xingu Of 126	Xingu Of 126	Xingu Of 126	Xingu Of 128	Xingu Of 128	Xingu Of 128	Tif-5-646-10	TifGp-2	Tif-13-1014	Tifguard	Tifguard	Tifrunner	Tifrunner	IAC-OL4	IAC-Runner-886	IAC-Runner-886	IAC Tatu-ST	IAC Tatu-ST
AX-147223711	Aradu.A05	104421873	2sten	2sten	2sten	2sten	2sten	2sten	2sten	2sten	-	-	-	-	-	-	-	-	-	-	-	-	-	-	-	-	-	-	-	-	-	-	-	-	-	-
AX-147251419	Aradu.A05	108709502	2sten	2sten	2sten	2sten	2sten	2sten	2sten	2sten	-	-	-	-	-	-	-	-	-	-	-	-	-	-	-	-	-	-	-	-	-	-	-	-	-	-
AX-147224089	Aradu.A06	259899	2sten	2sten	2sten	2sten	2sten	2sten	2sten	2sten	-	-	-	-	-	-	-	-	-	-	-	-	-	-	-	-	-	-	-	-	-	-	-	-	-	-
AX-147224165	Aradu.A06	1222094	2sten	2sten	2sten	2sten	2sten	2sten	2sten	2sten	-	-	-	-	-	-	-	-	-	-	-	-	-	-	-	-	-	-	-	-	-	-	-	-	-	-
AX-147224218	Aradu.A06	1685836	2sten	2sten	2sten	2sten	2sten	2sten	2sten	2sten	-	-	-	-	-	-	-	-	-	-	-	-	-	-	-	-	-	-	-	-	-	-	-	-	-	-
AX-147224248	Aradu.A06	2371285	2sten	2sten	2sten	2sten	2sten	2sten	2sten	2sten	-	-	-	-	-	-	-	-	-	-	-	-	-	-	-	-	-	-	-	-	-	-	-	-	-	-
AX-147224251	Aradu.A06	2375104	2sten	2sten	2sten	2sten	2sten	2sten	2sten	2sten	-	-	-	-	-	-	-	-	-	-	-	-	-	-	-	-	-	-	-	-	-	-	-	-	-	-
AX-147224270	Aradu.A06	2746968	2sten	2sten	2sten	2sten	2sten	2sten	2sten	2sten	-	-	-	-	-	-	-	-	-	-	-	-	-	-	-	-	-	-	-	-	-	-	-	-	-	-
AX-147224280	Aradu.A06	2863917	2sten	2sten	2sten	2sten	2sten	2sten	2sten	2sten	-	-	-	-	-	-	-	-	-	-	-	-	-	-	-	-	-	-	-	-	-	-	-	-	-	-
AX-147224316	Aradu.A06	3474332	2sten	2sten	2sten	2sten	2sten	2sten	2sten	2sten	-	-	-	-	-	-	-	-	-	-	-	-	-	-	-	-	-	-	-	-	-	-	-	-	-	-
AX-147224370	Aradu.A06	4260980	2sten	2sten	2sten	2sten	2sten	2sten	2sten	2sten	-	-	-	-	-	-	-	-	-	-	-	-	-	-	-	-	-	-	-	-	-	-	-	-	-	-
AX-147224372	Aradu.A06	4280748	2sten	2sten	2sten	2sten	2sten	2sten	2sten	2sten	-	-	-	-	-	-	-	-	-	-	-	-	-	-	-	-	-	-	-	-	-	-	-	-	-	-
AX-147224487	Aradu.A06	5411366	2sten	2sten	2sten	2sten	2sten	2sten	2sten	2sten	-	-	-	-	-	-	-	-	-	-	-	-	-	-	-	-	-	-	-	-	-	-	-	-	-	-

SNP ID	Chromosome ID	Start	*A. stenosperma* V 13840	*A. stenosperma* V 13844	*A. stenosperma* V 13844	*A. stenosperma* HLK-410	*A. stenosperma* V 7762	*A. stenosperma* V 10309	*A. stenosperma* V 10309	*A. stenosperma* V 13796	Xingu Of 115	Xingu Of 115	Xingu Of 115	Xingu Of 120	Xingu Of 120	Xingu Of 120	Xingu Of 122	Xingu Of 122	Xingu Of 126	Xingu Of 126	Xingu Of 126	Xingu Of 128	Xingu Of 128	Xingu Of 128	Tif-5-646-10	TifGp-2	Tif-13-1014	Tifguard	Tifguard	Tifrunner	Tifrunner	IAC-OL4	IAC-Runner-886	IAC-Runner-886	IAC Tatu-ST	IAC Tatu-ST
AX-147224507	Aradu.A06	5789201	2sten	2sten	2sten	2sten	2sten	2sten	2sten	2sten	-	-	-	-	-	-	-	-	-	-	-	-	-	-	-	-	-	-	-	-	-	-	-	-	-	-
AX-147224542	Aradu.A06	6374059	2sten	2sten	2sten	2sten	2sten	2sten	2sten	2sten	-	-	-	-	-	-	-	-	-	-	-	-	-	-	-	-	-	-	-	-	-	-	-	-	-	-
AX-147224636	Aradu.A06	7672289	2sten	2sten	2sten	2sten	2sten	2sten	2sten	2sten	-	-	-	-	-	-	-	-	-	-	-	-	-	-	-	-	-	-	-	-	-	-	-	-	-	-
AX-147224664	Aradu.A06	8149614	2sten	2sten	2sten	2sten	2sten	2sten	2sten	2sten	-	-	-	-	-	-	-	-	-	-	-	-	-	-	-	-	-	-	-	-	-	-	-	-	-	-
AX-147224737	Aradu.A06	9574491	2sten	2sten	2sten	2sten	2sten	2sten	2sten	2sten	-	-	-	-	-	-	-	-	-	-	-	-	-	-	-	-	-	-	-	-	-	-	-	-	-	-
AX-147224808	Aradu.A06	10688253	2sten	2sten	2sten	2sten	2sten	2sten	2sten	2sten	-	-	-	-	-	-	-	-	-	-	-	-	-	-	-	-	-	-	-	-	-	-	-	-	-	-
AX-147225104	Aradu.A06	16428309	2sten	2sten	2sten	2sten	2sten	2sten	2sten	2sten	-	-	-	-	-	-	-	-	-	-	-	-	-	-	-	-	-	-	-	-	-	-	-	-	-	-
AX-147225232	Aradu.A06	20970147	2sten	2sten	2sten	2sten	2sten	2sten	2sten	2sten	-	-	-	-	-	-	-	-	-	-	-	-	-	-	-	-	-	-	-	-	-	-	-	-	-	-
AX-147225297	Aradu.A06	26723018	2sten	2sten	2sten	2sten	2sten	2sten	2sten	2sten	-	-	-	-	-	-	-	-	-	-	-	-	-	-	-	-	-	-	-	-	-	-	-	-	-	-
AX-147225325	Aradu.A06	32572622	2sten	2sten	2sten	2sten	2sten	2sten	2sten	2sten	-	-	-	-	-	-	-	-	-	-	-	-	-	-	-	-	-	-	-	-	-	-	-	-	-	-
AX-147225334	Aradu.A06	34826369	2sten	2sten	2sten	2sten	2sten	2sten	2sten	2sten	-	-	-	-	-	-	-	-	-	-	-	-	-	-	-	-	-	-	-	-	-	-	-	-	-	-
AX-177638959	Aradu.A06	41229012	2sten	2sten	2sten	2sten	2sten	2sten	2sten	2sten	-	-	-	-	-	-	-	-	-	-	-	-	-	-	-	-	-	-	-	-	-	-	-	-	-	-
AX-147225443	Aradu.A06	66423193	2sten	2sten	2sten	2sten	2sten	2sten	2sten	2sten	-	-	-	-	-	-	-	-	-	-	-	-	-	-	-	-	-	-	-	-	-	-	-	-	-	-

SNP ID	Chromosome ID	Start	*A. stenosperma* V 13840	*A. stenosperma* V 13844	*A. stenosperma* V 13844	*A. stenosperma* HLK-410	*A. stenosperma* V 7762	*A. stenosperma* V 10309	*A. stenosperma* V 10309	*A. stenosperma* V 13796	Xingu Of 115	Xingu Of 115	Xingu Of 115	Xingu Of 120	Xingu Of 120	Xingu Of 120	Xingu Of 122	Xingu Of 122	Xingu Of 126	Xingu Of 126	Xingu Of 126	Xingu Of 128	Xingu Of 128	Xingu Of 128	Tif-5-646-10	TifGp-2	Tif-13-1014	Tifguard	Tifguard	Tifrunner	Tifrunner	IAC-OL4	IAC-Runner-886	IAC-Runner-886	IAC Tatu-ST	IAC Tatu-ST
AX-147225464	Aradu.A06	69465592	2sten	2sten	2sten	2sten	2sten	2sten	2sten	2sten	-	-	-	-	-	-	-	-	-	-	-	-	-	-	-	-	-	-	-	-	-	-	-	-	-	-
AX-176797223	Aradu.A06	78938720	2sten	2sten	2sten	2sten	2sten	2sten	2sten	2sten	-	-	-	-	-	-	-	-	1sten	-	-	-	-	-	-	-	-	-	-	-	-	-	-	-	-	-
AX-147225594	Aradu.A06	79851444	2sten	2sten	2sten	2sten	2sten	2sten	2sten	2sten	-	-	-	-	-	-	-	-	-	-	-	-	-	-	-	-	-	-	-	-	-	-	-	-	-	-
AX-147225614	Aradu.A06	80755162	2sten	2sten	2sten	2sten	2sten	2sten	2sten	2sten	-	-	-	-	-	-	-	-	-	-	-	-	-	-	-	-	-	-	-	-	-	-	-	-	-	-
AX-147225805	Aradu.A06	91658358	2sten	2sten	2sten	2sten	2sten	2sten	2sten	2sten	-	-	-	-	-	-	-	-	-	-	-	-	-	-	-	-	-	-	-	-	-	-	-	-	-	-
AX-147225935	Aradu.A06	97904331	2sten	2sten	2sten	2sten	2sten	2sten	2sten	2sten	-	-	-	-	-	-	-	-	-	-	-	-	-	-	-	-	-	-	-	-	-	-	-	-	-	-
AX-147226037	Aradu.A06	100317207	2sten	2sten	2sten	2sten	2sten	2sten	2sten	2sten	-	-	-	-	-	-	-	-	-	-	-	-	-	-	-	-	-	-	-	-	-	-	-	-	-	-
AX-147226154	Aradu.A06	102679906	2sten	2sten	2sten	2sten	2sten	2sten	2sten	2sten	-	-	-	-	-	-	-	-	-	-	-	-	-	-	-	-	-	-	-	-	-	-	-	-	-	-
AX-147226178	Aradu.A06	103181399	2sten	2sten	2sten	2sten	2sten	2sten	2sten	2sten	-	-	-	-	-	-	-	-	-	-	-	-	-	-	-	-	-	-	-	-	-	-	-	-	-	-
AX-147226189	Aradu.A06	103342353	2sten	2sten	2sten	2sten	2sten	2sten	2sten	2sten	-	-	-	-	-	-	-	-	-	-	-	-	-	-	-	-	-	-	-	-	-	-	-	-	-	-
AX-147226332	Aradu.A06	105470777	2sten	2sten	2sten	2sten	2sten	2sten	2sten	2sten	-	-	-	-	-	-	-	-	-	-	-	-	-	-	-	-	-	-	-	-	-	-	-	-	-	-
AX-147253944	Aradu.A06	107413089	2sten	2sten	2sten	2sten	2sten	2sten	2sten	2sten	-	-	-	-	-	-	-	-	-	-	-	-	-	-	-	-	-	-	-	-	-	-	-	-	-	-
AX-176800832	Aradu.A06	109385349	2sten	2sten	2sten	2sten	2sten	2sten	2sten	2sten	-	-	-	-	-	-	-	-	-	-	-	-	-	-	-	-	-	-	-	-	-	-	-	-	NN	1sten

SNP ID	Chromosome ID	Start	*A. stenosperma* V 13840	*A. stenosperma* V 13844	*A. stenosperma* V 13844	*A. stenosperma* HLK-410	*A. stenosperma* V 7762	*A. stenosperma* V 10309	*A. stenosperma* V 10309	*A. stenosperma* V 13796	Xingu Of 115	Xingu Of 115	Xingu Of 115	Xingu Of 120	Xingu Of 120	Xingu Of 120	Xingu Of 122	Xingu Of 122	Xingu Of 126	Xingu Of 126	Xingu Of 126	Xingu Of 128	Xingu Of 128	Xingu Of 128	Tif-5-646-10	TifGp-2	Tif-13-1014	Tifguard	Tifguard	Tifrunner	Tifrunner	IAC-OL4	IAC-Runner-886	IAC-Runner-886	IAC Tatu-ST	IAC Tatu-ST
AX-147226816	Aradu.A06	112388789	2sten	2sten	2sten	2sten	2sten	2sten	2sten	2sten	-	-	-	-	-	-	-	-	-	-	-	-	-	-	-	-	-	-	-	-	-	-	-	-	-	-
AX-147226820	Aradu.A06	112447907	2sten	2sten	2sten	2sten	2sten	2sten	2sten	2sten	-	-	-	-	-	-	-	-	-	-	-	-	-	-	-	-	-	-	-	-	-	-	-	-	-	-
AX-147227304	Aradu.A07	4510160	2sten	2sten	2sten	2sten	2sten	2sten	2sten	2sten	-	-	-	-	-	-	-	-	-	-	-	-	-	-	-	-	-	-	-	-	-	-	-	-	-	-
AX-147227318	Aradu.A07	4560031	2sten	2sten	2sten	2sten	2sten	2sten	2sten	2sten	-	-	-	-	-	-	-	-	-	-	-	-	-	-	-	-	-	-	-	-	-	-	-	-	-	-
AX-147227409	Aradu.A07	5513028	2sten	2sten	2sten	2sten	2sten	2sten	2sten	2sten	-	-	-	-	-	-	-	-	-	-	-	-	-	-	-	-	-	-	-	-	-	-	-	-	-	-
AX-147227676	Aradu.A07	10930502	2sten	2sten	2sten	2sten	2sten	2sten	2sten	2sten	-	-	-	-	-	-	-	-	-	-	-	-	-	-	-	-	-	-	-	-	-	-	-	-	-	-
AX-147227833	Aradu.A07	14860252	2sten	2sten	2sten	2sten	2sten	2sten	2sten	2sten	-	-	-	-	-	-	-	-	-	-	-	-	-	-	-	-	-	-	-	-	-	-	-	-	-	-
AX-147227840	Aradu.A07	15199108	2sten	2sten	2sten	2sten	2sten	2sten	2sten	2sten	-	-	-	-	-	-	-	-	-	-	-	-	-	-	-	-	-	-	-	-	-	-	-	-	-	-
AX-147227917	Aradu.A07	17133453	2sten	2sten	2sten	2sten	2sten	2sten	2sten	2sten	-	-	-	-	-	-	-	-	-	-	-	-	-	-	-	-	-	-	-	-	-	-	-	-	-	-
AX-147227919	Aradu.A07	17218119	2sten	2sten	2sten	2sten	2sten	2sten	2sten	2sten	-	-	-	-	-	-	-	-	-	-	-	-	-	-	-	-	-	-	-	-	-	-	-	-	-	-
AX-176791685	Aradu.A07	17302867	2sten	2sten	2sten	2sten	2sten	2sten	2sten	2sten	-	-	-	-	-	-	-	-	-	-	-	-	-	-	-	-	-	-	-	-	-	-	-	-	-	-
AX-147227930	Aradu.A07	17768653	2sten	2sten	2sten	2sten	2sten	2sten	2sten	2sten	-	-	-	-	-	-	-	-	-	-	-	-	-	-	-	-	-	-	-	-	-	-	-	-	-	-
AX-147228073	Aradu.A07	23988111	2sten	2sten	2sten	2sten	2sten	2sten	2sten	2sten	-	-	-	-	-	-	-	-	-	-	-	-	-	-	-	-	-	-	-	-	-	-	-	-	-	-

SNP ID	Chromosome ID	Start	*A. stenosperma* V 13840	*A. stenosperma* V 13844	*A. stenosperma* V 13844	*A. stenosperma* HLK-410	*A. stenosperma* V 7762	*A. stenosperma* V 10309	*A. stenosperma* V 10309	*A. stenosperma* V 13796	Xingu Of 115	Xingu Of 115	Xingu Of 115	Xingu Of 120	Xingu Of 120	Xingu Of 120	Xingu Of 122	Xingu Of 122	Xingu Of 126	Xingu Of 126	Xingu Of 126	Xingu Of 128	Xingu Of 128	Xingu Of 128	Tif-5-646-10	TifGp-2	Tif-13-1014	Tifguard	Tifguard	Tifrunner	Tifrunner	IAC-OL4	IAC-Runner-886	IAC-Runner-886	IAC Tatu-ST	IAC Tatu-ST
AX-147228146	Aradu.A07	28320876	2sten	2sten	2sten	2sten	2sten	2sten	2sten	2sten	-	-	-	-	-	-	-	-	-	-	-	-	-	-	-	-	-	-	-	-	-	-	-	-	-	-
AX-147228270	Aradu.A07	38770162	2sten	2sten	2sten	2sten	2sten	2sten	2sten	2sten	-	-	-	-	-	-	-	-	-	-	-	-	-	-	-	-	-	-	-	-	-	-	-	-	-	-
AX-147228382	Aradu.A07	49949832	2sten	2sten	2sten	2sten	2sten	2sten	2sten	2sten	-	-	-	-	-	-	-	-	-	-	-	-	-	-	-	-	-	-	-	-	-	-	-	-	-	-
AX-147228566	Aradu.A07	61354208	2sten	2sten	2sten	2sten	2sten	2sten	2sten	2sten	-	-	-	-	-	-	-	-	-	-	-	-	-	-	-	-	-	-	-	-	-	-	-	-	-	-
AX-147228576	Aradu.A07	62398204	2sten	2sten	2sten	2sten	2sten	2sten	2sten	2sten	-	-	-	-	-	-	-	-	-	-	-	-	-	-	-	-	-	-	-	-	-	-	-	-	-	-
AX-147228586	Aradu.A07	63674864	2sten	2sten	2sten	2sten	2sten	2sten	2sten	2sten	-	-	-	-	-	-	-	-	-	-	-	-	-	-	-	-	-	-	-	-	-	-	-	-	-	-
AX-147228634	Aradu.A07	65957751	2sten	2sten	2sten	2sten	2sten	2sten	2sten	2sten	-	-	-	-	-	-	-	-	-	-	-	-	-	-	-	-	-	-	-	-	-	-	-	-	-	-
AX-147228738	Aradu.A07	69596006	2sten	2sten	2sten	2sten	2sten	2sten	2sten	2sten	-	-	-	-	-	-	-	-	-	-	-	-	-	-	-	-	-	-	-	-	-	-	-	-	-	-
AX-147228759	Aradu.A07	70123535	2sten	2sten	2sten	2sten	2sten	2sten	2sten	2sten	-	-	-	-	-	-	-	-	-	-	-	-	-	-	-	-	-	-	-	-	-	-	-	-	-	-
AX-147228782	Aradu.A07	71054246	2sten	2sten	2sten	2sten	2sten	2sten	2sten	2sten	-	-	-	-	-	-	-	-	-	-	-	-	-	-	-	-	-	-	-	-	-	-	-	-	-	-
AX-147229051	Aradu.A07	76344644	2sten	2sten	2sten	2sten	2sten	2sten	2sten	2sten	-	-	-	-	-	-	-	-	-	-	-	-	-	-	-	-	-	-	-	-	-	-	-	-	-	-
AX-147229255	Aradu.A08	2897316	2sten	2sten	2sten	2sten	2sten	2sten	2sten	2sten	-	-	-	-	-	-	-	-	-	-	-	-	-	-	-	-	-	-	-	-	-	-	-	-	-	-
AX-147255960	Aradu.A08	3402445	2sten	2sten	2sten	2sten	2sten	2sten	2sten	2sten	-	-	-	-	-	-	-	-	-	-	-	-	-	-	-	-	-	-	-	-	-	-	-	-	2sten	2sten

SNP ID	Chromosome ID	Start	*A. stenosperma* V 13840	*A. stenosperma* V 13844	*A. stenosperma* V 13844	*A. stenosperma* HLK-410	*A. stenosperma* V 7762	*A. stenosperma* V 10309	*A. stenosperma* V 10309	*A. stenosperma* V 13796	Xingu Of 115	Xingu Of 115	Xingu Of 115	Xingu Of 120	Xingu Of 120	Xingu Of 120	Xingu Of 122	Xingu Of 122	Xingu Of 126	Xingu Of 126	Xingu Of 126	Xingu Of 128	Xingu Of 128	Xingu Of 128	Tif-5-646-10	TifGp-2	Tif-13-1014	Tifguard	Tifguard	Tifrunner	Tifrunner	IAC-OL4	IAC-Runner-886	IAC-Runner-886	IAC Tatu-ST	IAC Tatu-ST
AX-147229410	Aradu.A08	4910287	2sten	2sten	2sten	2sten	2sten	2sten	2sten	2sten	-	-	-	-	-	-	-	-	-	-	-	-	-	-	-	-	-	-	-	-	-	-	-	-	-	-
AX-147229642	Aradu.A08	7337501	2sten	2sten	2sten	2sten	2sten	2sten	2sten	2sten	-	-	-	-	-	-	-	-	-	-	-	-	-	-	-	-	-	-	-	-	-	-	-	-	-	-
AX-147229709	Aradu.A08	8571756	2sten	2sten	2sten	2sten	2sten	2sten	2sten	2sten	-	-	-	-	-	-	-	-	-	-	-	-	-	-	-	-	-	-	-	-	-	-	-	-	-	-
AX-147229729	Aradu.A08	8965991	2sten	2sten	2sten	2sten	2sten	2sten	2sten	2sten	-	-	-	-	-	-	-	-	-	-	-	-	-	-	-	-	-	-	-	-	-	-	-	-	-	-
AX-147229740	Aradu.A08	9346004	2sten	2sten	2sten	2sten	2sten	2sten	2sten	2sten	-	-	-	-	-	-	-	-	-	-	-	-	-	-	-	-	-	-	-	-	-	-	-	-	-	-
AX-147229763	Aradu.A08	10011905	2sten	2sten	2sten	2sten	2sten	2sten	2sten	2sten	-	-	-	-	-	-	-	-	-	-	-	-	-	-	-	-	-	-	-	-	-	-	-	-	-	-
AX-147229813	Aradu.A08	11067184	2sten	2sten	2sten	2sten	2sten	2sten	2sten	2sten	-	-	-	-	-	-	-	-	-	-	-	-	-	-	-	-	-	-	-	-	-	-	-	-	-	-
AX-147229852	Aradu.A08	11713883	2sten	2sten	2sten	2sten	2sten	2sten	2sten	2sten	-	-	-	-	-	-	-	-	-	-	-	-	-	-	-	-	-	-	-	-	-	-	-	-	-	-
AX-147230049	Aradu.A08	15036571	2sten	2sten	2sten	2sten	2sten	2sten	2sten	2sten	-	-	-	-	-	-	-	-	-	-	-	-	-	-	-	-	-	-	-	-	-	-	-	-	-	-
AX-147230087	Aradu.A08	15257408	2sten	2sten	2sten	2sten	2sten	2sten	2sten	2sten	-	-	-	-	-	-	-	-	-	-	-	-	-	-	-	-	-	-	-	-	-	-	-	-	-	-
AX-147230088	Aradu.A08	15257983	2sten	2sten	2sten	2sten	2sten	2sten	2sten	2sten	-	-	-	-	-	-	-	-	-	-	-	-	-	-	-	-	-	-	-	-	-	-	-	-	-	-
AX-147230121	Aradu.A08	15976972	2sten	2sten	2sten	2sten	2sten	2sten	2sten	2sten	-	-	-	-	-	-	-	-	-	-	-	-	-	-	-	-	-	-	-	-	-	-	-	-	-	-
AX-147230197	Aradu.A08	16894818	2sten	2sten	2sten	2sten	2sten	2sten	2sten	2sten	-	-	-	-	-	-	-	-	-	-	-	-	-	-	-	-	-	-	-	-	-	-	-	-	-	-

SNP ID	Chromosome ID	Start	*A. stenosperma* V 13840	*A. stenosperma* V 13844	*A. stenosperma* V 13844	*A. stenosperma* HLK-410	*A. stenosperma* V 7762	*A. stenosperma* V 10309	*A. stenosperma* V 10309	*A. stenosperma* V 13796	Xingu Of 115	Xingu Of 115	Xingu Of 115	Xingu Of 120	Xingu Of 120	Xingu Of 120	Xingu Of 122	Xingu Of 122	Xingu Of 126	Xingu Of 126	Xingu Of 126	Xingu Of 128	Xingu Of 128	Xingu Of 128	Tif-5-646-10	TifGp-2	Tif-13-1014	Tifguard	Tifguard	Tifrunner	Tifrunner	IAC-OL4	IAC-Runner-886	IAC-Runner-886	IAC Tatu-ST	IAC Tatu-ST
AX-147230319	Aradu.A08	23241272	2sten	2sten	2sten	2sten	2sten	2sten	2sten	2sten	-	-	-	-	-	-	-	-	-	-	-	-	-	-	-	-	-	-	-	-	-	-	-	-	-	-
AX-147230419	Aradu.A08	24793024	2sten	2sten	2sten	2sten	2sten	2sten	2sten	2sten	-	-	-	-	-	-	-	-	-	-	-	-	-	-	-	-	-	-	-	-	-	-	-	-	-	-
AX-147230422	Aradu.A08	25003320	2sten	2sten	2sten	2sten	2sten	2sten	2sten	2sten	-	-	-	-	-	-	-	-	-	-	-	-	-	-	-	-	-	-	-	-	-	-	-	-	-	-
AX-147230432	Aradu.A08	25082692	2sten	2sten	2sten	2sten	2sten	2sten	2sten	2sten	-	-	-	-	-	-	-	-	-	-	-	-	-	-	-	-	-	-	-	-	-	-	-	-	-	-
AX-147230437	Aradu.A08	25180565	2sten	2sten	2sten	2sten	2sten	2sten	2sten	2sten	-	-	-	-	-	-	-	-	-	-	-	-	-	-	-	-	-	-	-	-	-	-	-	-	-	-
AX-147230439	Aradu.A08	25222154	2sten	2sten	2sten	2sten	2sten	2sten	2sten	2sten	-	-	-	-	-	-	-	-	-	-	-	-	-	-	-	-	-	-	-	-	-	-	-	-	-	-
AX-147230499	Aradu.A08	26344925	2sten	2sten	2sten	2sten	2sten	2sten	2sten	2sten	-	-	-	-	-	-	-	-	-	-	-	-	-	-	-	-	-	-	-	-	-	-	-	-	-	-
AX-147230507	Aradu.A08	26553664	2sten	2sten	2sten	2sten	2sten	2sten	2sten	2sten	-	-	-	-	-	-	-	-	-	-	-	-	-	-	-	-	-	-	-	-	-	-	-	-	-	-
AX-176791657	Aradu.A08	27581325	2sten	2sten	2sten	2sten	2sten	2sten	2sten	2sten	-	-	-	-	-	-	-	-	-	-	-	-	-	-	-	-	-	-	-	-	-	-	-	-	-	-
AX-147230672	Aradu.A08	29666625	2sten	2sten	2sten	2sten	2sten	2sten	2sten	2sten	-	-	-	-	-	-	-	-	-	-	-	-	-	-	-	-	-	-	-	-	-	-	-	-	-	-
AX-147230776	Aradu.A08	31537516	2sten	2sten	2sten	2sten	2sten	2sten	2sten	2sten	-	-	-	-	-	-	-	-	-	-	-	-	-	-	-	-	-	-	-	-	-	-	-	-	-	-
AX-177643125	Aradu.A08	32566261	2sten	2sten	2sten	2sten	2sten	2sten	2sten	2sten	-	-	-	-	-	-	-	-	-	-	-	-	-	-	-	-	-	-	-	-	-	-	-	-	-	-
AX-147231077	Aradu.A08	37475940	2sten	2sten	2sten	2sten	2sten	2sten	2sten	2sten	-	-	-	-	-	-	-	-	-	-	-	-	-	-	-	-	-	-	-	-	-	-	-	-	-	-

SNP ID	Chromosome ID	Start	*A. stenosperma* V 13840	*A. stenosperma* V 13844	*A. stenosperma* V 13844	*A. stenosperma* HLK-410	*A. stenosperma* V 7762	*A. stenosperma* V 10309	*A. stenosperma* V 10309	*A. stenosperma* V 13796	Xingu Of 115	Xingu Of 115	Xingu Of 115	Xingu Of 120	Xingu Of 120	Xingu Of 120	Xingu Of 122	Xingu Of 122	Xingu Of 126	Xingu Of 126	Xingu Of 126	Xingu Of 128	Xingu Of 128	Xingu Of 128	Tif-5-646-10	TifGp-2	Tif-13-1014	Tifguard	Tifguard	Tifrunner	Tifrunner	IAC-OL4	IAC-Runner-886	IAC-Runner-886	IAC Tatu-ST	IAC Tatu-ST
AX-147231126	Aradu.A08	38180570	2sten	2sten	2sten	2sten	2sten	2sten	2sten	2sten	-	-	-	-	-	-	-	-	-	-	-	-	-	-	-	-	-	-	-	-	-	-	-	-	-	-
AX-147231148	Aradu.A08	38319035	2sten	2sten	2sten	2sten	2sten	2sten	2sten	2sten	-	-	-	-	-	-	-	-	-	-	-	-	-	-	-	-	-	-	-	-	-	-	-	-	-	-
AX-147231204	Aradu.A08	39171398	2sten	2sten	2sten	2sten	2sten	2sten	2sten	2sten	-	-	-	-	-	-	-	-	-	-	-	-	-	-	-	-	-	-	-	-	-	-	-	-	-	-
AX-147231206	Aradu.A08	39208922	2sten	2sten	2sten	2sten	2sten	2sten	2sten	2sten	-	-	-	NN	-	-	-	-	NN	-	-	-	-	-	-	-	-	-	-	-	-	-	-	-	-	-
AX-147258789	Aradu.A08	39271624	2sten	2sten	2sten	2sten	2sten	2sten	2sten	2sten	-	-	-	-	-	-	-	-	-	-	-	-	-	-	-	-	-	-	-	-	-	-	-	-	-	-
AX-147231307	Aradu.A08	40886425	2sten	2sten	2sten	2sten	2sten	2sten	2sten	2sten	-	-	-	-	-	-	-	-	1sten	-	-	-	-	-	-	-	-	-	-	-	-	-	-	-	-	-
AX-177644356	Aradu.A08	41339815	2sten	2sten	2sten	2sten	2sten	2sten	2sten	2sten	-	-	-	-	-	-	-	-	-	-	-	-	-	-	-	-	-	-	-	-	-	-	-	-	-	-
AX-147258971	Aradu.A08	41498901	2sten	2sten	2sten	2sten	2sten	2sten	2sten	2sten	-	-	-	-	-	-	-	-	-	-	-	-	-	-	-	-	-	-	-	-	-	-	-	-	-	-
AX-147231410	Aradu.A08	42383639	2sten	2sten	2sten	2sten	2sten	2sten	2sten	2sten	-	-	-	-	-	-	-	-	-	-	-	-	-	-	-	-	-	-	-	-	-	-	-	-	-	-
AX-147231809	Aradu.A08	46432971	2sten	2sten	2sten	2sten	2sten	2sten	2sten	2sten	-	-	-	-	-	-	-	-	-	-	-	-	-	-	-	-	-	-	-	-	-	-	-	-	-	-
AX-147232070	Aradu.A09	367270	2sten	2sten	2sten	2sten	2sten	2sten	2sten	2sten	-	-	-	-	-	-	-	-	-	-	-	-	-	-	-	-	-	-	-	-	-	-	-	-	-	-
AX-147232078	Aradu.A09	550863	2sten	2sten	2sten	2sten	2sten	2sten	2sten	2sten	-	-	-	-	-	-	-	-	-	-	-	-	-	-	-	-	-	-	-	-	-	-	-	-	-	-
AX-147232337	Aradu.A09	3427098	2sten	2sten	2sten	2sten	2sten	2sten	2sten	2sten	-	-	-	-	-	-	-	-	-	-	-	-	-	-	-	-	-	-	-	-	-	-	-	-	-	-

SNP ID	Chromosome ID	Start	*A. stenosperma* V 13840	*A. stenosperma* V 13844	*A. stenosperma* V 13844	*A. stenosperma* HLK-410	*A. stenosperma* V 7762	*A. stenosperma* V 10309	*A. stenosperma* V 10309	*A. stenosperma* V 13796	Xingu Of 115	Xingu Of 115	Xingu Of 115	Xingu Of 120	Xingu Of 120	Xingu Of 120	Xingu Of 122	Xingu Of 122	Xingu Of 126	Xingu Of 126	Xingu Of 126	Xingu Of 128	Xingu Of 128	Xingu Of 128	Tif-5-646-10	TifGp-2	Tif-13-1014	Tifguard	Tifguard	Tifrunner	Tifrunner	IAC-OL4	IAC-Runner-886	IAC-Runner-886	IAC Tatu-ST	IAC Tatu-ST
AX-147232661	Aradu.A09	8661881	2sten	2sten	2sten	2sten	2sten	2sten	2sten	2sten	-	-	-	-	-	-	-	-	-	-	-	-	-	-	-	-	-	-	-	-	-	-	-	-	-	-
AX-147232700	Aradu.A09	9827197	2sten	2sten	2sten	2sten	2sten	2sten	2sten	2sten	-	-	-	-	-	-	-	-	-	-	-	-	-	-	-	-	-	-	-	-	-	-	-	-	-	-
AX-176791761	Aradu.A09	12546376	2sten	2sten	2sten	2sten	2sten	2sten	2sten	2sten	-	-	-	-	-	-	-	-	-	-	-	-	-	-	-	-	-	-	-	-	-	-	-	-	-	-
AX-147232832	Aradu.A09	13120013	2sten	2sten	2sten	2sten	2sten	2sten	2sten	2sten	-	-	-	-	-	-	-	-	-	-	-	-	-	-	-	-	-	-	-	-	-	-	-	-	-	-
AX-147232906	Aradu.A09	14385375	2sten	2sten	2sten	2sten	2sten	2sten	2sten	2sten	-	-	-	-	-	-	-	-	-	-	-	-	-	-	-	-	-	-	-	-	-	-	-	-	-	-
AX-177639024	Aradu.A09	16385332	2sten	2sten	2sten	2sten	2sten	2sten	2sten	2sten	-	-	-	-	-	-	-	-	-	-	-	-	-	-	-	-	-	-	-	-	-	-	-	-	-	-
AX-147232952	Aradu.A09	16472740	2sten	2sten	2sten	2sten	2sten	2sten	2sten	2sten	-	-	-	-	-	-	-	-	-	-	-	-	-	-	-	-	-	-	1sten	-	-	-	-	-	-	-
AX-147232981	Aradu.A09	17033451	2sten	2sten	2sten	2sten	2sten	2sten	2sten	2sten	-	-	-	-	-	-	-	-	-	-	-	-	-	-	-	-	-	-	-	-	-	-	-	-	-	-
AX-147233079	Aradu.A09	20412693	2sten	2sten	2sten	2sten	2sten	2sten	2sten	2sten	-	-	-	-	-	-	-	-	-	-	-	-	-	-	-	-	-	-	-	-	-	-	-	-	-	-
AX-147233080	Aradu.A09	20416550	2sten	2sten	2sten	2sten	2sten	2sten	2sten	2sten	-	-	-	-	-	-	-	-	1sten	-	-	-	-	-	-	-	-	-	-	-	-	-	-	-	-	-
AX-176797333	Aradu.A09	20677273	2sten	2sten	2sten	2sten	2sten	2sten	2sten	2sten	-	-	-	-	-	-	-	-	-	-	-	-	-	-	-	-	-	-	-	-	-	-	-	-	NN	NN
AX-147233233	Aradu.A09	30827028	2sten	2sten	2sten	2sten	2sten	2sten	2sten	2sten	-	-	-	-	-	-	-	-	-	-	-	-	-	-	-	-	-	-	-	-	-	-	-	-	-	-
AX-147233267	Aradu.A09	35915290	2sten	2sten	2sten	2sten	2sten	2sten	2sten	2sten	-	-	-	-	-	-	-	-	-	-	-	-	-	-	-	-	-	-	-	-	-	-	-	-	-	-

SNP ID	Chromosome ID	Start	*A. stenosperma* V 13840	*A. stenosperma* V 13844	*A. stenosperma* V 13844	*A. stenosperma* HLK-410	*A. stenosperma* V 7762	*A. stenosperma* V 10309	*A. stenosperma* V 10309	*A. stenosperma* V 13796	Xingu Of 115	Xingu Of 115	Xingu Of 115	Xingu Of 120	Xingu Of 120	Xingu Of 120	Xingu Of 122	Xingu Of 122	Xingu Of 126	Xingu Of 126	Xingu Of 126	Xingu Of 128	Xingu Of 128	Xingu Of 128	Tif-5-646-10	TifGp-2	Tif-13-1014	Tifguard	Tifguard	Tifrunner	Tifrunner	IAC-OL4	IAC-Runner-886	IAC-Runner-886	IAC Tatu-ST	IAC Tatu-ST
AX-147233554	Aradu.A09	82366402	2sten	2sten	2sten	2sten	2sten	2sten	2sten	2sten	-	-	-	-	-	-	-	-	-	-	-	-	-	-	-	-	-	-	-	-	-	-	-	-	-	-
AX-147233670	Aradu.A09	94328757	2sten	2sten	2sten	2sten	2sten	2sten	2sten	2sten	-	-	-	-	-	-	-	-	-	-	-	-	-	-	-	-	-	-	-	-	-	-	-	-	-	-
AX-147233736	Aradu.A09	100203509	2sten	2sten	2sten	2sten	2sten	2sten	2sten	2sten	-	-	-	-	-	-	-	-	-	-	-	-	-	-	-	-	-	1sten	1sten	-	-	-	-	-	-	-
AX-147233961	Aradu.A09	108869835	2sten	2sten	2sten	2sten	2sten	2sten	2sten	2sten	-	-	-	-	-	-	-	-	1sten	-	1sten	-	-	-	-	-	-	-	-	-	-	-	-	-	-	-
AX-147233992	Aradu.A09	109699103	2sten	2sten	2sten	2sten	2sten	2sten	2sten	2sten	-	-	-	-	-	-	-	-	-	-	-	-	-	-	-	-	-	-	-	-	-	-	-	-	-	-
AX-147234159	Aradu.A09	111972465	2sten	2sten	2sten	2sten	2sten	2sten	2sten	2sten	-	-	-	-	-	-	-	-	-	-	-	-	-	-	-	-	-	1sten	1sten	-	-	-	-	-	-	-
AX-147234195	Aradu.A09	112245506	2sten	2sten	2sten	2sten	2sten	2sten	2sten	2sten	-	-	-	-	-	-	-	-	-	-	-	-	-	-	-	-	-	1sten	1sten	-	-	-	-	-	-	-
AX-147234232	Aradu.A09	112601250	2sten	2sten	2sten	2sten	2sten	2sten	2sten	2sten	-	-	-	-	-	-	-	-	-	-	-	-	-	-	-	-	-	1sten	1sten	-	-	-	-	-	-	-
AX-147234385	Aradu.A09	114515924	2sten	2sten	2sten	2sten	2sten	2sten	2sten	2sten	-	-	-	-	-	-	-	-	-	-	-	-	-	-	-	-	-	-	-	-	-	-	-	-	-	-
AX-147234444	Aradu.A09	115268567	2sten	2sten	2sten	2sten	2sten	2sten	2sten	2sten	-	-	-	-	-	-	-	-	-	-	-	-	-	-	-	-	-	-	-	-	-	-	-	-	-	-
AX-147262112	Aradu.A09	116264687	2sten	2sten	2sten	2sten	2sten	2sten	2sten	2sten	-	-	-	-	-	-	-	-	-	-	-	-	-	-	-	-	-	-	-	-	-	-	-	-	-	-
AX-147234599	Aradu.A09	116627055	2sten	2sten	2sten	2sten	2sten	2sten	2sten	2sten	-	-	-	-	-	-	-	-	-	-	-	-	-	-	-	-	-	-	-	-	-	-	-	-	-	-
AX-176792163	Aradu.A09	116748794	2sten	2sten	2sten	2sten	2sten	2sten	2sten	2sten	-	-	-	-	-	-	-	-	-	-	-	-	-	-	-	-	-	-	-	-	-	-	-	-	-	-

SNP ID	Chromosome ID	Start	*A. stenosperma* V 13840	*A. stenosperma* V 13844	*A. stenosperma* V 13844	*A. stenosperma* HLK-410	*A. stenosperma* V 7762	*A. stenosperma* V 10309	*A. stenosperma* V 10309	*A. stenosperma* V 13796	Xingu Of 115	Xingu Of 115	Xingu Of 115	Xingu Of 120	Xingu Of 120	Xingu Of 120	Xingu Of 122	Xingu Of 122	Xingu Of 126	Xingu Of 126	Xingu Of 126	Xingu Of 128	Xingu Of 128	Xingu Of 128	Tif-5-646-10	TifGp-2	Tif-13-1014	Tifguard	Tifguard	Tifrunner	Tifrunner	IAC-OL4	IAC-Runner-886	IAC-Runner-886	IAC Tatu-ST	IAC Tatu-ST
AX-177641676	Aradu.A09	117562306	2sten	2sten	2sten	2sten	2sten	2sten	2sten	2sten	-	-	-	-	-	-	-	-	-	-	-	-	-	-	-	-	-	-	-	-	-	-	-	-	1sten	1sten
AX-147234740	Aradu.A09	118577630	2sten	2sten	2sten	2sten	2sten	2sten	2sten	2sten	-	-	-	-	-	-	-	-	-	-	-	-	-	-	-	-	-	-	-	-	-	-	-	-	-	-
AX-147234866	Aradu.A10	373595	2sten	2sten	2sten	2sten	2sten	2sten	2sten	2sten	-	-	-	-	-	-	-	-	-	-	-	-	-	-	-	-	-	-	-	-	-	-	-	-	-	-
AX-147234996	Aradu.A10	2323309	2sten	2sten	2sten	2sten	2sten	2sten	2sten	2sten	-	-	-	-	-	-	-	-	-	-	-	-	-	-	-	-	-	-	-	-	-	-	-	-	-	-
AX-177644136	Aradu.A10	2422728	2sten	2sten	2sten	2sten	2sten	2sten	2sten	2sten	-	-	-	-	-	-	-	-	-	-	-	-	-	-	-	-	-	-	-	-	-	-	-	-	-	-
AX-147235037	Aradu.A10	2883293	2sten	2sten	2sten	2sten	2sten	2sten	2sten	2sten	-	-	-	-	-	-	-	-	-	-	-	-	-	-	-	-	-	-	-	-	-	-	-	-	-	-
AX-147235204	Aradu.A10	4951842	2sten	2sten	2sten	2sten	2sten	2sten	2sten	2sten	-	-	-	-	-	-	-	-	-	-	-	-	-	-	-	-	-	-	-	-	-	-	-	-	-	-
AX-147235274	Aradu.A10	5845893	2sten	2sten	2sten	2sten	2sten	2sten	2sten	2sten	-	-	-	-	-	-	-	-	-	-	-	-	-	-	-	-	-	-	-	-	-	-	-	-	-	-
AX-147235299	Aradu.A10	6103296	2sten	2sten	2sten	2sten	2sten	2sten	2sten	2sten	-	-	-	-	-	-	-	-	-	-	-	-	-	-	-	-	-	-	-	-	-	-	-	-	-	-
AX-147235343	Aradu.A10	6584612	2sten	2sten	2sten	2sten	2sten	2sten	2sten	2sten	-	-	-	-	-	-	-	-	-	-	-	-	-	-	-	-	-	-	-	-	-	-	-	-	-	-
AX-147235363	Aradu.A10	6782065	2sten	2sten	2sten	2sten	2sten	2sten	2sten	2sten	-	-	-	-	-	-	-	-	-	-	-	-	-	-	-	-	-	-	-	-	-	-	-	-	-	-
AX-147235364	Aradu.A10	6784582	2sten	2sten	2sten	2sten	2sten	2sten	2sten	2sten	-	-	-	-	-	-	-	-	NN	-	-	-	-	-	-	-	-	-	-	-	-	-	-	-	-	-
AX-147235513	Aradu.A10	10641846	2sten	2sten	2sten	2sten	2sten	2sten	2sten	2sten	-	-	-	-	-	-	-	-	-	-	-	-	-	-	-	-	-	-	-	-	-	-	-	-	1sten	-

SNP ID	Chromosome ID	Start	*A. stenosperma* V 13840	*A. stenosperma* V 13844	*A. stenosperma* V 13844	*A. stenosperma* HLK-410	*A. stenosperma* V 7762	*A. stenosperma* V 10309	*A. stenosperma* V 10309	*A. stenosperma* V 13796	Xingu Of 115	Xingu Of 115	Xingu Of 115	Xingu Of 120	Xingu Of 120	Xingu Of 120	Xingu Of 122	Xingu Of 122	Xingu Of 126	Xingu Of 126	Xingu Of 126	Xingu Of 128	Xingu Of 128	Xingu Of 128	Tif-5-646-10	TifGp-2	Tif-13-1014	Tifguard	Tifguard	Tifrunner	Tifrunner	IAC-OL4	IAC-Runner-886	IAC-Runner-886	IAC Tatu-ST	IAC Tatu-ST
AX-147235670	Aradu.A10	19686083	2sten	2sten	2sten	2sten	2sten	2sten	2sten	2sten	-	-	-	-	-	-	-	-	-	-	-	-	-	-	-	-	-	-	-	-	-	-	-	-	-	-
AX-176811229	Aradu.A10	22008533	2sten	2sten	2sten	2sten	2sten	2sten	2sten	2sten	-	-	-	-	-	-	-	-	-	-	-	-	-	-	-	-	-	-	-	-	-	-	-	-	1sten	1sten
AX-177642558	Aradu.A10	23775310	2sten	2sten	2sten	2sten	2sten	2sten	2sten	2sten	-	-	-	-	-	-	-	-	-	-	-	-	-	-	-	-	-	-	-	-	-	-	-	-	-	-
AX-147235833	Aradu.A10	34514313	2sten	2sten	2sten	2sten	2sten	2sten	2sten	2sten	-	-	-	-	-	-	-	-	-	-	-	-	-	-	-	-	-	-	-	-	-	-	-	-	-	-
AX-147235962	Aradu.A10	57722531	2sten	2sten	2sten	2sten	2sten	2sten	2sten	2sten	-	-	-	-	-	-	-	-	-	-	-	-	-	-	-	-	-	-	-	-	-	-	-	-	-	-
AX-147235972	Aradu.A10	58678624	2sten	2sten	2sten	2sten	2sten	2sten	2sten	2sten	-	-	-	-	-	-	-	-	-	-	-	-	-	-	-	-	-	-	-	-	-	-	-	-	-	-
AX-147235983	Aradu.A10	62593588	2sten	2sten	2sten	2sten	2sten	2sten	2sten	2sten	-	-	-	-	-	-	-	-	-	-	-	-	-	-	-	-	-	-	-	-	-	-	-	-	-	-
AX-147264133	Aradu.A10	66220759	2sten	2sten	2sten	2sten	2sten	2sten	2sten	2sten	-	-	-	-	-	-	-	-	-	-	-	-	-	-	-	-	-	-	-	-	-	-	-	-	-	-
AX-147236045	Aradu.A10	67382991	2sten	2sten	2sten	2sten	2sten	2sten	2sten	2sten	-	-	-	-	-	-	-	-	-	-	-	-	-	-	-	-	-	-	-	-	-	-	-	-	-	-
AX-147236198	Aradu.A10	83313544	2sten	2sten	2sten	2sten	2sten	2sten	2sten	2sten	-	-	-	-	-	-	-	-	-	-	-	-	-	-	-	-	-	-	-	-	-	-	-	-	-	-
AX-147236222	Aradu.A10	84012865	2sten	2sten	2sten	2sten	2sten	2sten	2sten	2sten	-	-	-	-	-	-	-	-	-	-	-	-	-	-	-	-	-	-	-	-	-	-	-	-	-	-
AX-147236225	Aradu.A10	84240871	2sten	2sten	2sten	2sten	2sten	2sten	2sten	2sten	-	-	-	-	-	-	-	-	-	-	-	-	-	-	-	-	-	-	-	-	-	-	-	-	-	-
AX-147236244	Aradu.A10	85297078	2sten	2sten	2sten	2sten	2sten	2sten	2sten	2sten	-	-	-	-	-	-	-	-	-	-	-	-	-	-	-	-	-	-	-	-	-	-	-	-	-	-

SNP ID	Chromosome ID	Start	*A. stenosperma* V 13840	*A. stenosperma* V 13844	*A. stenosperma* V 13844	*A. stenosperma* HLK-410	*A. stenosperma* V 7762	*A. stenosperma* V 10309	*A. stenosperma* V 10309	*A. stenosperma* V 13796	Xingu Of 115	Xingu Of 115	Xingu Of 115	Xingu Of 120	Xingu Of 120	Xingu Of 120	Xingu Of 122	Xingu Of 122	Xingu Of 126	Xingu Of 126	Xingu Of 126	Xingu Of 128	Xingu Of 128	Xingu Of 128	Tif-5-646-10	TifGp-2	Tif-13-1014	Tifguard	Tifguard	Tifrunner	Tifrunner	IAC-OL4	IAC-Runner-886	IAC-Runner-886	IAC Tatu-ST	IAC Tatu-ST
AX-177640443	Aradu.A10	87711827	2sten	2sten	2sten	2sten	2sten	2sten	2sten	2sten	-	-	-	-	-	-	-	-	-	-	-	-	-	-	-	-	-	-	-	-	-	-	-	-	1sten	1sten
AX-147236291	Aradu.A10	88475585	2sten	2sten	2sten	2sten	2sten	2sten	2sten	2sten	-	-	-	-	-	-	-	-	-	-	-	-	-	-	-	-	-	-	-	-	-	-	-	-	-	-
AX-147236571	Aradu.A10	99172678	2sten	2sten	2sten	2sten	2sten	2sten	2sten	2sten	-	-	-	-	-	-	-	-	-	-	-	-	-	-	-	-	-	-	-	-	-	-	-	-	-	-
AX-147236642	Aradu.A10	100698699	2sten	2sten	2sten	2sten	2sten	2sten	2sten	2sten	-	-	-	-	-	-	-	-	-	-	-	-	-	-	-	-	-	-	-	-	-	-	-	-	-	-
AX-176791584	Aradu.A10	101233562	2sten	2sten	2sten	2sten	2sten	2sten	2sten	2sten	-	-	-	-	-	-	-	-	-	-	-	-	-	-	-	-	-	-	-	-	-	-	-	-	-	-
AX-147236792	Aradu.A10	103586828	2sten	2sten	2sten	2sten	2sten	2sten	2sten	2sten	-	-	-	-	-	-	-	-	-	-	-	-	-	-	-	-	-	-	-	-	-	-	-	-	-	-
AX-147236821	Aradu.A10	104138416	2sten	2sten	2sten	2sten	2sten	2sten	2sten	2sten	-	-	-	-	-	-	-	-	-	-	-	-	-	-	-	-	-	-	-	-	-	-	-	-	-	-
AX-147236829	Aradu.A10	104321843	2sten	2sten	2sten	2sten	2sten	2sten	2sten	2sten	-	-	-	-	-	-	-	-	-	-	-	-	-	-	-	-	-	-	-	-	-	-	-	-	-	-
AX-147236852	Aradu.A10	104660189	2sten	2sten	2sten	2sten	2sten	2sten	2sten	2sten	-	-	-	-	-	-	-	-	-	-	-	-	-	-	-	-	-	-	-	-	-	-	-	-	-	-
AX-147236916	Aradu.A10	105361983	2sten	2sten	2sten	2sten	2sten	2sten	2sten	2sten	-	-	-	1sten	-	-	-	-	1sten	-	-	-	-	-	-	-	-	-	-	-	-	-	-	-	-	-
AX-147236928	Aradu.A10	105535519	2sten	2sten	2sten	2sten	2sten	2sten	2sten	2sten	-	-	-	-	-	-	-	-	-	-	-	-	-	-	-	-	-	-	-	-	-	-	-	-	-	-
AX-147237116	Aradu.A10	107338645	2sten	2sten	2sten	2sten	2sten	2sten	2sten	2sten	-	-	-	-	-	-	-	-	-	-	-	-	-	-	-	-	-	-	-	-	-	-	-	-	-	-
AX-147237141	Aradu.A10	107485077	2sten	2sten	2sten	2sten	2sten	2sten	2sten	2sten	-	-	-	-	-	-	-	-	-	-	-	-	-	-	-	-	-	-	-	-	-	-	-	-	-	-

SNP ID	Chromosome ID	Start	*A. stenosperma* V 13840	*A. stenosperma* V 13844	*A. stenosperma* V 13844	*A. stenosperma* HLK-410	*A. stenosperma* V 7762	*A. stenosperma* V 10309	*A. stenosperma* V 10309	*A. stenosperma* V 13796	Xingu Of 115	Xingu Of 115	Xingu Of 115	Xingu Of 120	Xingu Of 120	Xingu Of 120	Xingu Of 122	Xingu Of 122	Xingu Of 126	Xingu Of 126	Xingu Of 126	Xingu Of 128	Xingu Of 128	Xingu Of 128	Tif-5-646-10	TifGp-2	Tif-13-1014	Tifguard	Tifguard	Tifrunner	Tifrunner	IAC-OL4	IAC-Runner-886	IAC-Runner-886	IAC Tatu-ST	IAC Tatu-ST
AX-147237210	Aradu.A10	107885719	2sten	2sten	2sten	2sten	2sten	2sten	2sten	2sten	-	-	-	-	-	-	-	-	-	-	-	-	-	-	-	-	-	-	-	-	-	-	-	-	-	-
AX-147237245	Aradu.A10	108153380	2sten	2sten	2sten	2sten	2sten	2sten	2sten	2sten	-	-	-	-	-	-	-	-	-	-	-	-	-	-	-	-	-	-	-	-	-	-	-	-	-	-
AX-147255729	#N/D	#N/D	2sten	2sten	2sten	2sten	2sten	2sten	2sten	2sten	-	-	-	-	-	-	-	-	-	-	-	-	-	-	-	-	-	-	-	-	-	-	-	-	-	-
AX-147231118	#N/D	#N/D	2sten	2sten	2sten	2sten	2sten	2sten	2sten	2sten	-	-	-	-	-	-	-	-	-	-	-	-	-	-	-	-	-	-	-	-	-	-	-	-	-	-
AX-147238563	#N/D	#N/D	2sten	2sten	2sten	2sten	2sten	2sten	2sten	2sten	-	-	-	-	-	-	-	-	-	-	-	-	-	-	-	-	-	-	-	-	-	-	-	-	-	-
AX-147264134	#N/D	#N/D	2sten	2sten	2sten	2sten	2sten	2sten	2sten	2sten	-	-	-	-	-	-	-	-	-	-	-	-	-	-	-	-	-	-	-	-	-	-	-	-	2sten	2sten
AX-147240547	#N/D	#N/D	2sten	2sten	2sten	2sten	2sten	2sten	2sten	2sten	-	-	-	-	-	NN	-	-	NN	-	-	-	-	-	-	-	-	-	-	-	-	-	-	-	-	-
AX-147265753	#N/D	#N/D	2sten	2sten	2sten	2sten	2sten	2sten	2sten	2sten	-	-	-	-	-	-	-	-	-	-	-	-	-	-	-	-	-	-	-	-	-	-	-	-	-	-
AX-147265751	#N/D	#N/D	2sten	2sten	2sten	2sten	2sten	2sten	2sten	2sten	-	-	-	-	-	-	-	-	-	-	-	-	-	-	-	-	-	-	-	-	-	-	-	-	-	-
AX-177637537	#N/D	#N/D	2sten	2sten	2sten	2sten	2sten	2sten	2sten	2sten	-	-	-	-	-	-	-	-	-	-	-	-	-	-	-	-	-	-	-	-	-	-	-	-	-	-
AX-177637307	#N/D	#N/D	2sten	2sten	2sten	2sten	2sten	2sten	2sten	2sten	-	-	-	-	-	-	-	-	-	-	-	-	-	-	-	-	-	-	-	-	-	-	-	-	-	-

NN = No calls. 1sten and 2sten corresponds respectively to the identification of one and two alleles of *A. stenosperma*. ND = Not determined.
